# Active crustal straining and fault reactivation at Valles Marineris

**DOI:** 10.1038/s41467-026-78193-6

**Published:** 2026-10-15

**Authors:** Jung-Hun Song, Doyeon Kim, Folarin Kolawole, Joel M. Davis, Rebekah Parry, Ross Maguire

**Affiliations:** 1 Department of Earth Science and Engineering, Imperial College London, South Kensington Campus, London, UK; 2 Department of Earth and Environmental Sciences, Columbia University, New York, NY USA; 3 Department of Earth Science and Environmental Change, University of Illinois Urbana-Champaign, Urbana, IL USA

**Keywords:** Seismology, Structural geology, Tectonics

## Abstract

Tectonic deformation on stagnant-lid planets reflects the interaction between long-lived lithospheric structures and evolving stress fields. Whether the ancient tectonic fabrics can localise present-day seismic failure under contemporary stress fields remains poorly tested by direct geophysical observations. Here we show that the farside seismic event, S0976a, detected by InSight in the western hemisphere is a marsquake that occurred in Valles Marineris and involved strike-slip faulting in the deep crust. The stress orientation inferred from the focal mechanism is consistent with reactivation of pre-existing fault systems under a present-day stress field shaped primarily by long-term planetary cooling, regional Tharsis-related loading, and surface erosion-related local unloading. These observations provide direct seismic evidence that inherited fault systems on Mars can remain mechanically favourable for reactivation over billion-year timescales, suggesting that slip on deeply rooted faults remains a key mechanism of long-term strain release in a cooling single-lid planet.

## Abstract (web summary)

Valles Marineris, the Solar System’s largest canyon, is tectonically active today. A marsquake beneath it was traced to deep fault slip, showing billion-year-old faults can still rupture on Mars.

## Introduction

Tectonic longevity on rocky planets depends on how lithospheric stresses are generated, modified, and accommodated through time. On Earth, tectonic stresses are continually produced and dissipated through global plate interactions, with deformation focused along long-lived zones of weakness^1^. In contrast, stagnant-lid planets lack global lithospheric recycling, and stresses are generated primarily by non-plate tectonic processes such as global contraction, lithospheric flexure, mantle convection, magmatic activity, and volcanic loading^2,3^. On Mars, geologically young faults and ongoing seismicity^4–7^ suggest that deformation can persist well beyond the planet’s early evolutionary history^8^, likely driven by localised or regionally variable forcing^9,10^. At the same time, ancient tectonic structures and heterogeneous, near-critical lithospheric stress states may remain preserved, leaving inherited faults susceptible to reactivation by subsequent or ongoing stress sources. However, during plate-cooling phases in intraplate environments, pre-existing faults may undergo mechanical healing that involves geochemical reactions, resulting in progressive frictional strengthening of the faults^11^. Thus, whether the pre-existing tectonic fabrics of cooling single-lid planets can localise failure under later stress perturbations billions of years after their formation has not yet been directly demonstrated by geophysical observations.

Valles Marineris (VM), the largest tectonic structure on Mars, preserves a record of lithospheric stresses linked to the formation of the Tharsis rise^12,13^. It therefore provides a natural setting in which to assess how inherited fault architecture interacts with present-day lithospheric forcing. The formation and early deformation of VM have been attributed to extensional rifting^14,15^, gravitational collapse^16,17^, and transtensional deformation^18^, while geodynamic models suggest that long-wavelength stresses generated by Tharsis loading could have localised subsidence and shear strain across the surrounding lithosphere^19–21^. Orbital observations of reactivated faults^22^, landslides^4^, and syn-tectonic deposits^23^ indicate deformation continued after initial canyon formation^4,24^; however, widespread resurfacing and modification obscure primordial tectonic structures^25,26^, leaving their relationship to later deformation difficult to resolve from surface geology alone.

**Fig. 1:**
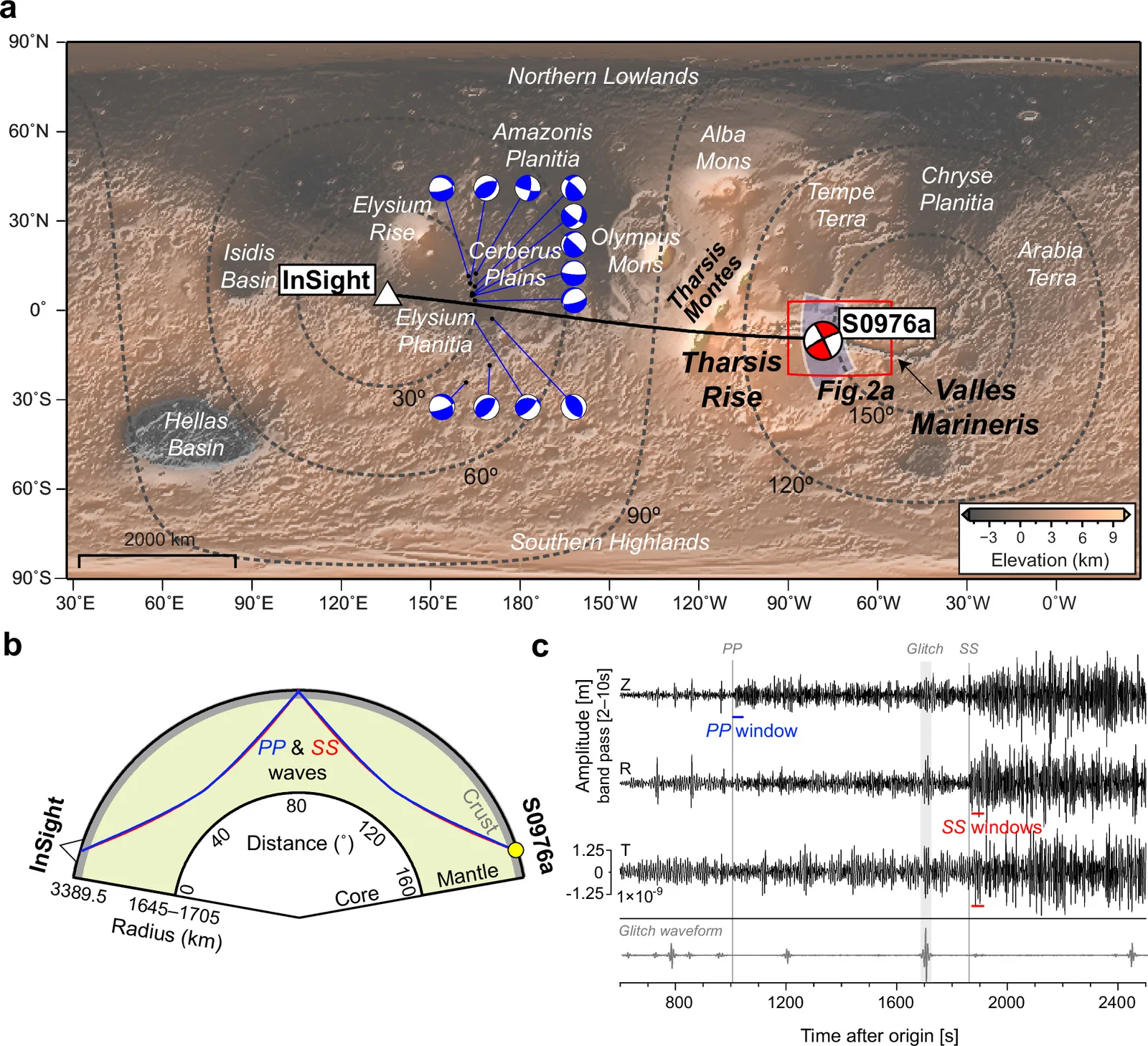
Location and seismic waveforms of event S0976a. **a** Mars Orbiter Laser Altimeter (MOLA) topography^113^ showing the InSight lander (white triangle), S0976a (red beachball), and the source–receiver great-circle path (black). Blue beachballs show previously published focal mechanisms^35,37,40^, whereas the red beachball shows the focal mechanism of S0976a determined in this study. The shaded region around S0976a denotes the epicentral uncertainty reported in the MQS catalogue^38^. The scale bar represents 2000 km at the equator. **b** PP and SS free-surface multiple ray paths for S0976a. **c** Filtered, deglitched three-component Very Broad-Band (VBB) displacement seismograms recorded by InSight. Coloured bars around PP and SS arrivals indicate analysis windows unrelated to glitches shown below.

Direct observation of present-day tectonic activity on Mars became possible with the InSight mission^27,28^, which provided the first successful seismic observations^6^ and new constraints on Martian interior structure (Lognonné et al.^29^ and references therein). Although InSight operated only a single seismic station^30^, high-quality event recordings have enabled estimates of source type^31,32^, marsquake magnitude^33^, faulting mechanism^34,35^, and rupture processes^9,36^. Detailed event characterisation has so far been largely limited to source regions relatively close to the InSight landing site (Fig. 1a), notably Cerberus Fossae^9^, and the source region of the mission’s largest marsquake (S1222a) in southern Elysium Planitia, near the equatorial dichotomy boundary^37^.

These previous studies demonstrate that even single-station seismic observations can constrain marsquake source mechanisms and their tectonic context, thereby motivating comparable analysis for VM. A seismic event recorded on Sol 976 (25 August 2021), hereafter S0976a, is located in western VM near Ius Chasma^38^, and represents the only marsquake detected in the western hemisphere. This event provides the first opportunity to bring seismological constraints to bear on VM and to test tectonic inferences about its deformation.

Here, we investigate the origin of S0976a and assess whether it was generated by faulting within VM or by an alternative process, such as meteoroid impact or landsliding. We show that the seismic radiation pattern is best explained by strike-slip faulting at crustal depth beneath western VM. We combine seismic analysis with structural interpretation of mapped fault systems and high-resolution orbital observations of pre-existing boulder tracks suggestive of regional susceptibility to seismic shaking. Together, these observations provide converging geophysical and geological evidence that VM remains tectonically active today, with seismicity localised by inherited fault systems reactivated under contemporary lithospheric stress fields.

## Results and discussion

### Seismic source characterisation of Marsquake S0976a

Constraining the source mechanism requires sampling the source radiation pattern over a range of azimuths and take-off angles to resolve fault orientation and slip direction. On Earth, this is commonly achieved using P-wave first-motion polarities together with amplitude information from dense seismic networks^39^. InSight, by contrast, operated as a single station and thus sampled the radiation pattern only sparsely. Previous single-station analyses on Mars nevertheless show that source mechanisms can still be constrained from three-component recordings by combining multiple seismic phases with their relative amplitudes and polarities^34,35,37,40,41^. Because different seismic phases sample distinct take-off angles, they provide complementary constraints on the source mechanism.

**Fig. 2:**
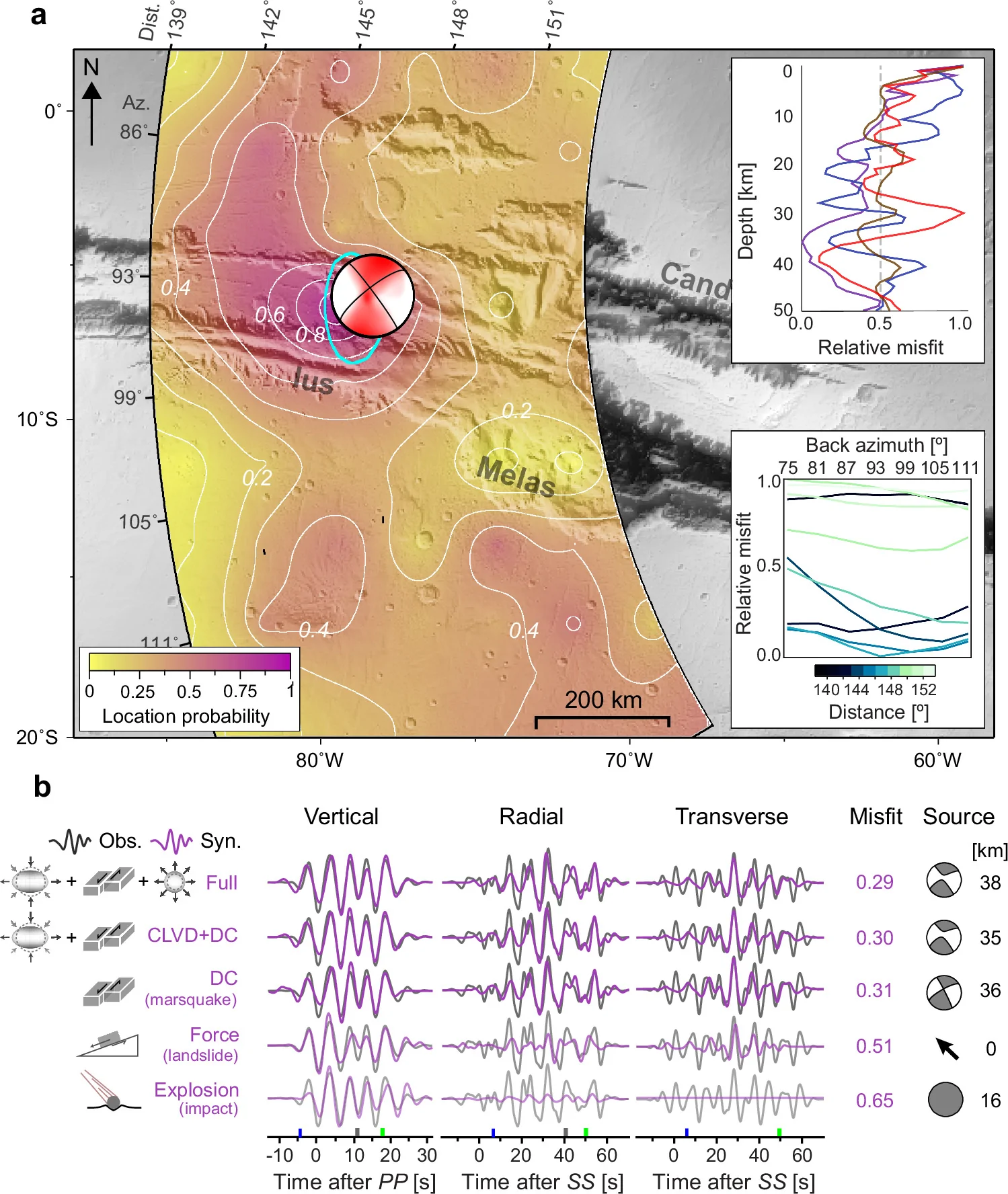
Seismic source characterisation of Marsquake S0976a. **a** Inferred focal mechanism solution and source-location probability. The beachball diagram shows an aggregate representation of the acceptable-solution ensemble, with its finite spread reflecting uncertainty and trade-offs among source parameters. Lines indicate the two nodal planes of the best-fit solution. Contours denote the joint probability from the product of waveform-fit and mapped fault density^50^ (see “Methods”). The region of maximum probability overlaps the independently constrained source area from frequency-dependent polarisation analysis of the high-frequency PP wave^52,53^ (cyan; Supplementary Fig. 19). Insets show relative misfit as a function of depth for different Martian interior models (top right;^43–46^), with colours denoting individual models as detailed in Supplementary Fig. 12, and as a function of back azimuth and epicentral distance (bottom right). **b** Best-fitting waveform comparisons between observed (black) and synthetic (coloured) three-component seismograms for the source-scenario tests. Synthetic waveforms were calculated using the Khan et al. model^43^. Values at right indicate waveform misfit and, where applicable, centroid depth and source mechanism. From top to bottom: full moment tensor inversion, including isotropic (ISO), double-couple (DC), and compensated linear vector dipole (CLVD); CLVD + DC; DC-only; force-based landslide model; and explosion, representing an impact-like source. The preferred solution has a dominant DC component, consistent with shear slip on a planar fault and strike-slip faulting. Vertical ticks mark the predicted arrivals of the principal PP/SS phases and possible depth phases, including pPP/sPP and pSS/sSS. For each phase group, only the earliest (first-arriving) predicted arrival is shown as a representative pick (Supplementary Fig. 13). Time is referenced to the PP and SS picks of Li et al. ^45^.

For S0976a, we jointly invert the first-arriving P and S phases, corresponding to the free-surface multiples PP and SS^38^, to constrain the source mechanism and associated fault-plane geometry, including strike, dip and rake (see “Methods”; Supplementary Fig. 1). Tests on a terrestrial reference event recorded at a single station show that inversion of PP and SS phases recovers known source parameters comparably to direct P and S, supporting the application of this approach to S0976a (Supplementary Fig. 11). Our results indicate a predominantly double-couple, strike-slip mechanism, with two steeply dipping nodal planes (Fig. 2). The best-fitting focal mechanism yields fault strikes of ~60° and ~330°. Its pressure axis (P axis) is oriented subparallel to the VM trough system, whereas the tension axis (T axis) is broadly perpendicular to it, reflecting horizontal extension across the canyon combined with shear parallel to its axis. The corresponding centroid depth lies within the middle-to-lower crust, above the estimated Moho depth of ~55–65 km beneath western VM^42^. Across the range of tested crustal structures and velocity models^43–46^, the allowed depth ranges are ~15–45, ~20–40, ~25–45, and ~15–40 km, respectively (Fig. 2 and Supplementary Fig. 12).

**Fig. 3:**
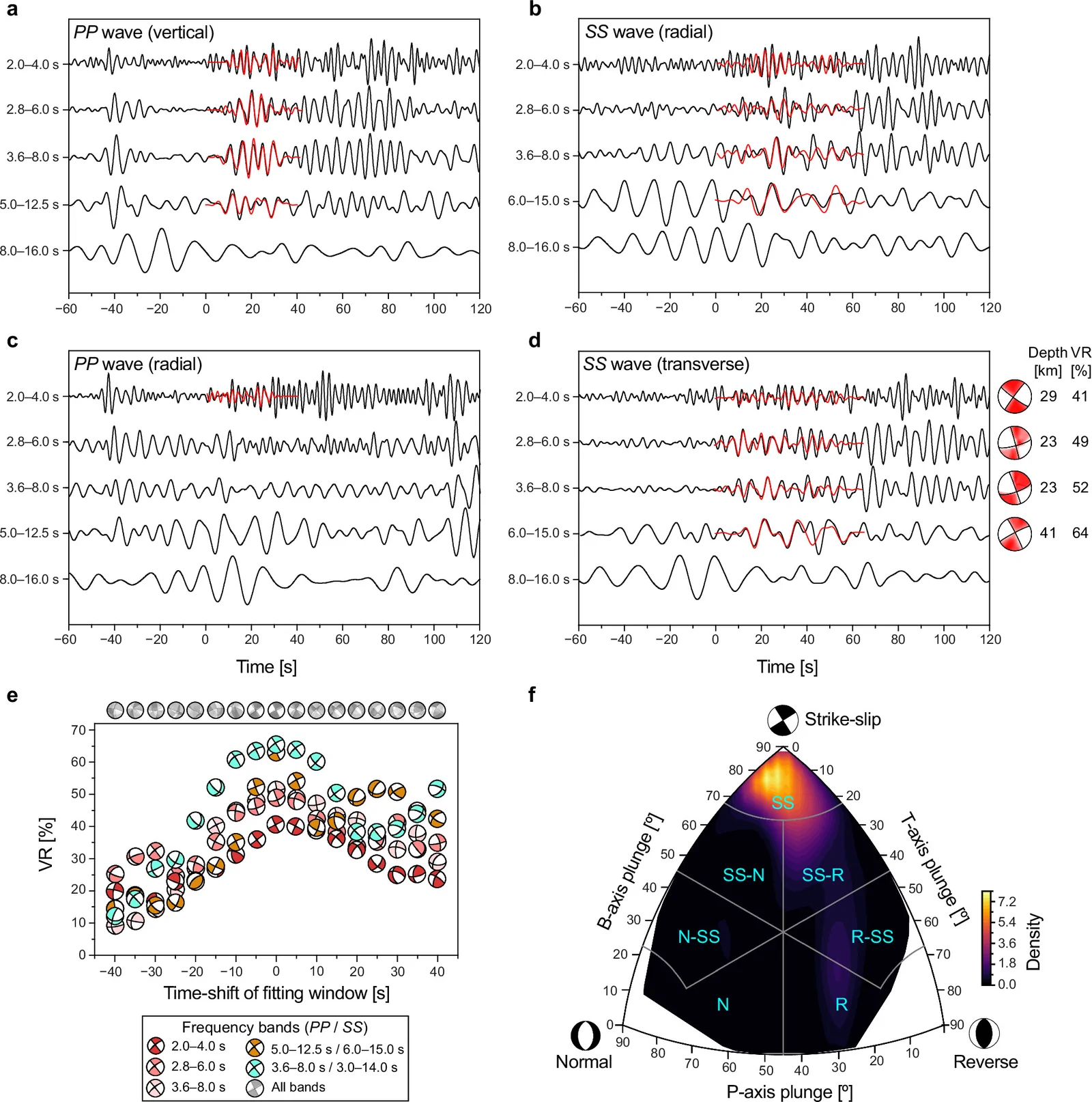
Sensitivity analysis of focal mechanism solutions to frequency band and fitting-window selection, and distribution of acceptable source types. **a–d** Bandpass-filtered PP and SS waveforms and corresponding fits for independent inversions using distinct frequency bands. Vertical (**a**) and radial (**c**) components are shown for PP, and radial (**b**) and transverse (**d**) components for SS. Observed and synthetic seismograms are shown in black and red. Rows correspond to period bands of 2–4, 2.8–6.0, and 3.6–8.0 s, followed by longer-period cases (5–12.5 s for PP; 6–15 s for SS; and 8–16 s for both phases). Inversions jointly fit PP and SS phases; synthetics are shown only for the bands and components included in each inversion. The preferred focal mechanism, centroid depth, and variance reduction (VR) are indicated at right, where the mechanism represents the ensemble average of solutions with VR > 0.9 × VRmax, where VRmax is the maximum variance reduction obtained among all grid-search solutions. Time is referenced to aligned synthetics, with 0 s marking the start of the inversion window. Delayed windowing of the long-period SS band (6–15 s) to avoid noise near the theoretical arrival causes an apparent advance of the observed waveforms. Waveform amplitudes are normalised by the maximum amplitude of the corresponding observed waveform. **e** Focal mechanism solutions and corresponding VR values for systematically shifted fitting windows relative to the preferred PP–SS alignment. Coloured focal mechanisms denote solutions obtained for the tested frequency bands shown in (**a**–**d**), using the preferred centroid depth for each band. Grey, semi-transparent focal mechanisms shown above the panel represent the cumulative ensemble of solutions across all tested frequency bands. **f** Distribution of acceptable solutions projected onto the Kaverina diagram^88^. SS strike-slip; SS-N strike-slip with normal component; SS-R strike-slip with reverse component; N normal; R reverse; N-SS normal with strike-slip component; R-SS reverse with strike-slip component.

We assess the robustness of the inferred mechanism by exploring the full solution ensemble across tested source distances, depths, velocity models, and attenuation parameters using waveform variance reduction (see “Methods”). At the preferred epicentral location, the P and T axes cluster tightly across the explored depth range (Supplementary Figs. 2 and 3). Small shifts in source distance reduce the maximum variance reduction by more than 20% (Fig. 2, Supplementary Figs. 13 and 14) and visibly degrade the waveform fit to the PP and SS phases (Supplementary Fig. 14). Away from the preferred location, the corresponding ensembles become diffuse, indicating that no comparably stable fault geometry is recovered. Strike, dip and rake are similarly well constrained, with even small perturbations producing a noticeable reduction in waveform fit (Supplementary Fig. 1). The strike-slip solution for S0976a is also stable across the available frequency content (Fig. 3a–d) and different fitting-window positions, with the highest VR obtained when the windows are centred on the observed PP and SS energy (Fig. 3e). Including the SKS phase (Supplementary Fig. 15) does not improve the overall waveform misfit, given the low signal-to-noise ratio of the SKS arrival in S0976a^47^. The inferred mechanism is likewise insensitive to the tested velocity-model choices or attenuation parameters (Supplementary Fig. 12), which are derived from Martian teleseismic estimates at periods near 15 s^28,48^ and are appropriate for modelling S0976a as a low-frequency event^49^. The crustal velocity structure derived from Rayleigh-wave group-velocity dispersion^46^ likewise favours a steeply dipping strike-slip mechanism, but provides a poorer fit to parts of the later PP and SS waveforms than velocity structures containing intracrustal interfaces, suggesting that these interfaces contribute to the observed waveform complexity. Among all acceptable solutions from the full strike–dip–rake grid search across candidate source locations, depths and scalar moments (see “Methods”), 82.2% fall within the strike-slip family (Fig. 3f and Supplementary Table 1), dominated by steeply dipping strike-slip to oblique strike-slip mechanisms. Within this ensemble, nodal-plane strike orientations, folded into the 0–180° range for statistical summary, cluster at 48.0° ± 21.5° and 143.5° ± 22.5°, with steep dips centred near 80° (Supplementary Table 1 and Supplementary Fig. 4). The corresponding rakes for the two strike-orientation clusters are centred at 8.0° ± 26.0° and 174.0° ± 24.5°, close to pure strike slip. The expected back-azimuth–strike trade-off permits an outlying local best-fit solution with a VM-parallel nodal plane (Supplementary Fig. 3), while the ensemble as a whole consists predominantly of strike-slip mechanisms with nodal planes oblique to VM. An independent amplitude-ratio method^35^ yields a comparable strike-slip mechanism, although these solutions retain the expected fault-plane ambiguity associated with single-station amplitude methods (Supplementary Fig. 16). We therefore use the waveform-fitting solution for interpretation.

Although single-station geometry permits some trade-off between back azimuth and fault strike^37^, the stable solution ensemble remains confined to western VM. Within western VM, the spatial density of previously mapped faults, used here as a first-order proxy for regions prone to seismicity^50^, provides an independent constraint on the location and points to a source near Ius Chasma (see “Methods”; Fig. 2a). In this region, mapped surface structures likely mark the surface expression of broader crustal deformation zones associated with long-term lithospheric extension and strain localisation^20^. We note that this weighting is approximate, as fault density informs the regional distribution of deformation rather than directly constraining the geometry of deep seismic slip. Tectonic structures outside the canyon system are more commonly characterised by contractional landforms, including wrinkle ridges^51^, with possible localised extensional deformation. Despite these caveats, the highest joint probability lies within a structurally complex area near Ius Chasma, where dense graben, normal faults, and oblique structural fabrics define a tectonic setting consistent with the inferred strike-slip mechanism. This location also coincides with the back-azimuth estimates from our frequency-dependent polarisation analysis^52,53^ of the high-frequency PP wave (cyan contour in Fig. 2a, Supplementary Figs. 18 and 19).

S0976a is incompatible with any plausible non-tectonic source process. Source representations of meteoroid impacts, landslides, and other mass-wasting processes, including single-force, multiple-force time series, isotropic and mixed non-double-couple mechanisms, all fail to reproduce the observed phase polarities, arrival times, and relative amplitudes of the PP and SS waves (Fig. 2b). In particular, force-based source models cannot account for the strong horizontal ground motion observed in the data. This is consistent with the predominantly low-frequency content of S0976a, which contrasts with the high-frequency signatures typically observed for confirmed meteoroid impacts^31,54^. Allowing additional volumetric or tensile terms improves the waveform fit by less than 2%, suggesting that the source is adequately described by shear slip on a planar fault.

Consistent with the seismic evidence for a tectonic source, post-2021 Context Camera (CTX)^55^ images show no contemporaneous landslides, fresh impact craters or other surface disturbances within the inferred source region (see “Methods”). Given the widespread evidence for slope failure in VM, including abundant landslide deposits^26,56^, we examined all available post-event CTX images for signs of recent collapse that could have generated S0976a, but found none within the available temporal coverage (Supplementary Fig. 20). Based on a simple scaling estimate, generating a seismic event the size of S0976a by landslide on Mars would require a failure volume of at least 10^7^ m^3^ (see “Methods”). Such a collapse should have left a fresh deposit several hundred metres to a kilometre across, detectable in CTX images, making a landslide origin unlikely.

The inferred tectonic origin of S0976a raises the possibility of detectable aftershocks. No aftershocks were recorded in the days to weeks following S0976a. The next catalogued event^57^ occurred 36 sols later at an epicentral distance of ~38°, far closer than the teleseismic distance of S0976a. Most events recorded after S0976a until the end of the InSight mission (~440 sols later) fall within ~29–38°, the distance range typical of Cerberus Fossae events. The only later event beyond 100°, S1000a, is a confirmed meteoroid impact, with a corresponding fresh crater identified in HiRISE images and spectral content consistent with other observed impacts^31^.

**Fig. 4:**
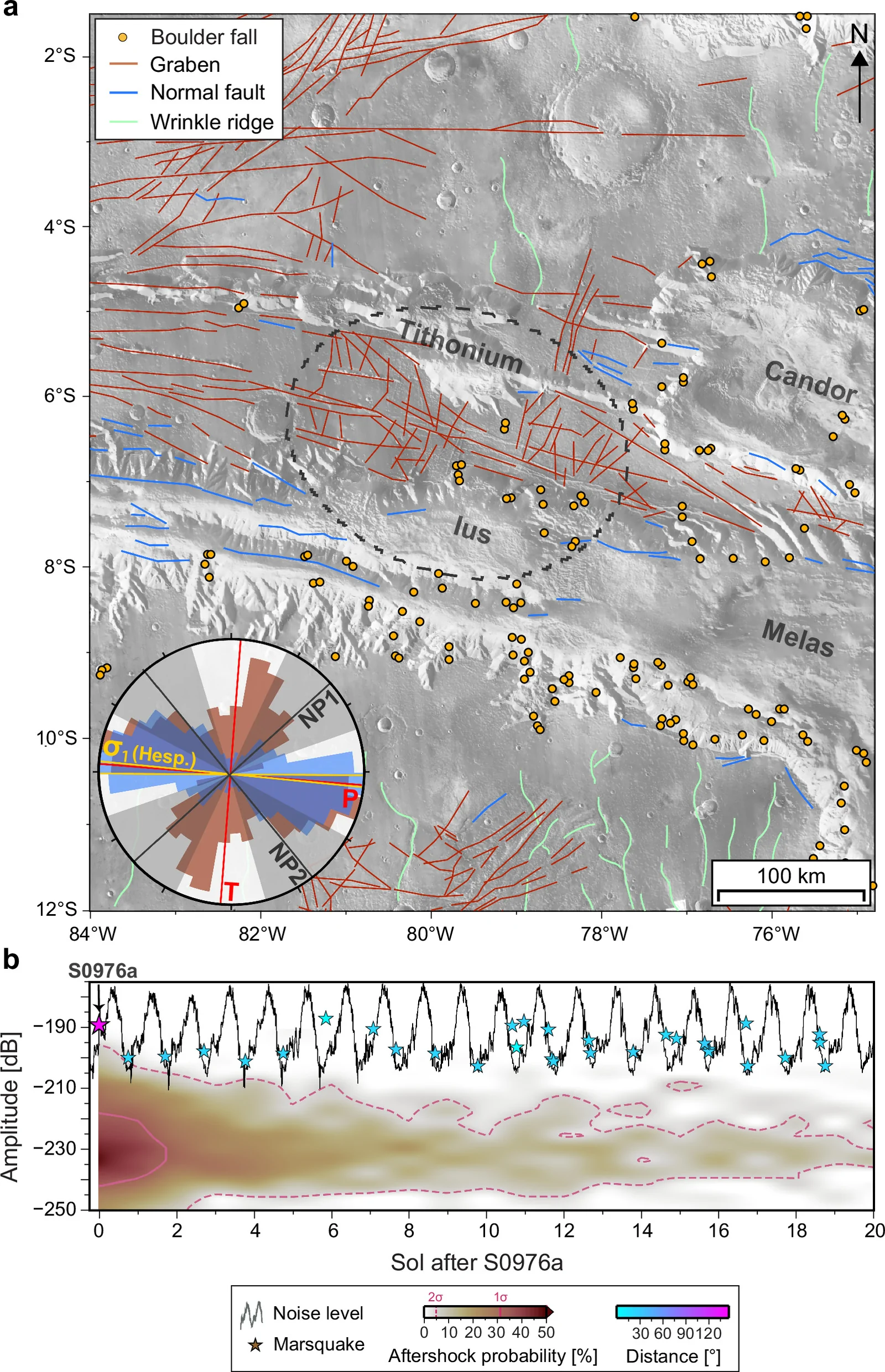
Fault map of the S0976a marsquake source in western Valles Marineris and aftershock detectability. **a** Global Context Camera (CTX) mosaic of the inferred source region, defined by the probability *>*0.6 in Fig. 2a (black dashed), together with mapped tectonic features, including graben, normal faults, wrinkle ridges and boulder-fall sites. The rose diagram shows the feature-count distributions for graben populations (brown) and normal faults (blue), together with the median P- and T-axis orientations (red) and the nodal planes (black, NP1 and NP2) derived from the acceptable focal mechanism solution ensemble. Grey fans indicate the angular ranges of the nodal planes within the focal mechanism solution ensemble (see “Methods”). The orange line marks the inferred E–W to WNW–ESE (~090–100°) orientation of the maximum principal compressive stress associated with Hesperian-aged deformation^59^. A broader structural map of Valles Marineris is provided in Supplementary Fig. 22. **b** Cumulative probability of simulated aftershock occurrence in time–amplitude space, based on synthetic marsquake waveforms at the landing site, with 33% and 5% probability contours (dashed and solid magenta, respectively). The black curve denotes the root-mean-square ambient noise level at the lander. Catalogued marsquakes are shown as stars, coloured by distance.

The absence of additional catalogued events from the source region could reflect intrinsically low aftershock productivity^2,3^, limited detectability at teleseismic distances, or both. Synthetic waveforms from stochastic aftershock simulations that account for a range of source and propagation effects (see “Methods”; Fig. 4) indicate that ground displacements generated by a marsquake in western VM would rarely exceed the ambient noise level at the lander (Fig. 4b). Even under conservative assumptions for b-values and aftershock productivity, most events would fall below the detection threshold. The probability of detecting even a single sizeable aftershock within 50 sols is only 0.1%, with any additional detections thereafter remaining unlikely. This low detection probability is explained by geometric limitations on long-distance wave propagation across Mars, including core shadowing^58^ and attenuation by deep interior structure^44^, which reduce the observability of seismic phases from distant source regions such as VM.

### The S0976a event region in Valles Marineris

To assess the structural context of the S0976a event region, we mapped structural features across western VM (Fig. 4a), spanning the full range of permissible epicentral locations. Using the fault mapping of Kumar et al.^4^ as a guide, we re-evaluated structural features by digitising normal faults, grabens, wrinkle ridges, and boulder-fall sites from high-resolution CTX images (Supplementary Fig. 22). Here, grabens are defined as fault-bounded troughs expressed by paired normal-fault scarps, whereas individual normal faults are mapped where only a single scarp can be identified in the available imagery. This mapping reveals a bimodal graben population with dominant strikes at ~15° and ~115°, defining an orthogonal fault system (Fig. 4a). Normal faults are predominantly oriented subparallel to the VM axis. Near the approximate S0976a source location, on the plateaus between northern Ius, Tithonium, and Candor Chasmata, grabens form dense intersections of well-developed orthogonal sets consistent with these regional trends. The estimated strikes of the possible coseismic rupture planes fall within the range of mapped graben and normal fault orientations, but do not uniquely coincide with a single dominant surface faulting trend. The P and T axes, however, align with the dominant structural fabric orientations: the P axis trends subparallel to VM and to the dominant WNW–ESE graben and fault set, whereas the T axis is approximately orthogonal to it and aligns with the secondary NNE–SSW set (Fig. 4a, inset). Although local geomorphic patterns occasionally suggest minor lateral displacements, these features are sparse and discontinuous. At the regional scale, orbital analyses have identified through-going fault zones and offset features indicating cumulative strike-slip deformation in VM, including left-lateral faulting in Ius Chasma^18^ and shear-zone-related deformation in Ophir and Hebes Chasmata^22^. These observations point to a significant oblique component in the tectonic evolution of VM. The presence of an orthogonal structural fabric near the inferred source region may therefore reflect regional-scale strike-slip tectonism, a polyphase deformation history, or localised oblique deformation within a rift transfer zone.

### Tectonic stresses and fault reactivation in Valles Marineris

The inferred present-day stress geometry is inconsistent with models for the formation of VM that invoke gravitational collapse of overthickened crust, which predict dominantly vertical loading and axisymmetric extension^24^. Instead, it requires a significant component of oblique slip, more consistent with a transtensional origin for VM and with mapped fault kinematics, oblique graben geometries, and asymmetric subsidence^18,59,60^ (Fig. 4). Kinematic analyses of the Hesperian transtensional evolution of VM suggest a broadly E–W-oriented maximum compressive axis of ~090–100°^59^, consistent with the present-day WNW–ESE P-axis orientation of ~95° inferred from the S0976a focal mechanism (Fig. 4a, inset). This agreement suggests that the orthogonal structural fabric may record long-lived strike-slip deformation, possibly facilitated by high strain-weakening rates in the ductile roots of crustal-scale faults^61^.

**Fig. 5:**
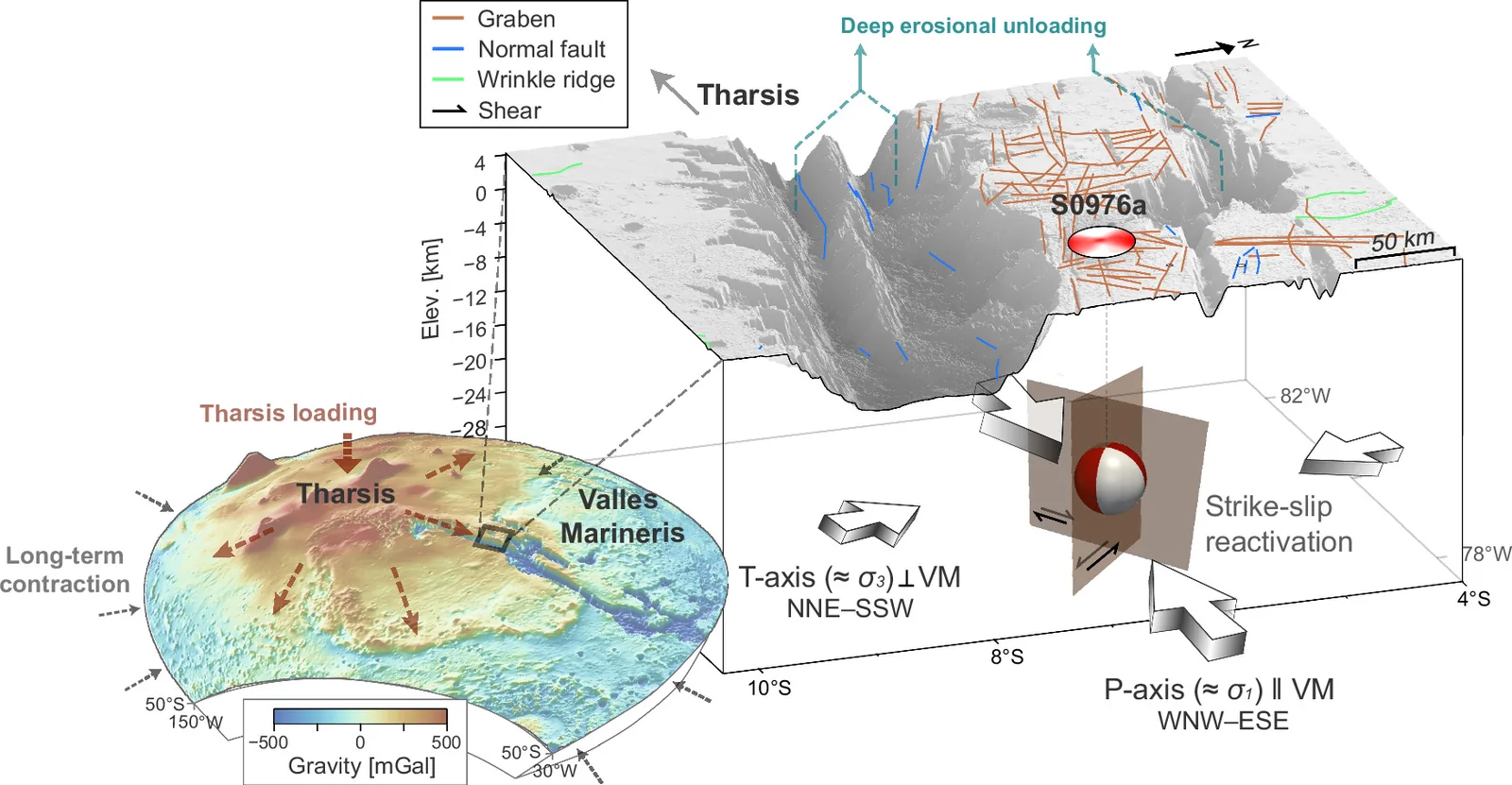
Schematic tectonic setting and inferred faulting geometry of the S0976a marsquake. Right, interpreted fault geometry from the focal solution and structural features mapped around the source region in Fig. 4. The S0976a focal mechanism is shown as a 3-D beachball, with both nodal planes (shaded) and associated slip directions (black arrows). The P axis is subparallel to VM, whereas the T axis is approximately perpendicular to it, consistent with dominantly strike-slip motion along VM fault fabrics. Left, regional topography and free-air gravity anomalies of the Tharsis rise and Valles Marineris^108^. A plausible interpretation invokes slip on suitably oriented VM faults under a combined lithospheric stress field shaped by long-term planetary contraction and regional Tharsis-related stresses, while local flexure, subsidence, dynamic support, and erosional unloading associated with deep canyon incision may further modify the regional crustal stress state.

The strike-slip mechanism of S0976a is therefore best interpreted as slip on inherited structural fabrics under the present-day stress field (Fig. 5). VM-parallel normal faults and grabens are oriented subparallel to the P axis and may be particularly susceptible to dilatant opening, while VM-oblique faults may accommodate strike-slip shear. The focal depths of S0976a, ~20–40 km, are deeper than the inferred shallow wrinkle-ridge detachments, <10 km^62,63^, although deeper-seated wrinkle-ridge faulting has also been proposed^64^. Transfer zones, such as the source region of S0976a, are strain relay zones that commonly host excess tectonic stress concentrations and localise earthquake ruptures^65,66^. Accordingly, shear displacement on rift-oblique or accommodation faults within VM is plausible where these structures are favourably oriented with respect to the present-day stress regime. NE-striking rift-oblique faults near the inferred epicentral region (Fig. 4a) may have originated as transfer zones between en echelon trough segments or during an earlier phase of oblique extension, making them mechanically favourable sites for seismogenic strain localisation under the current stress field. Thus, the resolved modern strike-slip stress regime suggests that strain is accommodated primarily by reactivation of pre-existing structures rather than by the formation of new faults^67–69^.

Several present-day stress sources may account for the strike-slip character of S0976a without requiring regional extension to dominate the Martian stress field^70^. Long-term planetary cooling provides a background horizontal compression, whereas Tharsis-induced flexural and long-wavelength lithospheric loading stresses could modify both the magnitude and orientation of the horizontal principal stresses, permitting local conditions conducive to seismic slip on suitably oriented faults^71^ (Fig. 5). At the inferred source depth of S0976a, the basaltic crust would be subject to lithostatic pressures on the order of several hundred MPa. This confining pressure would tend to suppress tensile opening and favour brittle shear failure^71^, consistent with a near strike-slip stress state in which a horizontal σ_1_ is imposed primarily by global contraction, while regional Tharsis-related stresses preferentially reduce N–S horizontal compression, making this the σ_3_ direction.

Candidate mechanisms for locally reducing σ_3_ include continued flexural adjustment of the Tharsis load and deeper buoyancy- or dynamically supported uplift beneath Tharsis, and deeply incisive surface erosion of VM. Recent gravity–topography models suggest a deep positive-buoyancy anomaly that contributes ~20% of present Tharsis uplift^72^. Flexural bending stresses associated with lithospheric adjustment are expected to locally modulate horizontal compression near VM^21^, while long-wavelength support of the Tharsis rise may further reduce circumferential compressive stress. Regional stress perturbations associated with VM tectonic architecture may also influence the local stress field and fault reactivation. Deep canyon incision through surface erosion of VM could have created an unloading effect, which would decrease the vertical stress and result in horizontal compression in the crust. In these interpretations, the alignment of the S0976a T axis with mapped graben and ancient normal-fault fabrics near Ius Chasma implies that present-day crustal extension or transtension across VM is not pervasive. Rather, it indicates that inherited VM-parallel normal faults are favourably oriented for dilatant opening, whereas rift-oblique structures remain capable of accommodating the dominant strike-slip shear. Given the modest seismic moment inferred from our waveform modelling of S0976a, limited slip on a pre-existing fault patch could produce plausible MPa-scale stress drops without nucleating a new through-going fault. This interpretation is consistent with lunar stress-superposition models^73^, predicting that long-lived contractional stresses bring inherited structures close to failure, while secondary stresses—here non-tidal—determine which faults are reactivated. Although the relative contributions of flexural loading and dynamic support cannot be uniquely determined, the stress orientation recorded by S0976a provides observational evidence against which geodynamic models of their combined influence on the present-day stress field can be tested.

Thermal and rheological variations within the Martian lithosphere may produce spatial variations in mechanical strength^74^, while pre-existing fault zones can provide mechanically weak surfaces that focus later seismic deformation under slowly evolving lithospheric stress fields. Numerical models predict that stress maxima associated with lithospheric flexure evolve only gradually as the lithosphere cools and thickens, enabling repeated fault reactivation without major changes in the large-scale stress field^12,20,21^. Similar localisation of seismicity beneath Cerberus Fossae^9^ suggests that Martian tectonic activity may be concentrated within a limited number of long-lived deformation zones. However, the limited number of well-constrained source regions and focal mechanisms may also reflect Mars’ low seismic moment rate^75^, InSight’s short single-station record, and poor detectability of small distant events, rather than an absence of seismicity elsewhere.

### Implications for stagnant-lid tectonics on Mars

InSight seismic observations^40^ from three tectonically distinct regions now reveal contrasting styles of present-day deformation within the Martian lithosphere: strike-slip faulting beneath VM constrained here from S0976a, compressional faulting near the crustal dichotomy in southern Elysium Planitia inferred from S1222a^37^, and normal faulting beneath Cerberus Fossae^9^. The coexistence of these mechanisms indicates that deformation is partitioned across a laterally heterogeneous stagnant lid, rather than governed by a globally uniform stress field. This partitioning implies that broad lithospheric stresses act on a mechanically heterogeneous shell, shaped by long-lived structural and rheological variations. In VM, the WNW–ESE maximum compressive stress axis inferred from S0976a is broadly consistent with the axis of maximum horizontal stress proposed for the Hesperian development of the canyon system, linking present-day seismic failure to a fault architecture shaped during earlier Tharsis-related deformation.

Deep brittle faulting beneath western VM indicates that, at least locally, Martian lithosphere remains mechanically active despite extremely low strain rates. Such behaviour suggests that mechanically strong domains can persist within a laterally heterogeneous stagnant lid, allowing inherited structures to localise later deformation over geological timescales^76,77^. Limited viscous relaxation allows lithospheric stress heterogeneity to be maintained^21^, promoting renewed slip on pre-existing structures^78,79^. This interpretation is supported by independent constraints, including gravity–topography relationships indicative of flexural support^76^, and the clustering of marsquakes within localised deformation zones^9^. In this setting, deformation reflects selective strain release along favourably oriented structures under long-wavelength lithospheric stresses. In a stagnant-lid lithosphere, slow healing of fault zones and slowly evolving stress fields may jointly control where tectonic activity continues long after the structures themselves were first established^11,80^.

Plate tectonics appears to be rare among rocky planets, with most known terrestrial bodies instead inferred to operate in a stagnant-lid regime^81^. Where viscous relaxation is inefficient and convective stresses remain small relative to lithospheric strength^81,82^, earlier tectonic events can leave behind heterogeneous structural and mechanical conditions that shape later deformation. Under these conditions, seismicity may preserve a mechanical imprint of earlier tectonism through preferential slip along older weaknesses. More broadly, our observations here suggest that tectonic persistence on rocky planets without plate tectonics may reflect the interaction between long-lived fault architecture and contemporary lithospheric forcing^81^, allowing geologically ancient structures to remain seismically active over planetary timescales.

## Methods

### Seismic characteristics of S0976a

Marsquake S0976a was recorded on sol 976 of the InSight mission and classified as a low-frequency (LF) event by the Marsquake Service^57^. The event occurred at an epicentral distance of approximately 146° ± 7° and a back azimuth of 101° ± 25° from the InSight lander^38^. Despite its moderate magnitude, S0976a exhibits well-resolved P, SV, and SH body-wave phases, providing a rare opportunity to directly investigate present-day deformation beneath VM. The waveform is characterised by emergent yet distinct PP and SS phases with stable polarities across the vertical and horizontal components (Fig. 1c). The amplitudes and polarities of the PP and SS phases, together with later arrivals (e.g., depth phases), provide strong constraints on fault geometry and source depth.

### Single-station body-wave moment tensor inversion

We performed a single-station moment tensor inversion using PP and SS body-wave phases. Synthetic seismograms were computed with Instaseis^83^, based on Green’s functions generated using the spectral-element method (AxiSEM^84^) and one-dimensional (1-D) Martian interior models. Broadband wavefields (periods down to 2 s) were simulated in axisymmetric media using a 2-D spectral-element scheme with analytical reconstruction of the azimuthal dimension via multipole expansion^84^. Attenuation was parametrised using a frequency-independent shear quality factor in the crust and upper mantle^28,44^. The observed VBB displacement records were deglitched/despiked^85^, demeaned, detrended, tapered, and rotated into vertical, radial and transverse components for each tested source back azimuth. The inversion combines linear least-squares fitting with a nonlinear grid search over source depth, strike, dip, rake, and static time shifts^86^.

For the preferred inversion, the vertical PP waveform was filtered in the 3.6–8.0 s period band, whereas the radial and transverse SS waveforms were filtered in the 3.0–14.0 s period band. Waveforms were processed using a one-pass causal bandpass filter together with an additional high-pass filter at ~11 s period to suppress long-period pre-arrival noise. These passbands were selected because they provided the best joint fit across the PP and radial/transverse SS components among the tested frequency ranges (Fig. 3). Shorter-period bands retain higher signal-to-noise ratios but introduce greater waveform complexity associated with scattering and unmodelled three-dimensional crustal heterogeneity, whereas longer-period bands produce smoother waveforms but exclude part of the body-wave energy. Across all tested passbands, the strike-slip mechanism remained stable, indicating that the inferred source geometry is not dependent on a single frequency band choice. PP and SS phases were fit using extended windows (~45 s for PP and ~75 s for SS) selected to include the principal arrivals and possible depth phases, and static time shifts were restricted to ±10 s to avoid overfitting while remaining within picking uncertainties^37^. Only the vertical component was used for PP, whereas radial and transverse components were used for SS. The PP radial component was excluded because of its low signal-to-noise ratio (SNR), except for the 2–4 s period inversion (Fig. 3c), in which the PP radial arrival was discernible. Misfit was quantified using an amplitude-normalised *L*_2_ norm. Because the observed PP waveform has lower energy than the radial and transverse SS waveforms, the PP residual was scaled by the SS-to-PP energy ratio, giving PP and SS equal influence on the overall misfit. To test sensitivity to processing choices, we repeated the inversion across multiple PP and SS passbands spanning the observed source spectrum and systematically shifted the PP–SS fitting windows relative to the optimal alignment while keeping the window lengths fixed (Fig. 3a–e).

To account for source location uncertainty, we sampled epicentral distances of 146° ± 7° and back azimuths of 101° ± 25° on a regular grid. For each location, source depth was independently searched between 0 and 50 km in 1 km increments. Moment tensor parameters (strike, dip, and rake; each discretized into 60 values) and scalar moments (*M*_w_ 3.3–4.0 in increments of 0.1) define the full source parameter space (Supplementary Fig. 1). The preferred solution maximises the variance reduction^87^ (VR),

1 $${VR}=\left(1-\frac{{\sum }_{i}{({d}_{i}-{s}_{i})}^{2}}{{\sum }_{i}{d}_{i}^{2}}\right)\times 100,$$

where *d*_*i*_ and *s*_*i*_ are observed and synthetic displacements, respectively, summed over all time samples. For visualisation, we instead use the corresponding normalised *L*_2_ misfit, ${\sum }_{i}{({d}_{i}-{s}_{i})}^{2}/{\sum }_{i}{d}_{i}^{2}$, which provides a more intuitive representation of solution quality (Fig. 2). For SS, VR was computed separately for radial and transverse components and averaged, and combined with PP with equal weight. The reported depth ranges were defined as the interval over which the depth-dependent normalised misfit remained below 0.5. The normalised misfit was obtained by applying a common min–max scaling to the misfit values across all tested interior models.

For visualising the stability of stress-axis orientations, P- and T-axis directions were derived from each trial mechanism, represented as unit vectors, and projected onto a common hemisphere to resolve directional ambiguity. At individual candidate locations, VR-weighted axis-density fields were used to assess whether the waveform fit preferentially supports a stable mechanism (Supplementary Fig. 2). Locations and depths for which the waveform fit strongly favours a specific fault geometry yield concentrated P- and T-axis density maxima. In contrast, diffuse or blurred distributions indicate that acceptable solutions span a wide range of source parameters, including depth, and that VR does not preferentially favour a single mechanism, reflecting poor constraint at those locations (Supplementary Fig. 3).

To quantify uncertainty in the focal mechanism, we extracted an acceptable-solution ensemble from the full grid search rather than relying on a single best-fitting model. For each candidate source location (Supplementary Fig. 3), source depth and scalar moment, we calculated VR for all strike–dip–rake combinations. We then retained all solutions with VR > 0.9 × VRmax, where VRmax is the maximum variance reduction obtained across the explored source-parameter space. The focal mechanism shown in Fig. 2a is an aggregate representation of these acceptable solutions; its finite spread reflects the remaining uncertainty and trade-offs among source location, depth and focal-mechanism parameters.

We further characterised the acceptable-solution ensemble statistically. All accepted focal mechanisms were first projected onto the Kaverina source-type diagram^88^, which uses the relative orientations of the compressional (P), tensional (T) and null (B) axes to distinguish strike-slip, normal, reverse and mixed faulting styles. This classification was used to quantify the relative proportions of each source type within the acceptable-solution ensemble (Fig. 3f) and to assess whether the nodal-plane geometry could be interpreted primarily in terms of conjugate strike-slip fault orientations. Nodal-plane strike orientations were analysed as axial data, folding strikes into the 0–180° range and treating orientations separated by 180° as equivalent rather than as directed 0–360° strikes. We then separated the two dominant strike-orientation populations using k-means clustering and calculated circular means and circular standard deviations for each population in axial-data space (Supplementary Fig. 4). After assigning each nodal plane to a strike-orientation cluster, the associated dip and rake values from the original strike–dip–rake triplets were summarised using median values, percentile ranges and cluster statistics. These ensemble statistics are reported in Supplementary Table 1 and provide the uncertainty bounds used to assess whether the inferred strike-slip mechanism and nodal-plane orientations are stable across the acceptable solution space.

We performed synthetic recovery tests to evaluate whether the single-station inversion can reliably resolve focal mechanism type and source depth (Supplementary Figs. 5–9). Synthetic events with known mechanisms (strike-slip, normal, and reverse faulting) were placed at the S0976a source location and a fixed depth of 40 km, and processed using the same geometry, filtering, and inversion settings as the real data. Strike-slip scenarios were generated over a range of strike angles (0–67.5° in 22.5° increments), while normal and reverse faults were tested with fixed dip (45°) and variable strike. No additional noise was introduced in order to isolate the intrinsic resolving power of the single-station phase information. The inversion successfully recovered the correct faulting style and depth in all cases (Supplementary Fig. 5), demonstrating that the method robustly distinguishes between different focal mechanism types under the adopted configuration. As expected, azimuthal ambiguity remains, with comparable waveform fits obtained under joint rotation of source azimuth and fault strike (Supplementary Figs. 6–9), reflecting the fundamental limitation of single-station geometry^35,37^. Synthetic tests, including the radial component PP waveform, show that its inclusion does not substantially alter the source geometry or further constrain the acceptable solution space (Supplementary Fig. 10).

We further tested the resolving power of the single-station inversion by applying the same method to a terrestrial earthquake recorded under comparable source–receiver geometry (Supplementary Fig. 11). Despite differences in magnitude and frequency content, this provides an independent check on whether a unique solution can be recovered from single-station PP and SS phases within the same inversion scheme. We selected the 15 May 2020 Nevada earthquake (GCMT 202005151103A; *M*_*w*_ ≃ 6.5), recorded at an epicentral distance of ~145° at a single Global Seismic Network station in Australia (CAAN). Long-period (10–50 s) PP and SS phases were analysed on three components, and synthetics were computed using PREM^89^. The inversion recovers the correct faulting style and a source depth consistent with the reported solution, demonstrating that single-station body-wave inversion can robustly resolve faulting type and depth.

An additional sensitivity test of the interior structure was performed using five different 1-D Martian interior models developed in the context of the InSight mission: InSight KKS21_GP^58,90,91^, Khan et al.^43^, Samuel et al.^44^, Li et al.^45^, and Kim et al.^46^ (Supplementary Fig. 12). These models draw on complementary datasets and modelling approaches: KKS21_GP and Khan et al. combine InSight seismic observations with geophysical and mineral-physics constraints, Samuel et al. incorporate geodynamic constraints, Li et al. use underside-reflected seismic phases to resolve regional crustal structure, and Kim et al. use surface-wave dispersion to characterise regional crustal and uppermost-mantle structure. For each model, the inversion was repeated using the same parameterisation as adopted for the preferred analysis. For KKS21_GP, Khan et al., and Samuel et al. models, we used their corresponding 1-D average crust–mantle velocity profiles, adopting the shear quality factor (Qs) of Samuel et al. for the Khan et al. model. The Li et al. crustal model was combined with the Samuel et al. mantle structure, whereas the Kim et al. surface-wave model was joined to the Samuel et al. model below ~90 km. All tested models favour a strike-slip mechanism with steeply dipping fault planes (Supplementary Fig. 12) and yield preferred depths within 20–40 km (Fig. 2). The inversion yields *M*_*w*_ 3.4–3.6, somewhat lower than the MQS catalogue estimate^33^ of 4.0. Similar discrepancies between waveform-based moment tensor magnitudes and MQS magnitudes have been noted in previous studies of marsquakes^34,37,40^. This mismatch may reflect differences in the assumed radiation pattern used for catalogue magnitude estimation, as well as in the seismic velocity models used to estimate seismic moment^92^.

### Amplitude-based source analysis

As an independent validation, we applied the amplitude-only method^35^, which estimates source mechanisms using P, SV, and SH amplitude ratios (Supplementary Fig. 16). Because PP and SS phases undergo free-surface reflection, their waveforms are phase-shifted (approximately Hilbert transformed) and experience amplitude modifications relative to direct P and S waves^93^. To account for these effects, we generated synthetic waveforms for identical source mechanisms at both the full source–receiver distance of S0976a and at half that distance. Phase shifts and amplitude ratios were quantified by comparing PP and SS phases at the full distance with their corresponding direct P and S phases at half the distance, thereby isolating the effects of surface reflection and propagation. These corrections were then applied to the observed amplitudes, allowing the reflected phases to be treated as equivalent to direct P and S waves originating from the bounce point. The corresponding radiation patterns are preserved, as the take-off angles are equivalent. The resulting solutions consistently indicate strike-slip faulting with orientations comparable to those obtained from waveform inversion. As expected, solutions exhibit fault-plane ambiguity associated with rotation about the P or T axes, reflecting the reduced information content of amplitude-only methods^35^.

### Force-based source inversions for landslide scenarios

We modelled potential landslide-induced seismicity using time-dependent force representations. Unlike tectonic earthquakes, landquakes arise from momentum exchange during mass acceleration and deceleration, and are therefore more naturally described by force histories than moment tensors. We adopted a simplified two-force model representing the dominant acceleration and deceleration phases of a sliding mass, following terrestrial studies of large landquakes^94,95^, although realistic sources involve continuous force time series. Force-based synthetic waveforms were constructed using Green’s functions computed in the same manner as for the moment tensor inversions. Unit Green’s functions for three orthogonal force components were linearly combined to generate synthetic seismograms for arbitrary force orientations and amplitudes. The same preprocessing, filtering, time windows, and phase selections as for S0976a were applied to ensure direct comparability. Source parameters were explored using a grid search over force magnitude, orientation, and inter-force time delay, with the source depth fixed at the surface. Force amplitudes were sampled over 10^8^–10^12^ N (log-uniform), and orientations over the full sphere using a regular angular grid. Interforce time delays of 20–80 s were tested. For each time delay, independent grid searches were performed, and the best-fitting solution was selected based on minimum waveform misfit.

### Location probability based on fault density and waveform misfit

We used quake probability information as an independent prior on the spatial distribution of seismicity^50^. In this model, seismicity is assumed to occur preferentially along mapped tectonic faults, treated as first-order regional proxies for long-lived deformation zones that may be prone to stress accumulation and release, such that the spatial probability of quakes scales with local fault density. The model is provided on a regular grid with a spatial resolution of 2° × 2°. We extracted the subset of this model within the VM region and normalised the probability field to unity over the region (Supplementary Fig. 17). Independently, waveform-based constraints on source location were obtained by evaluating candidate locations defined in distance–azimuth space relative to the station. Great-circle distance was varied from 139° to 153° in increments of 1.4°, and back azimuth from 76° to 124° in increments of 6°, encompassing the previously reported location uncertainty of S0976a^38^ and the SS–PP differential-time distance estimates obtained with recent Martian interior models^43,44^. These parameters were converted to geographic coordinates to define a spatial grid. At each location, waveform inversion was performed independently to determine the best-fitting focal mechanism, and the goodness-of-fit was quantified using VR. The resulting VR values were mapped onto the corresponding spatial grid over western VM and normalised to define a relative likelihood field. Finally, we constructed a weighted location probability map by combining the two independent constraints. The normalised quake probability field was multiplied by the normalised VR field, yielding a joint probability distribution that favours locations consistent with both tectonic structure and seismic observations.

### Satellite imagery analysis for landslide detection

To investigate a landslide as the possible source for S0976a, we downloaded all available CTX (5–6 m/pix^55^) coverage of the Ius-Tithonium-Candor Chasma border region in western VM, specifically within the 0.6 probability contour and data that were acquired after August 2021 (Supplementary Fig. 20). These images covered ~51.6% of the total area (unique coverage) within the 0.6 probability contour and 53.1% of areas which cover canyon floors and walls (i.e., not the VM plateaus; unique coverage). No HiRISE coverage was downloaded as these data were entirely co-located with CTX. We used the CTX images to look for any signs of a fresh landslide, such as a new deposit (e.g., no craters, not in older CTX images) or fresh appearing scarps, by comparing them to older CTX images.

### Energy-based estimation of the minimum landslide volume

To assess whether S0976a could plausibly be generated by a landslide, we estimated the minimum landslide volume required to produce the observed seismic energy using an order-of-magnitude energy balance. The radiated seismic energy (*E*_*s*_) for an event of magnitude *M*_*w*_ = 3.6 was calculated from the empirical magnitude–energy relation^96^ log_10_ *E*_*s*_ = 1.5*M*_*w*_ + 4.8, yielding *E*_*s*_ ≈ 1.6 × 10^10^ J. Assuming this represents a fraction (*η*) of the gravitational work released by a downslope mass movement, the relationship between the source mass (*m*) and radiated seismic energy is given by

2 $${E}_{s}=\eta \,{mg\,L}(\sin \!\theta -{{\rm{\mu \!}}}\cos \!\theta )$$

where *g* is Martian gravity, *L* is runout distance, *θ* is slope angle, and µ is the effective basal friction coefficient.

We adopted *g* = 3.71 m/s^2^, *L* = 5 km, and *θ* = 35°, consistent with the geometry of large slope failures mapped in VM^26,56^. The effective friction coefficient was set to µ = 0.55, within the range inferred for dry to weakly lubricated Martian rock avalanches^97^. The seismic efficiency *η* is taken as 10^−4^ to 5 × 10^−4^, spanning typical terrestrial estimates and representing an optimistic upper bound for efficient seismic radiation^98,99^.

These assumptions yield a required source mass of approximately 10^10^–10^11^ kg. Assuming a bulk density of 2200 kg/m^3^ for fractured upper-crustal materials^100^, chosen to provide a conservative (minimum) estimate, this corresponds to a minimum landslide volume of ~10^7^–10^8^ m^3^, equivalent to a collapse scar several hundred metres to 1 km in scale. Such a feature should be detectable in post-event CTX imagery, yet none is observed within the available coverage.

### Aftershock simulation

Synthetic marsquake catalogues were generated using the Epidemic-Type Aftershock Sequence (ETAS) model^101^, in which events trigger secondary seismicity and produce clustered sequences. Simulations were conditioned on S0976a as the mainshock, with its preferred location as the reference hypocentre. Event times follow a modified Omori law^102,103^, and magnitudes follow a Gutenberg–Richter distribution^104^. Model parameters were sampled within ranges consistent with terrestrial seismicity and constraints from the MQS catalogue^75^ (see Supplementary Text 1 for details). We generated 1000 independent ETAS catalogues using Monte Carlo sampling over a 100-day window, with each catalogue capped at 2000 events to prevent rare, unrealistically large branching cascades from dominating the ensemble. Seismicity was simulated down to a magnitude of 1.0 to sustain triggering of small events. A completeness threshold of 1.5 was then applied, and only events above this level were retained. After filtering, catalogues typically contain tens to a few hundred events. Maximum aftershock magnitudes were limited following Båth’s law^105,106^.

Event locations were sampled within a 10° radius of the mainshock epicentre, with probability decreasing with distance according to a Gaussian distribution (*σ* = 3°) (Supplementary Fig. 21). Depths were uniformly sampled between 0 and 50 km, within the estimated crustal thickness in western VM^107^. Focal mechanisms were assigned by random sampling of strike, dip and rake, and converted to moment tensors scaled to match the assigned magnitudes. Synthetic seismograms were computed for all events using a consistent forward-modelling approach based on one-dimensional Martian interior models^43,44^. Waveforms were filtered to the frequency bands applied to the real data, and peak amplitudes of the vertical component were measured and compared with background noise levels to assess detectability.

## Supplementary information

Supplementary Information

Transparent Peer Review file

## Data Availability

The InSight event catalogue (https://doi.org/10.12686/a21) and waveform data are available from the IRIS-DMC (https://ds.iris.edu/ds/nodes/dmc/tools/mars-events/), the NASA Planetary Data System (PDS) (https://pds-geosciences.wustl.edu/missions/insight/seis.htm), and the IPGP data centre (https://doi.org/10.18715/SEIS.INSIGHT.XB_2016). The global CTX mosaic is available through the Caltech Murray Lab (https://murray-lab.caltech.edu/CTX/) and is archived in the PDS (https://doi.org/10.17189/m23v-ae12). Topographic data are derived from Mars Orbiter Laser Altimeter (MOLA) global datasets, available through the NASA PDS. Gravity data are derived from the MGM2011 Mars gravity model^108^, available at https://ddfe.curtin.edu.au/models/MGM2011/. The waveform data used for the single-station validation test were downloaded through the EarthScope FDSN dataselect service (https://service.earthscope.org/fdsnws/dataselect/1/), and the reference focal-mechanism solution was obtained from the Global Centroid Moment Tensor (Global CMT) Catalogue (https://www.globalcmt.org/CMTsearch). Figures were generated using the Generic Mapping Tools (GMT; https://www.generic-mapping-tools.org/). The processed waveform segments used for the moment tensor inversions, inversion input files, best-fitting source-parameter outputs for S0976a, fault-mapping data, boulder-fall site locations, and source data for the manuscript figures have been deposited in Figshare^109^ (https://doi.org/10.6084/m9.figshare.32144254).

## Code availability

Moment tensor inversions were performed using the Python package MTUQ^86,110^, available at https://github.com/mtuqorg/mtuq. The code for calculating the Kaverina source-type diagram^111,112^ used in Fig. 3f is available at https://github.com/ElsevierSoftwareX/SOFTX_2018_227.
