# Supplementary material for: Active crustal straining and fault reactivation at Valles Marineris
Source: Nat Commun. 2026 Oct 15;17:10530. doi: 10.1038/s41467-026-78193-6 (PMC13672341; doi:10.1038/s41467-026-78193-6)
Supplement: Supplementary file 1 [file 41467_2026_78193_MOESM1_ESM.pdf]

Supplementary information for

Active crustal straining and fault reactivation at Valles Marineris

Jung-Hun Song[1*], Doyeon Kim[1], Folarin Kolawole[2], Joel M. Davis[1], Rebekah Parry[1], and Ross Maguire[3]

[1] Department of Earth Science and Engineering, Imperial College London, South Kensington Campus, London, SW7 2BP, United Kingdom

[2] Department of Earth and Environmental Sciences, Columbia University, New York, NY, United States of America

[3] Department of Earth Science and Environmental Change, University of Illinois Urbana-Champaign, Urbana, IL, United States of America

Corresponding Author: Jung-Hun Song (j.song1@imperial.ac.uk)

* Department of Earth Science and Engineering, Imperial College London, South Kensington Campus, London, SW7 2BP, United Kingdom

Contents:

Supplementary Text 1

Supplementary Table 1

Supplementary Figures 1 to 22

Introduction

The supporting information includes:

- A detailed description of the synthetic marsquake catalogue generation using an Epidemic-Type Aftershock Sequence (ETAS) model, including parameter selection, calibration, and Monte Carlo implementation (Supplementary Text 1).
- Misfit-defined solution space from the grid-search moment tensor inversion, illustrating trade-offs among focal-mechanism parameters (Supplementary Fig. 1).
- Variance-reduction-weighted distributions of P- and T-axis orientations for the preferred source location and across the full source-location uncertainty region (Supplementary Figs. 2–3).

- Histograms of acceptable focal-mechanism parameters (strike, dip, rake) from the full inversion ensemble (Supplementary Fig. 4).
- Synthetic recovery tests and sensitivity analyses assessing the robustness of single-station moment tensor inversion across different source mechanisms and locations (Supplementary Figs. 5–9)
- Comparison of focal-mechanism solution spaces with and without the radial PP component as a function of centroid depth and back-azimuth (Supplementary Fig. 10).
- Validation of the single-station PP–SS body-wave inversion method using a terrestrial reference earthquake (Supplementary Fig. 11).
- Comparison of waveform fitting and focal-mechanism solutions obtained using alternative one-dimensional Martian interior models (Supplementary Fig. 12).
- Distance-dependent variations of PP and SS waveforms and their influence on waveform similarity and inversion performance (Supplementary Figs. 13–14).
- Sensitivity test examining the influence of the SKS phase in the inversion (Supplementary Fig. 15).
- Independent amplitude-based moment tensor inversion and associated waveform corrections accounting for free-surface reflections (Supplementary Fig. 16)
- Spatial constraints on source location from fault-density-derived quake probability and variance reduction fields (Supplementary Fig. 17)
- Polarisation analysis of the PP phase and probabilistic single-station source location using polarisation and travel-time constraints (Supplementary Figs. 18–19).
- Satellite image coverage used for landslide assessment in the inferred source region (Supplementary Fig. 20)
- Hypothetical aftershock distributions derived from stochastic ETAS simulations conditioned on S0976a (Supplementary Fig. 21)
- Regional structural mapping of Valles Marineris and surrounding areas, including mapped tectonic features and their relationship to the inferred source region (Supplementary Fig. 22)
- References

Supplementary Text 1. Synthetic marsquake catalogue generation

Synthetic marsquake catalogues were generated using a stochastic earthquake triggering framework based on the Epidemic-Type Aftershock Sequence (ETAS) model[1,2]. In this formulation, each seismic event can trigger its own aftershocks, resulting in cascading earthquake sequences characteristic of branching processes in seismicity[3]. The temporal decay of aftershock rates follows the modified Omori law[4,5]:

$$\lambda(\mathrm{t}) = K/(t+c)^{p} \tag{1}$$

where K controls aftershock productivity, p governs the temporal decay rate of seismicity, c is a short-time regularisation parameter, and t is time after the mainshock. All simulations were conditioned on S0976a as the initiating mainshock, with a fixed moment magnitude of 3.6 and occurrence time set to zero. Each sequence was simulated over a time window of 100 days. To prevent rare, unrealistically large branching cascades from dominating the Monte Carlo ensemble, the total number of events in a single realisation was capped at 2,000 before applying the catalogue completeness threshold. This cap was used as a numerical safeguard rather than as a physical constraint on Martian aftershock productivity. No separate cap was imposed on the number of direct secondary events generated by an individual parent event; offspring numbers were determined by the sampled ETAS productivity parameters and the modified Omori temporal distribution.

Because the physical mechanisms controlling aftershock triggering on Mars are not yet well constrained, model parameters were sampled across ranges typical of terrestrial seismicity[6]. Previous studies of global aftershock sequences[4] report Omori decay exponents typically between 0.8 and 1.5. In this study we sampled p in the range 1.0–1.3. The short-time parameter c was sampled logarithmically:

$$c = 10^{(U(-2.5,-1.5))}\ \text{days} \tag{2}$$

These values represent commonly observed ranges in terrestrial ETAS studies and serve as physically plausible proxies for Martian seismicity in the absence of direct observational constraints[7].

Although ETAS parameters are derived from terrestrial seismicity, the model provides a physically grounded statistical method for clustered event triggering and has been widely applied in environments where detailed physical constraints are limited. In the absence of sufficient Martian observational data, this approach allows exploration of a broad and physically plausible range of seismic behaviours.

- **Magnitude–frequency distribution**

Earthquake magnitudes in the synthetic catalogues were generated following the Gutenberg–Richter relationship[8]:

$$\log_{10} N(M) = a - bM \tag{3}$$

which describes the statistical relationship between earthquake magnitude (M) and frequency (N). For Mars, the magnitude–frequency distribution has been estimated from the seismic catalogue recorded by

the InSight mission. Analysis of the global seismic moment rate of Mars[9] suggests a b-value close to 1.06. To incorporate uncertainty in the Martian magnitude-frequency distribution, the b-value in our simulations was randomly sampled from the range $b \in [1.0, 1.12]$.

- **Aftershock productivity**

In the ETAS, the expected number of aftershocks triggered by an event of magnitude M is given by $K(M) = K_0 \cdot 10^{\{\alpha(M-M_c)\}}$, where K_0 controls the baseline productivity of the sequence, α describes how productivity scales with magnitude, and M_c represents the triggering magnitude threshold. Because productivity parameters for Mars remain unconstrained by observations, these parameters were sampled across ranges typical of terrestrial ETAS studies. Specifically, α was sampled from 0.6–1.0 and K_0 from $10^{(U(-3,-1))}$. These values span the range typically inferred from global earthquake catalogues and allow the synthetic simulations to represent both weakly and strongly clustered seismic sequences.

- **Maximum aftershock magnitude constraint**

To ensure physically realistic aftershock sequences, the maximum magnitude of secondary events was constrained using the empirical relationship known as Båth's law[10,11]. This relationship states that the difference between the mainshock magnitude and the largest aftershock magnitude is approximately constant. Because the applicability of Båth's law to Martian seismicity remains uncertain due to the limited number of observed large marsquakes, the magnitude difference $\Delta M = M_{main} - M_{largest}$ was treated as a stochastic variable sampled around the canonical value of approximately 1.2 magnitude units. The magnitude difference was sampled from a normal distribution centred at 1.2 with a standard deviation of 0.3 to account for natural variability.

- **Simulation magnitude threshold and catalogue completeness**

Synthetic seismicity was generated down to a minimum magnitude of 1.0 to ensure that small events capable of sustaining the ETAS triggering cascade were included. A magnitude completeness threshold of 1.5 was then applied, and only events above this level were retained in the final synthetic catalogues. This approach accounts for observational limitations of planetary seismic monitoring, while preserving smaller events required for physically consistent triggering behaviour. After applying the completeness threshold, catalogues typically contain tens to a few hundred events and rarely exceed ~150 events. Synthetic marsquake hypocentres were generated relative to the preferred location of S0976a. Event locations were sampled within a 10° radius of the mainshock epicentre, with probability decreasing with distance according to a Gaussian distribution (σ = 3°) (Supplementary Fig. 21). Event coordinates were converted to geographic latitude and longitude assuming a spherical planetary radius of 3389.5 km. Hypocentral depths were randomly sampled between 0 and 50 km, consistent with depth ranges inferred from marsquake waveform modelling. Focal mechanisms were assigned by uniform random sampling of strike (0–360°), dip (0–90°), and rake (−180° to 180°), without imposing a

preferred faulting style. Moment tensors were computed from these mechanisms and scaled to match the assigned moment magnitudes using standard seismic moment–magnitude relationships[12].

- **Monte Carlo ensemble generation**

Large ensembles of synthetic marsquake catalogues were generated through Monte Carlo sampling of all stochastic parameters described above. Independent catalogues were generated using different random seeds to ensure statistical independence between realisations. Each catalogue contains the event occurrence time relative to the initiating event, moment magnitude, geographic location, source depth, and full moment tensor representation. This ensemble-based analysis enables robust statistical exploration of plausible marsquake scenarios consistent with current observational constraints.

Supplementary Table 1. Statistics of the acceptable focal-mechanism ensemble.

Quantity	Value
Total models explored	~8.72 $\times 10^9$
Acceptable solutions (VR > 0.9 × VRmax)	164,877
Strike-slip family (SS + SS-R + SS-N)	82.2 %
Strike cluster 1	48.0° ± 21.5°
Strike cluster 2	143.5° ± 22.5°
Dip (median)	79.0°
Dip (16th–84th percentile range)	62.5°–86.5°
Rake cluster 1	8.0° ± 26.0°
Rake cluster 2	174.0° ± 24.5°

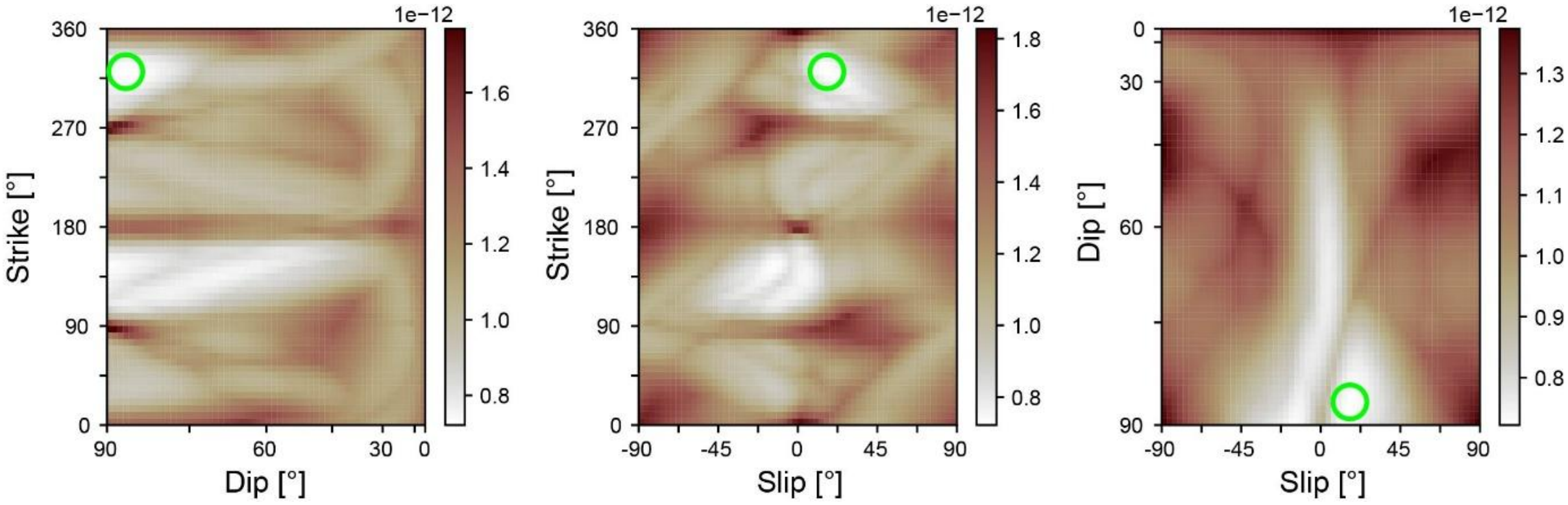

Supplementary Figure 1: Misfit-defined solution space from the grid-search moment tensor inversion, illustrating trade-offs between source parameters. Panels show pairwise relationships between (left) dip and strike, (middle) rake and strike, and (right) rake and dip. Colours indicate the normalised L_2 waveform misfit. The green circle marks the best-fitting solution. The misfit minimum is well confined, favouring a near-vertical, strike-slip faulting mechanism. The misfit corresponds to the L_2 norm of waveform residuals, defined as the summed squared differences between observed and synthetic waveforms[13].

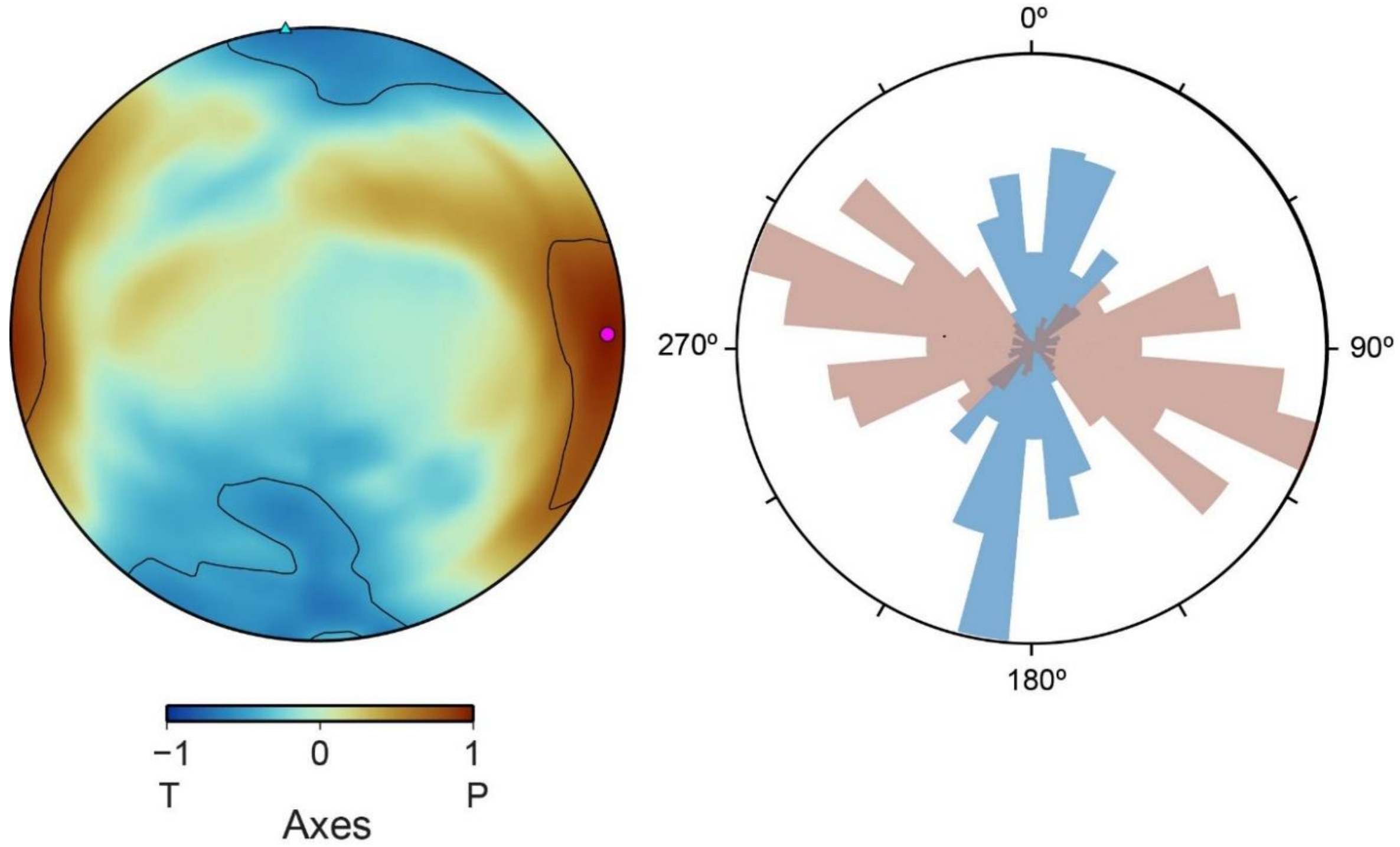

Supplementary Figure 2: Variance-reduction (VR)-weighted distribution of P- and T-axis orientations for S0976a. (Left) Stereographic projection of VR-weighted P- (compressional) and T- (tensional) axis orientations derived from the full ensemble of moment tensor solutions at the preferred epicentre. The ensemble spans source depths from 1 to 50 km (1 km increments) and the full grid of strike, dip and rake parameters explored in the inversion (see "Methods"). Each solution is weighted by its VR, and the resulting density field is normalised to emphasise the most strongly supported orientations. Warmer colours denote the P-axis distribution, whereas cooler colours denote the T-axis distribution. Density maxima are marked by a magenta circle (P axis) and a cyan triangle (T axis). Black contours indicate the angular region corresponding to the one-standard-deviation (1σ) spread around each maximum. (Right) Rose diagram showing the distribution of P- (brown) and T- (blue) axis orientations from the ensemble.

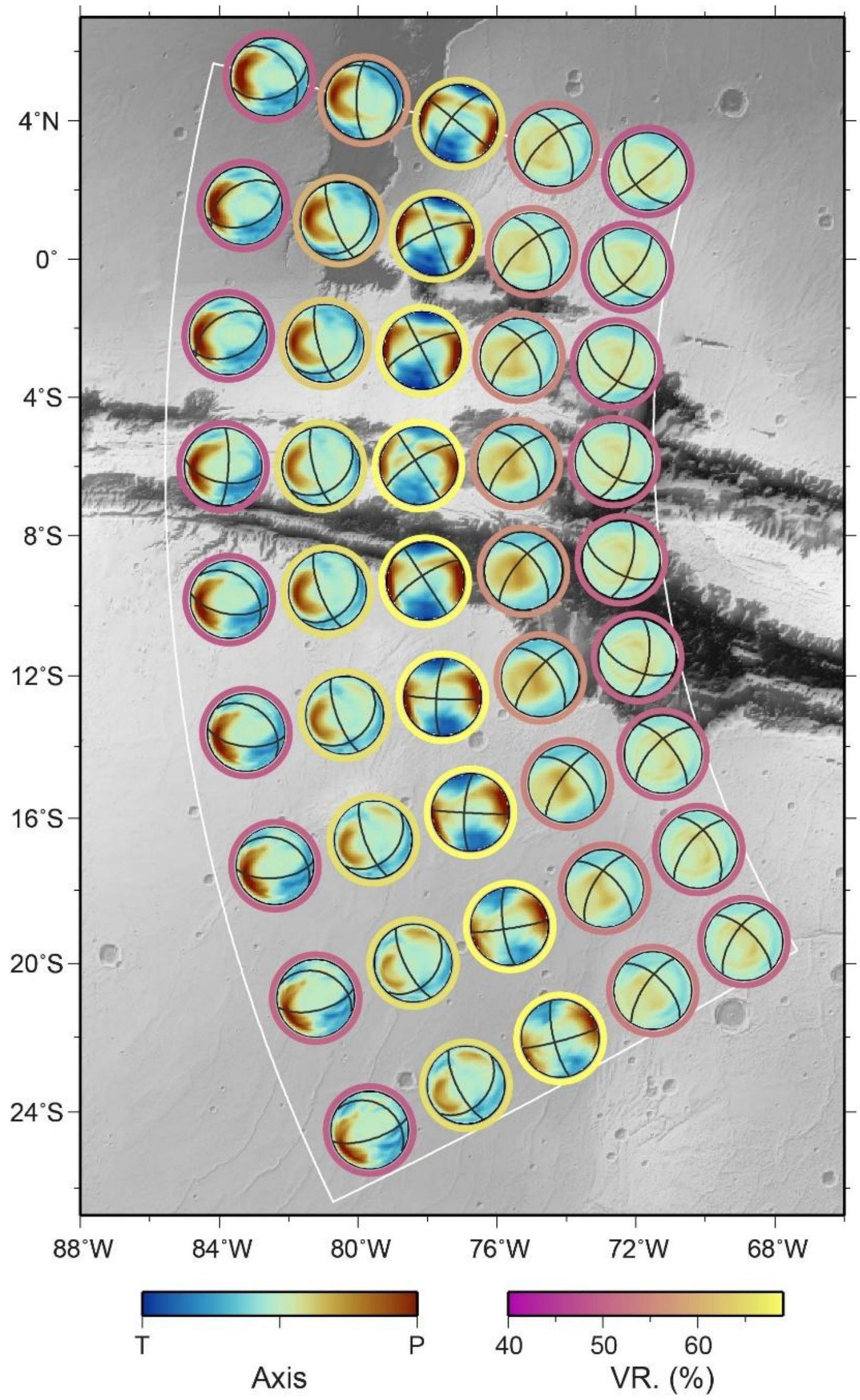

Supplementary Figure 3: VR-weighted distribution of P- and T-axis orientations for S0976a across candidate source locations**.** Stereographic projection showing the VR-weighted distribution of P- (compressional) and T- (tensional) axis orientations derived from the ensemble of moment tensor solutions across candidate source epicentres within the location uncertainty region[14]. For each epicentral location, the full set of trial solutions spanning depths from 1 to 50 km and the complete grid of strike, dip, and rake parameters is included. Fault planes corresponding to the best-fitting solution at each epicentral location are shown as black lines. Rim colours indicate the VR of the best-fitting solution at each location.

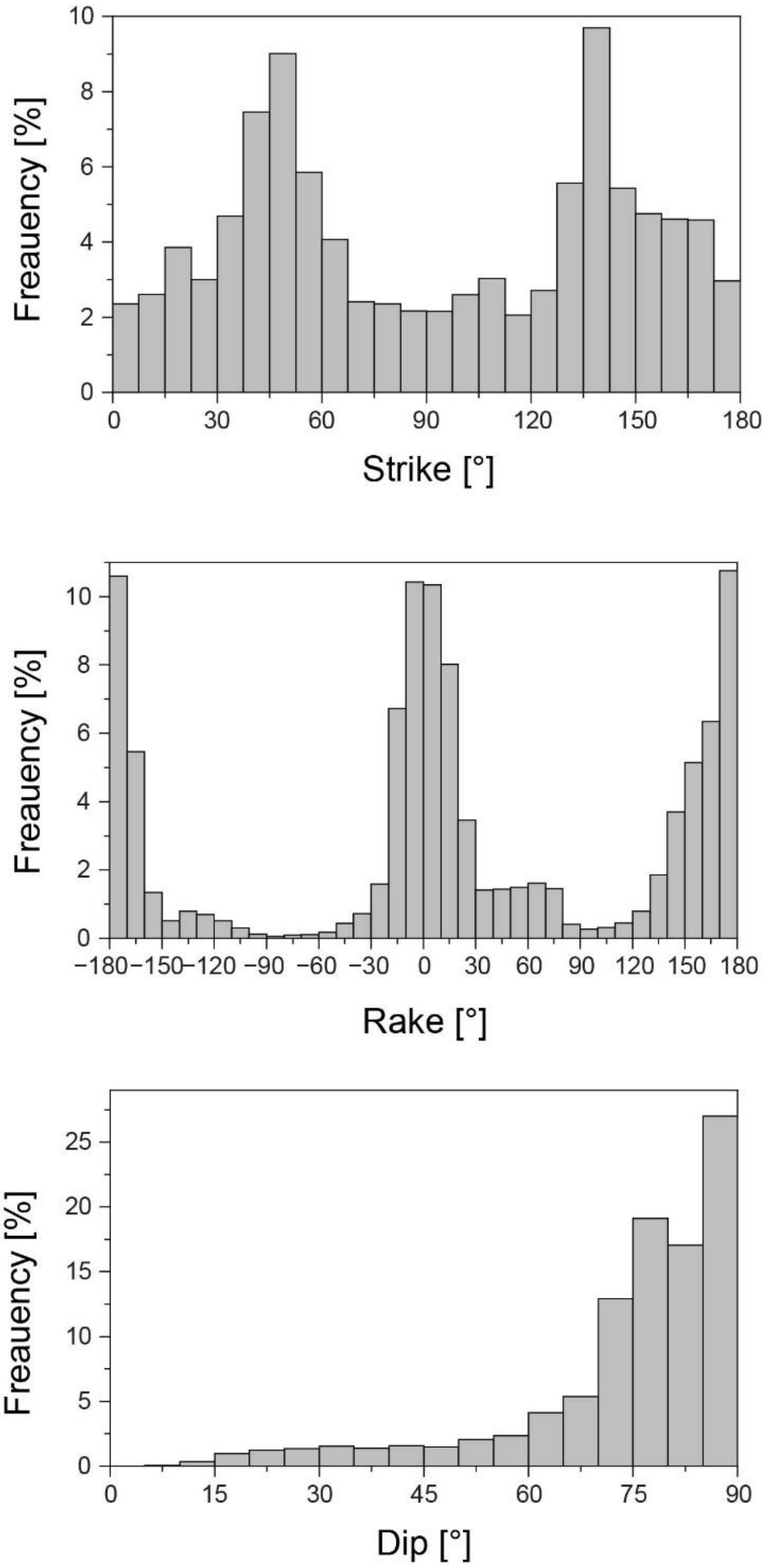

Supplementary Figure 4: Histograms showing the distributions of strike, dip, and rake for acceptable focal-mechanism solutions. The ensemble was extracted from the full grid search over strike, dip, rake, source depth, and epicentral location (see "Methods" for the complete parameter ranges explored). Summary statistics for the strike, dip, and rake distributions are provided in Supplementary Table 1. Strike orientations are folded into the 0–180° range for statistical summary.

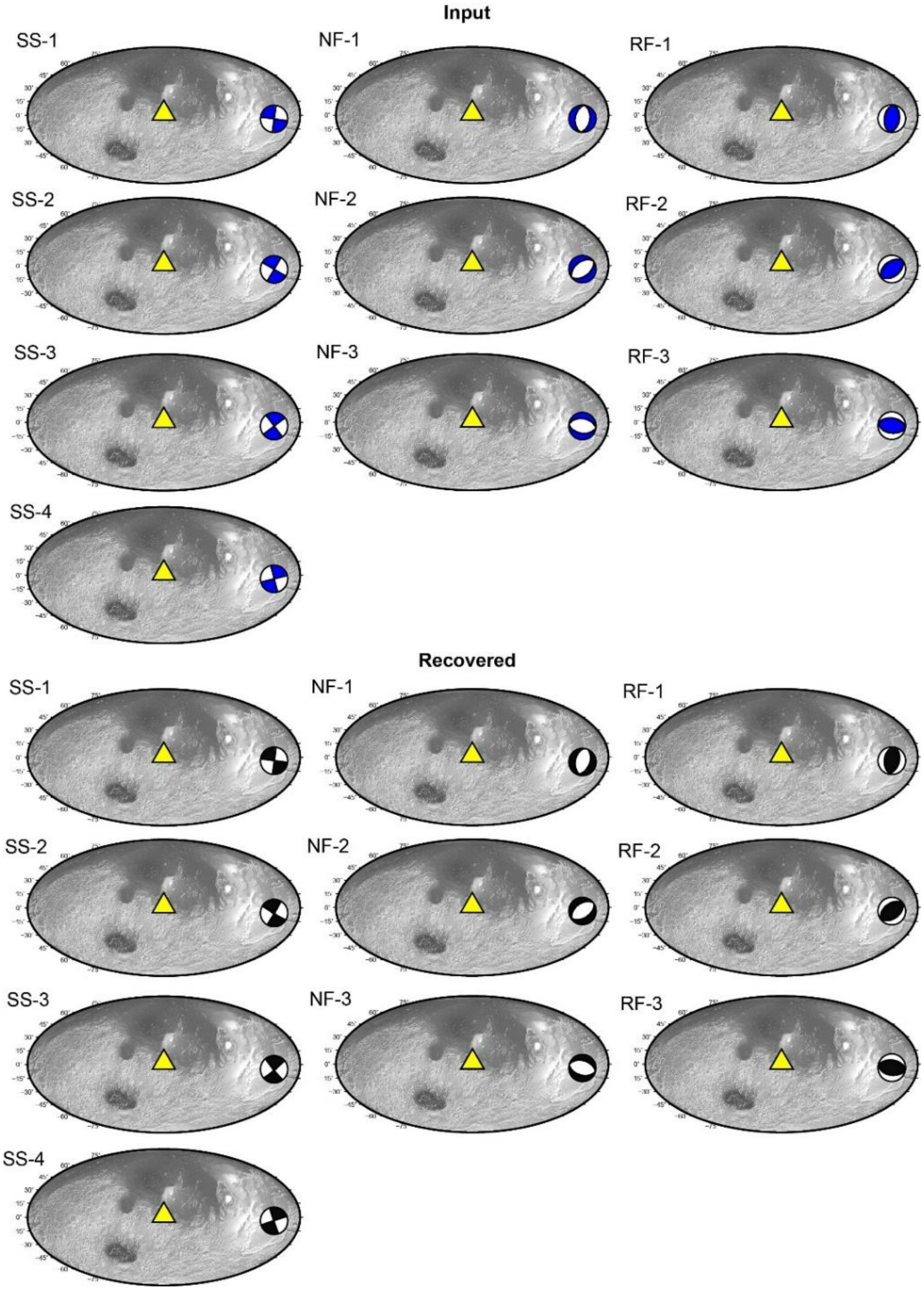

Supplementary Figure 5: Synthetic recovery tests for single-station moment tensor inversion. Synthetic focal mechanisms (top; Input) were used to generate waveforms at the S0976a source location, assuming a fixed depth of 40 km. The same single-station inversion method applied to the real data, using only PP and SS phases, was then used to recover the source mechanisms (bottom; Recovered). Strike-slip (SS), normal fault (NF), and reverse fault (RF) scenarios were tested. Strike-slip cases (SS-1 to SS-4) share identical dip (90°) and rake (180°) but vary in strike from 0° to 67.5° in 22.5° increments. Normal fault models (NF-1 to NF-3) have a fixed dip of 45° and rake of −90°, with strike varying from 0° to 90°. Reverse fault models (RF-1 to RF-3) similarly adopt a dip of 45° and rake of 90°, with strike varying from 0° to 90°. Yellow triangles indicate the station location, and beachballs in blue and black denote the input and recovered focal mechanisms, respectively.

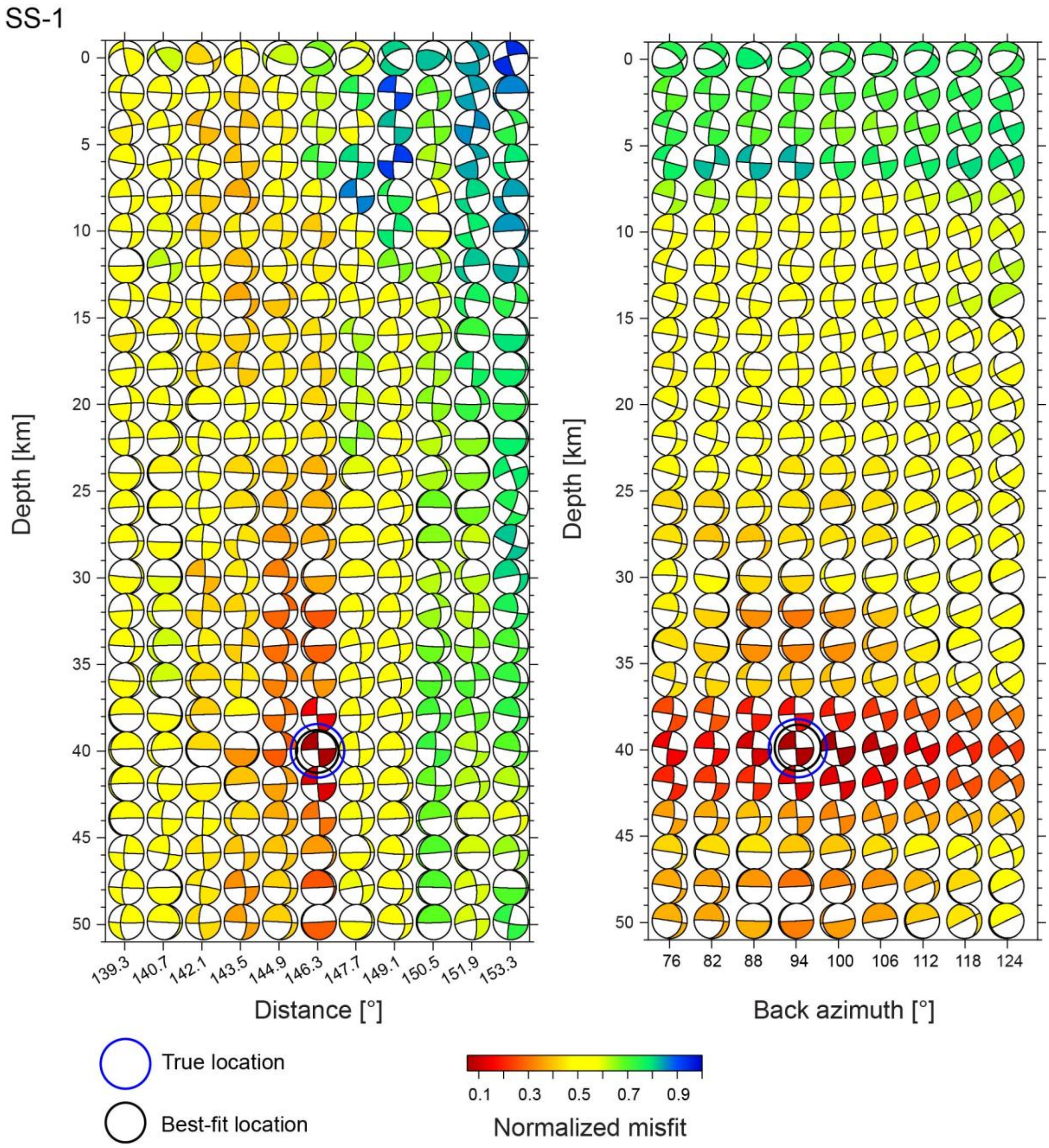

Supplementary Figure 6: Sensitivity of single-station inversion to source location for a strike-slip mechanism (SS-1). Moment tensor inversions were performed for the SS-1 synthetic focal mechanism over a range of source locations within the S0976a uncertainty region[14] and depths from 0 to 50 km (2 km increments). The left panel shows inversion results as a function of epicentral distance and depth, while the right panel shows results as a function of back azimuth and depth, with distance fixed at 146°. Beachball colours indicate the normalised misfit (colour scale), with lower misfit shown in red and higher misfit in blue. The blue circle marks the true source location used to generate the synthetic data, and the black circle indicates the best-fitting solution. The results demonstrate that while the inversion recovers the correct depth and focal mechanism near the true location, comparable solutions can be obtained across a range of azimuth–distance combinations, reflecting trade-offs inherent to single-station geometry.

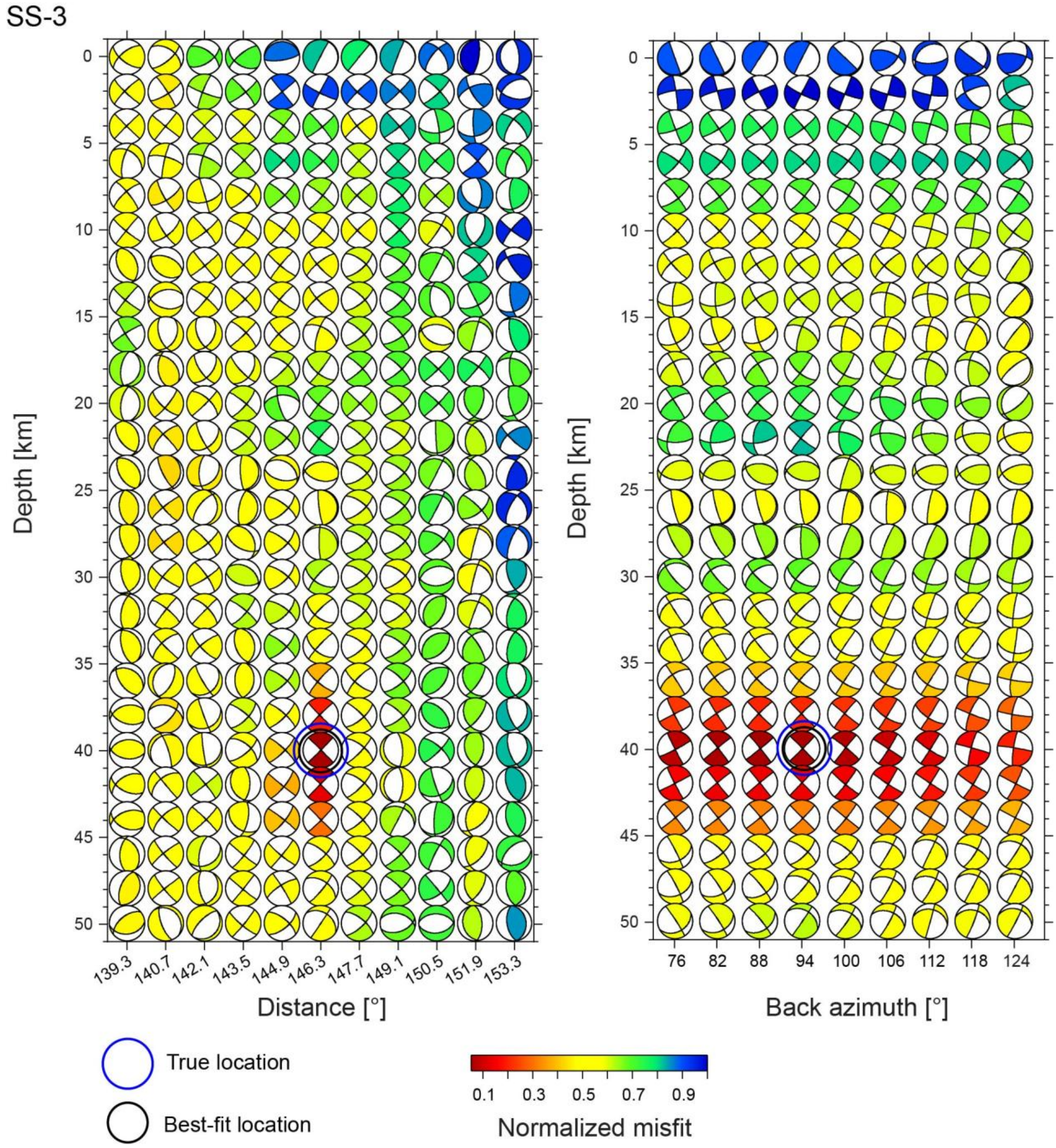

Supplementary Figure 7: Same as Supplementary Fig. 6, but for the case of the SS-3 input source mechanism.

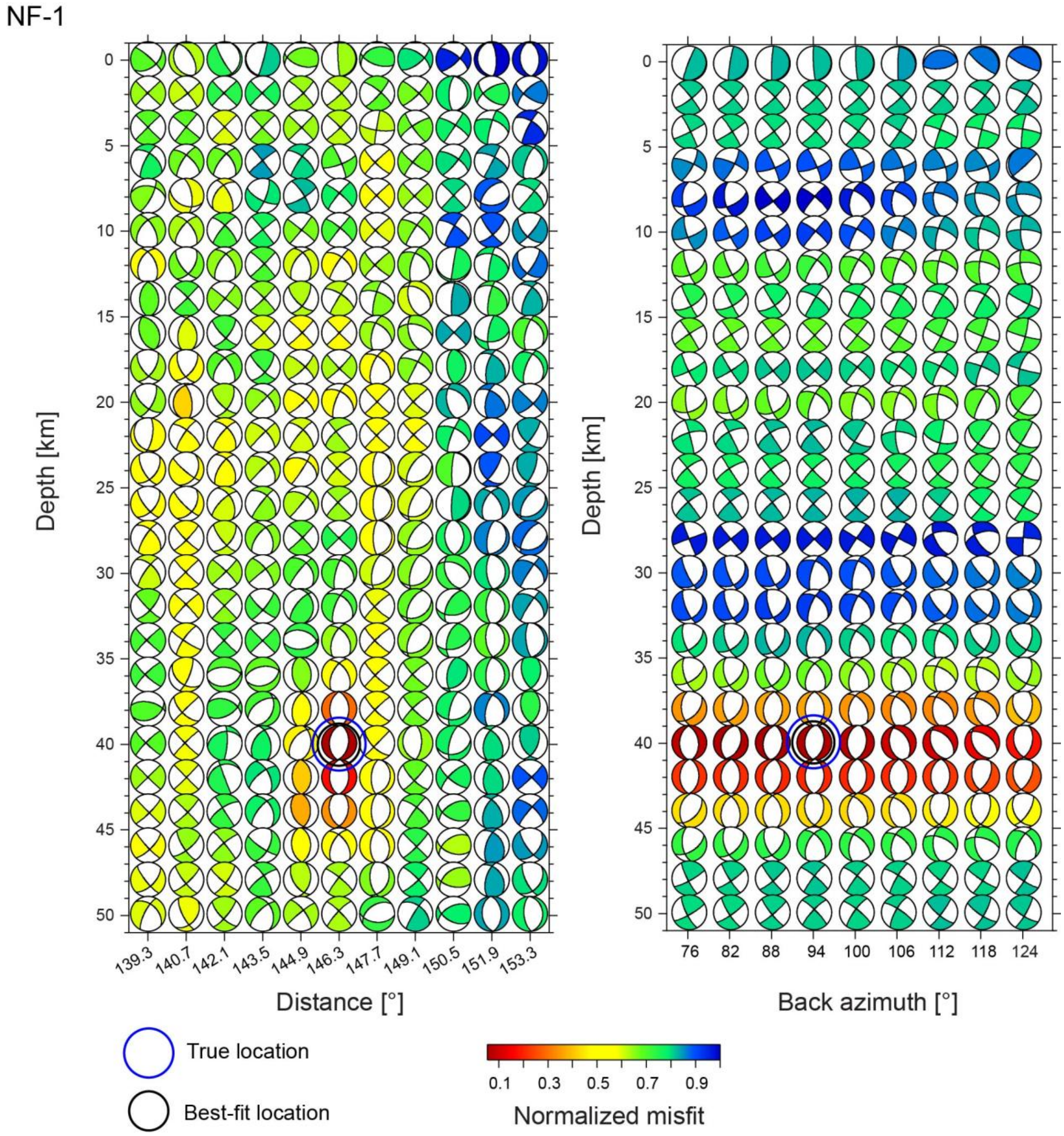

Supplementary Figure 8: Same as Supplementary Fig. 6, but for the case of the NF-1 input source mechanism.

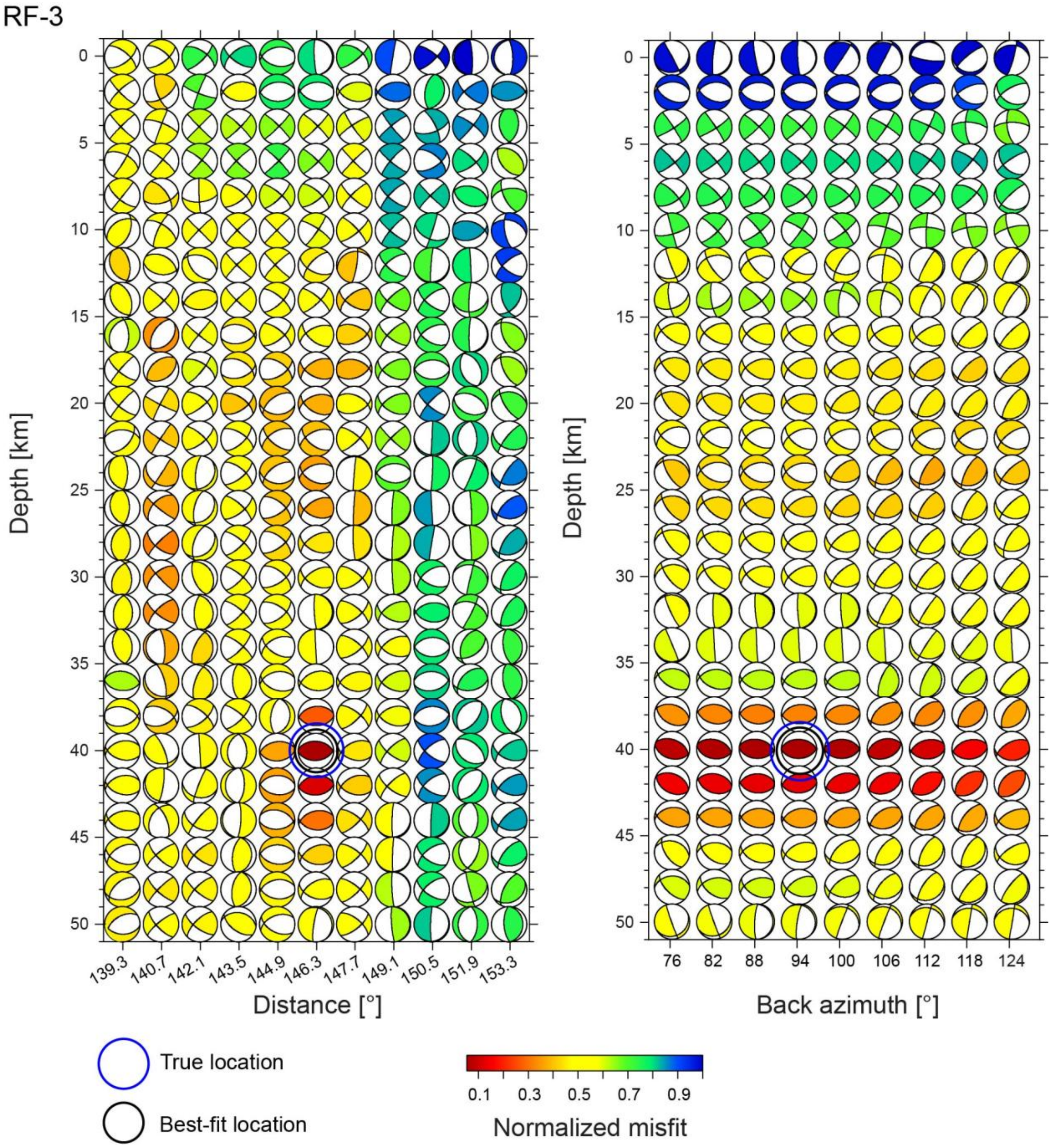

Supplementary Figure 9: Same as Supplementary Fig. 6, but for the case of the RF-3 input source mechanism.

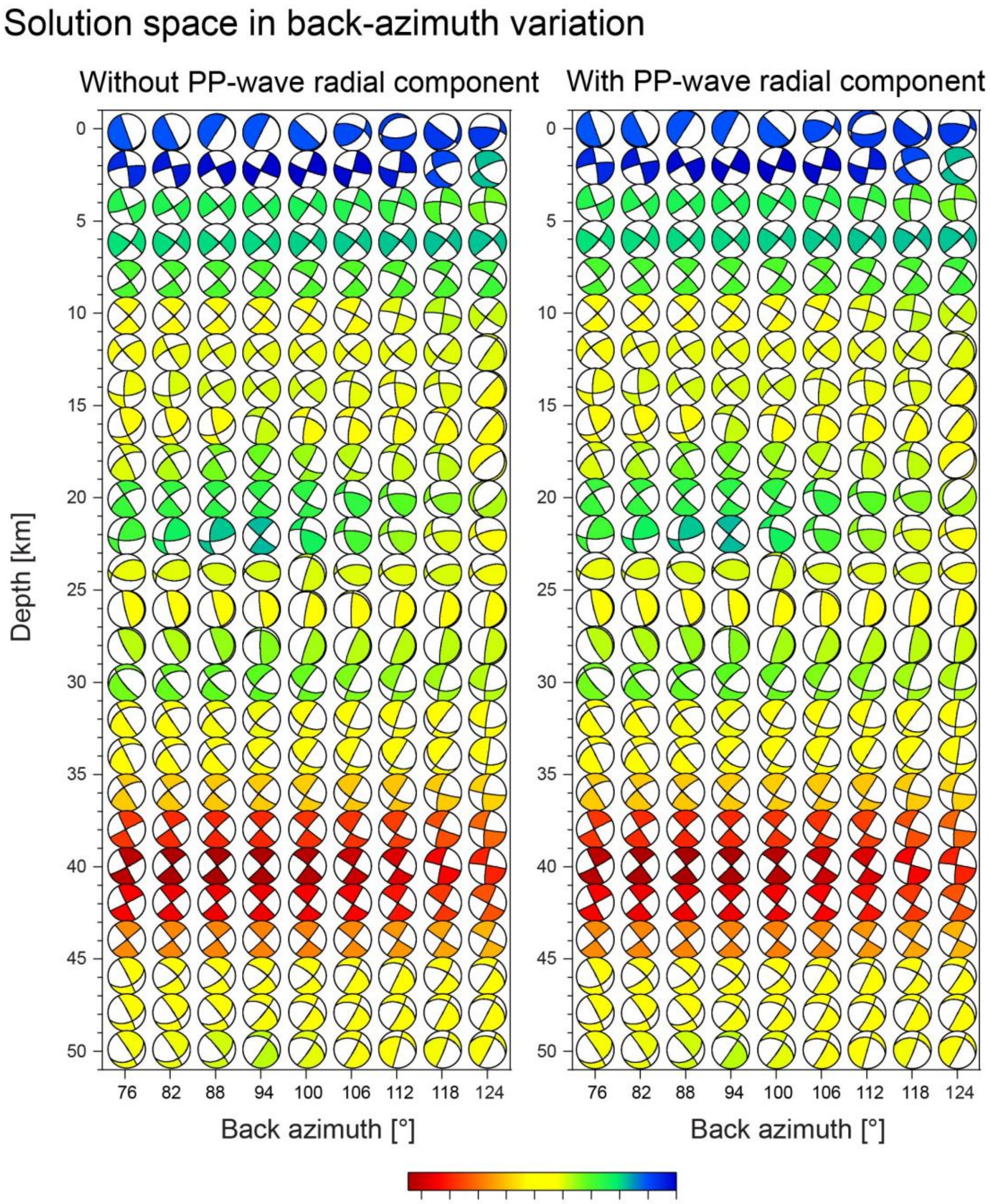

Supplementary Figure 10: Focal mechanism solution space comparison with and without inclusion of the radial component of the PP wave. Focal mechanisms are determined based on synthetic seismograms generated with input focal mechanism of SS-3 in Supplementary Fig. 7 with centroid depth of 40 km. Solutions are shown as a function of centroid depth and back-azimuth, with epicentral distance held fixed. Beachball colours indicate normalised waveform misfit. Including the radial component results in only minor changes in the misfit distribution and does not significantly alter the preferred solution. The overall solution space remains broadly similar, reflecting the limited additional constraint provided by the radial PP waveform in a single-station inversion, where trade-offs with back-azimuth persist.

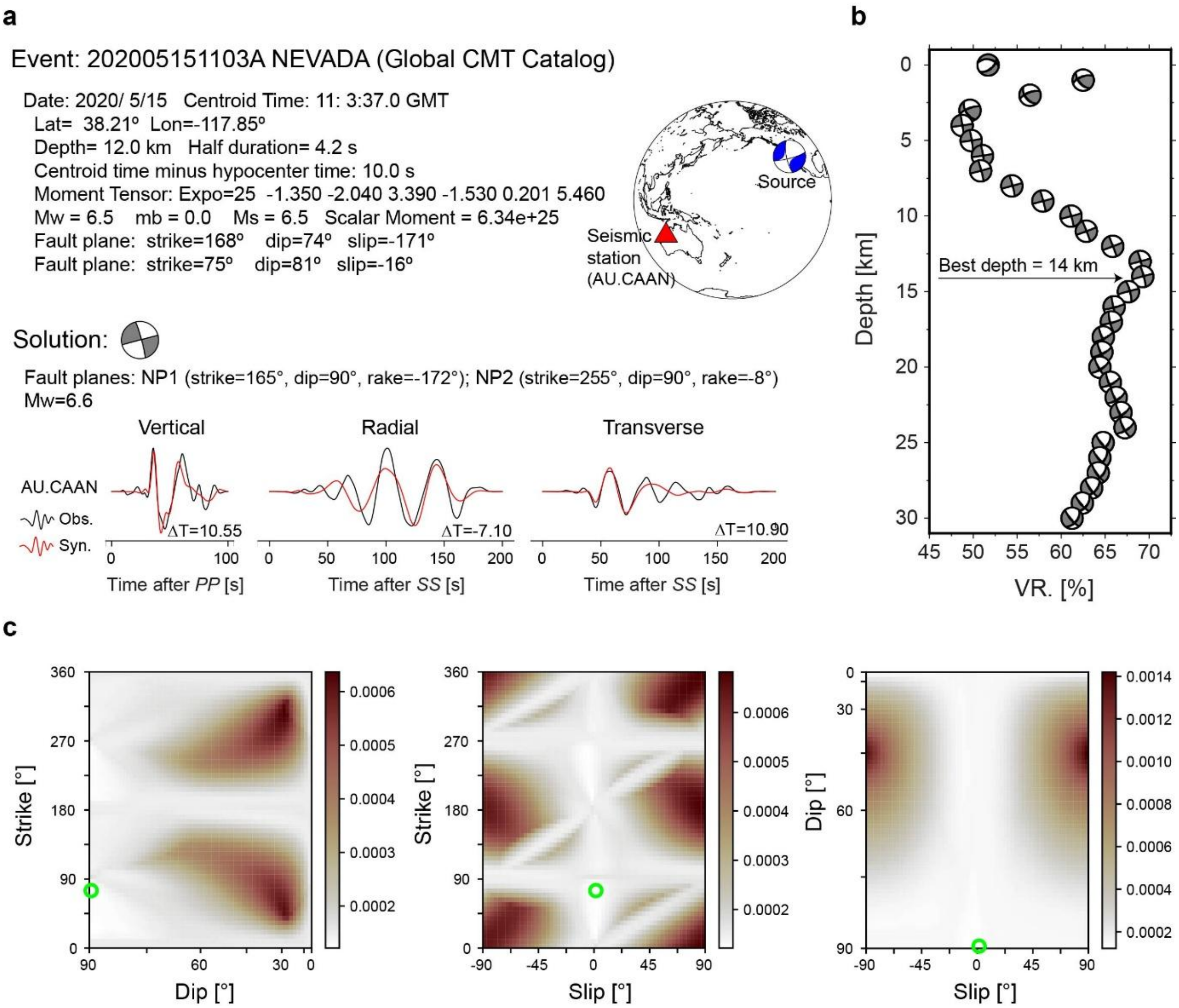

Supplementary Figure 11: Results of body-wave inversion (PP and SS phases) for a terrestrial reference event. Tests on a terrestrial earthquake (202005151103A NEVADA) recorded at a single station demonstrate that inversion of PP and SS phases recovers known source parameters comparably to direct P and S phases, supporting the application of this approach to S0976a. **a**, Top left: Source parameters from the Global CMT catalogue[15]. Top right: Geographic configuration of the event and the seismic station (AU.CAAN). Bottom: Best-fitting solution at a depth of 14 km, including focal mechanism and source parameters (strike, dip, rake, and Mw). Three-component waveform fits are shown for vertical (PP) and radial/transverse (SS) components. Observed waveforms are shown in black and synthetic waveforms in red, filtered in the 10–50 s period band. **b**, Depth-dependent inversion results showing focal mechanisms and corresponding VR. The best-fitting solution is obtained at a depth of 14 km, with a maximum VR of 69.3%. **c,** Misfit-defined solution space from the grid-search moment tensor inversion, illustrating trade-offs between source parameters. Panels show pairwise relationships between (left) dip and strike, (middle) rake and strike, and (right) rake and dip. Colours indicate the normalised L_2 waveform misfit. The green circle marks the best-fitting solution (misfit ~ 0.00012). The misfit minimum favours a near-vertical, strike-slip faulting mechanism.

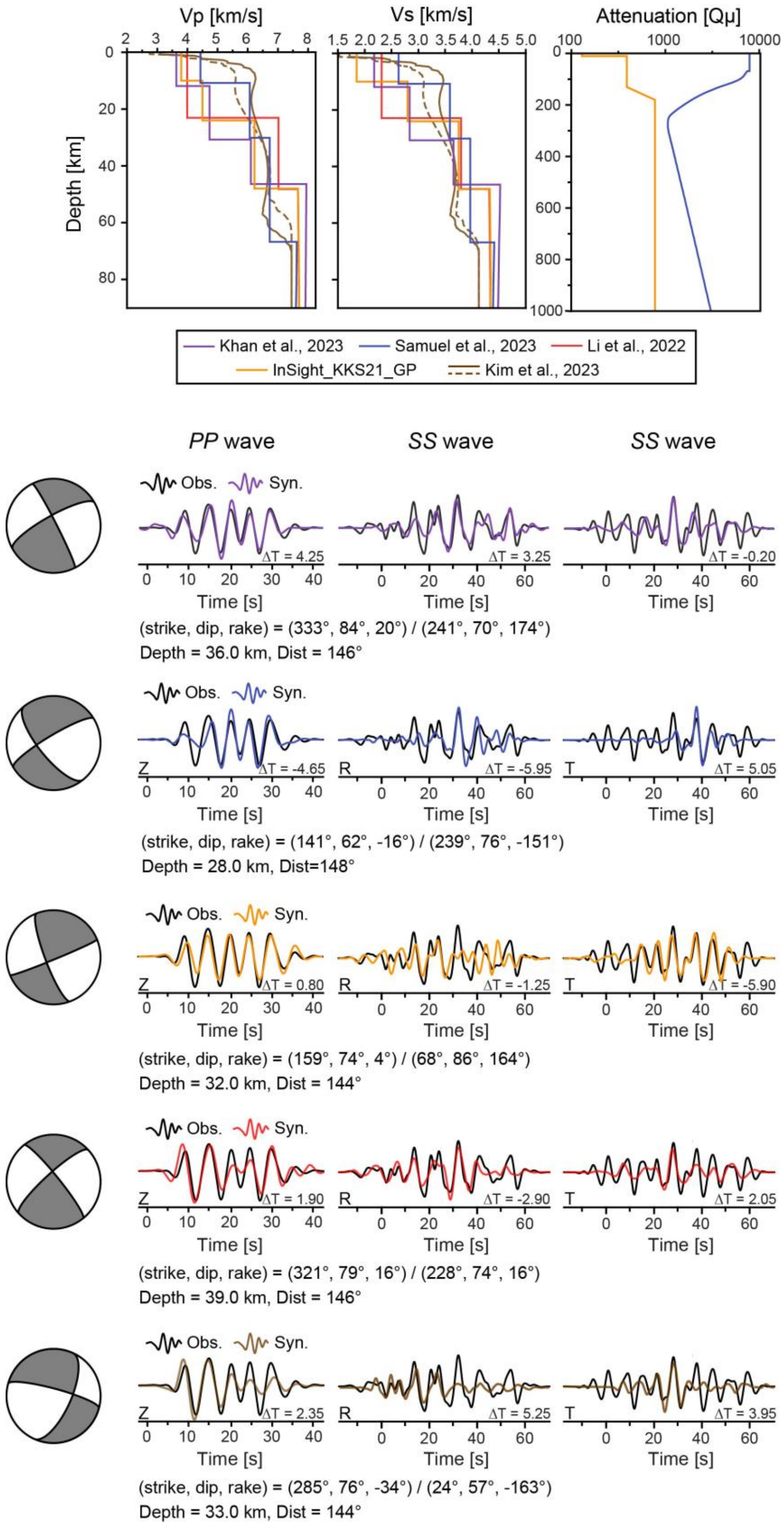

Supplementary Figure 12: Focal mechanism solution and waveform fitting using different Martian interior models. Comparison between observed and synthetic waveforms computed using different 1-D Martian velocity models (top): Khan et al.[16], Samuel et al.[17], the InSight KKS21_GP model (a combined model based on early InSight mission data[18,19,20]), Li et al.[21], and Kim et al.[22]. PP phases are shown on

the vertical (Z) component, whereas SS phases are compared on the radial (R) and transverse (T) components. Δt indicates the optimal time shift. For each velocity model, focal-mechanism solutions were searched over source distance and depth while fixing the back azimuth to the preferred value (~93°). The corresponding best-fitting source depth is indicated. For the shear quality factor (Qs) in the Khan et al. model, we adopt the parameterisation of the Samuel et al model. As Li et al. constrain only the crustal structure, their crustal model was combined with the deeper mantle structure of the Samuel et al. model. For the Kim et al. model, solid and dashed curves denote horizontally and vertically polarised P- and S-wave velocities, respectively; below ~90 km depth, the deeper mantle structure of Samuel et al. was used.

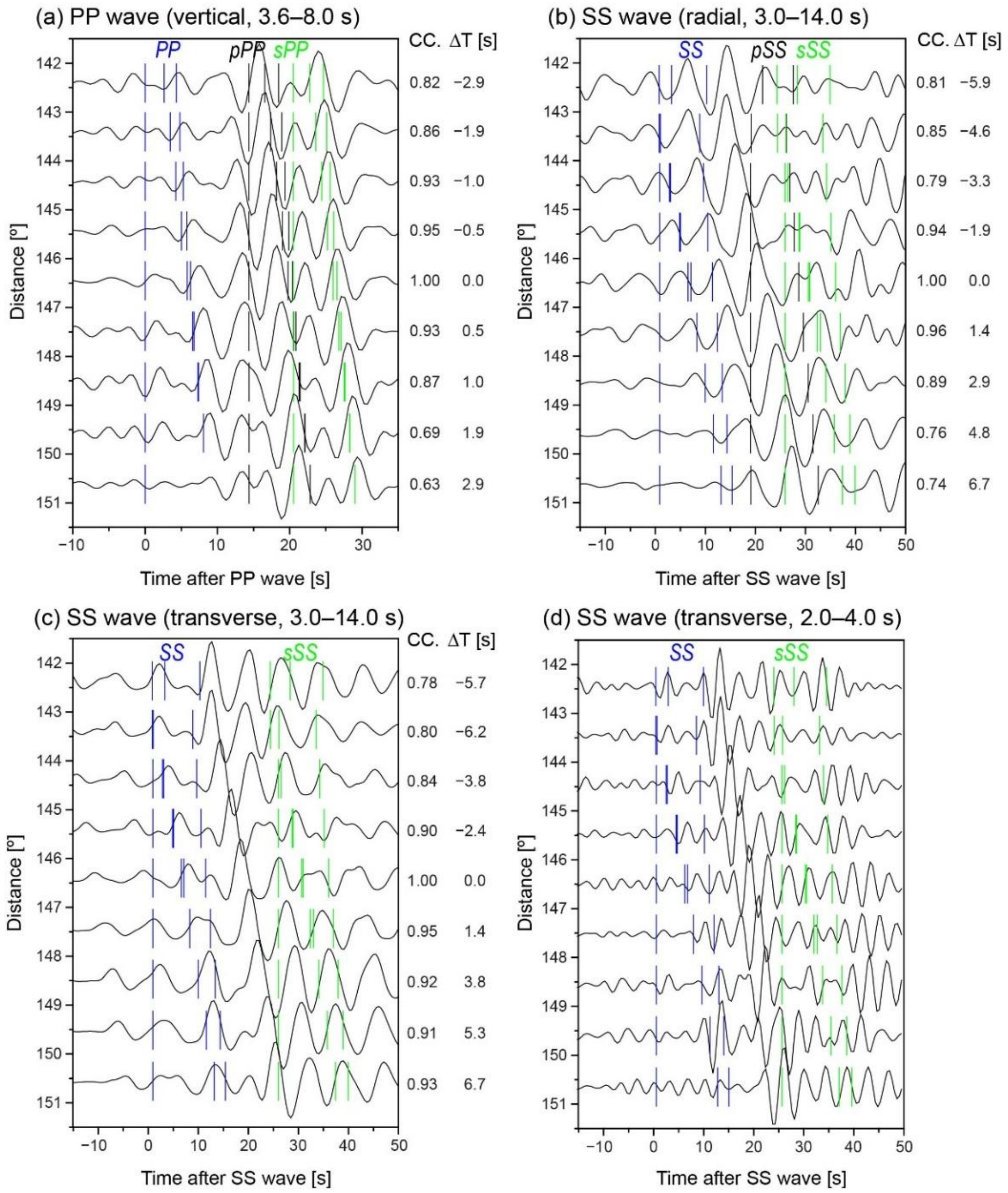

Supplementary Figure 13: Distance-dependent waveform variations of PP and SS phases. Synthetic seismograms are computed using the preferred focal mechanism solution (Fig. 2) at a fixed depth of 35 km and the interior velocity model of Khan et al.[16]. Waveforms are aligned on the predicted first arrivals of the PP or SS phases based on this velocity model. Vertical ticks indicate the theoretical arrival times of PP and SS phases (blue), as well as depth phases (pPP and pSS in grey; sPP and sSS in green). Multiple arrivals of PP, SS, or depth phases are present within a single trace; these are all predicted using the TauP travel-time toolkit[23], and all possible arrivals are displayed. To evaluate waveform stability with respect to epicentral distance, cross-correlation coefficients (CC) are calculated for each wavetrain relative to a reference waveform at 146.5°. The corresponding CC values and optimal time shifts are indicated on the right side of each panel. Apparent phase shifts and waveform distortions with increasing distance demonstrate that distance variations introduce effects beyond a simple uniform time shift.

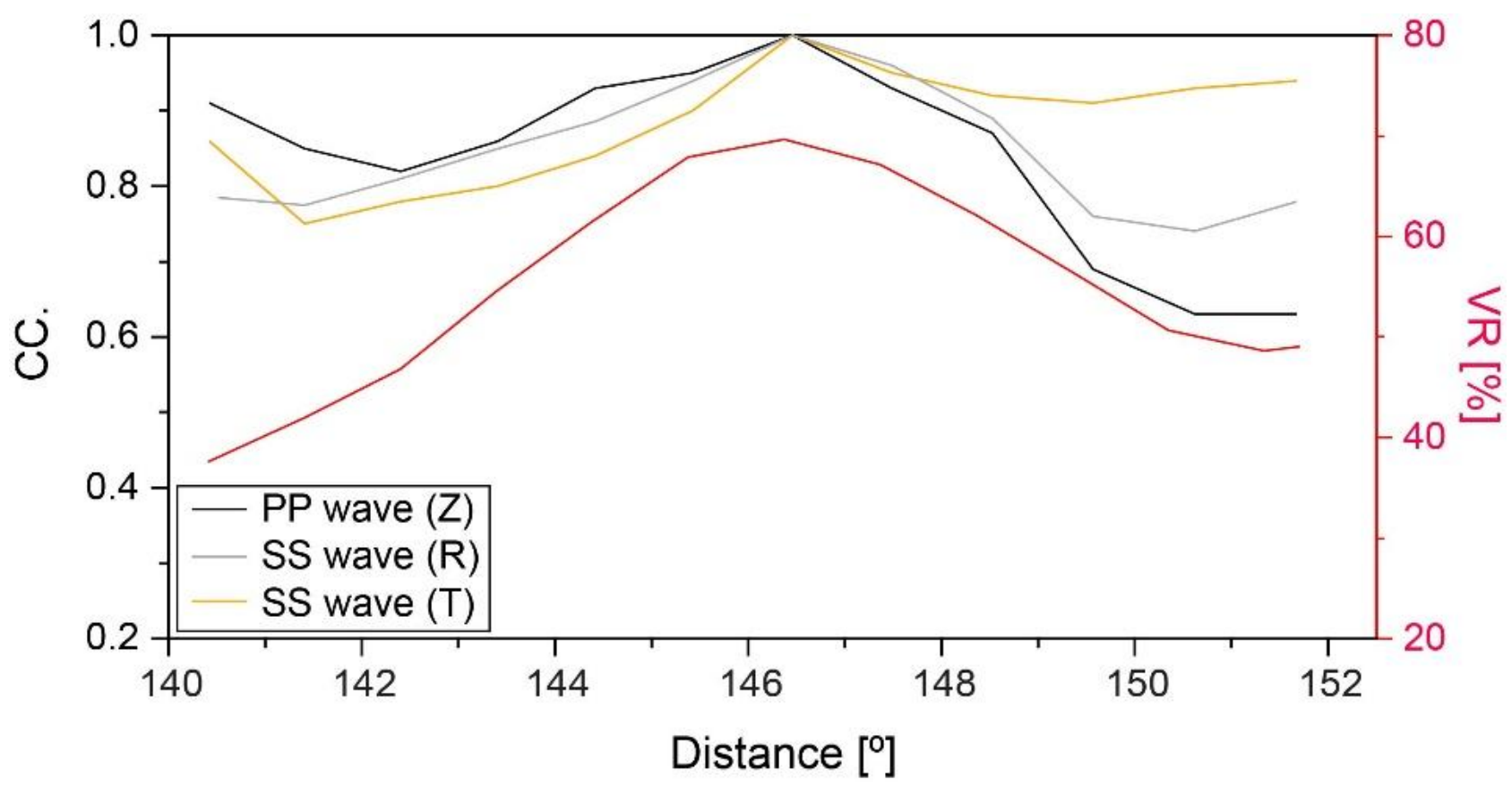

Supplementary Figure 14: Distance dependence of waveform similarity and waveform VR. CC values from the synthetic waveforms in Supplementary Fig. 13 and VR values from moment tensor inversion of the observed data (Supplementary Fig. 17) are shown as a function of epicentral distance.

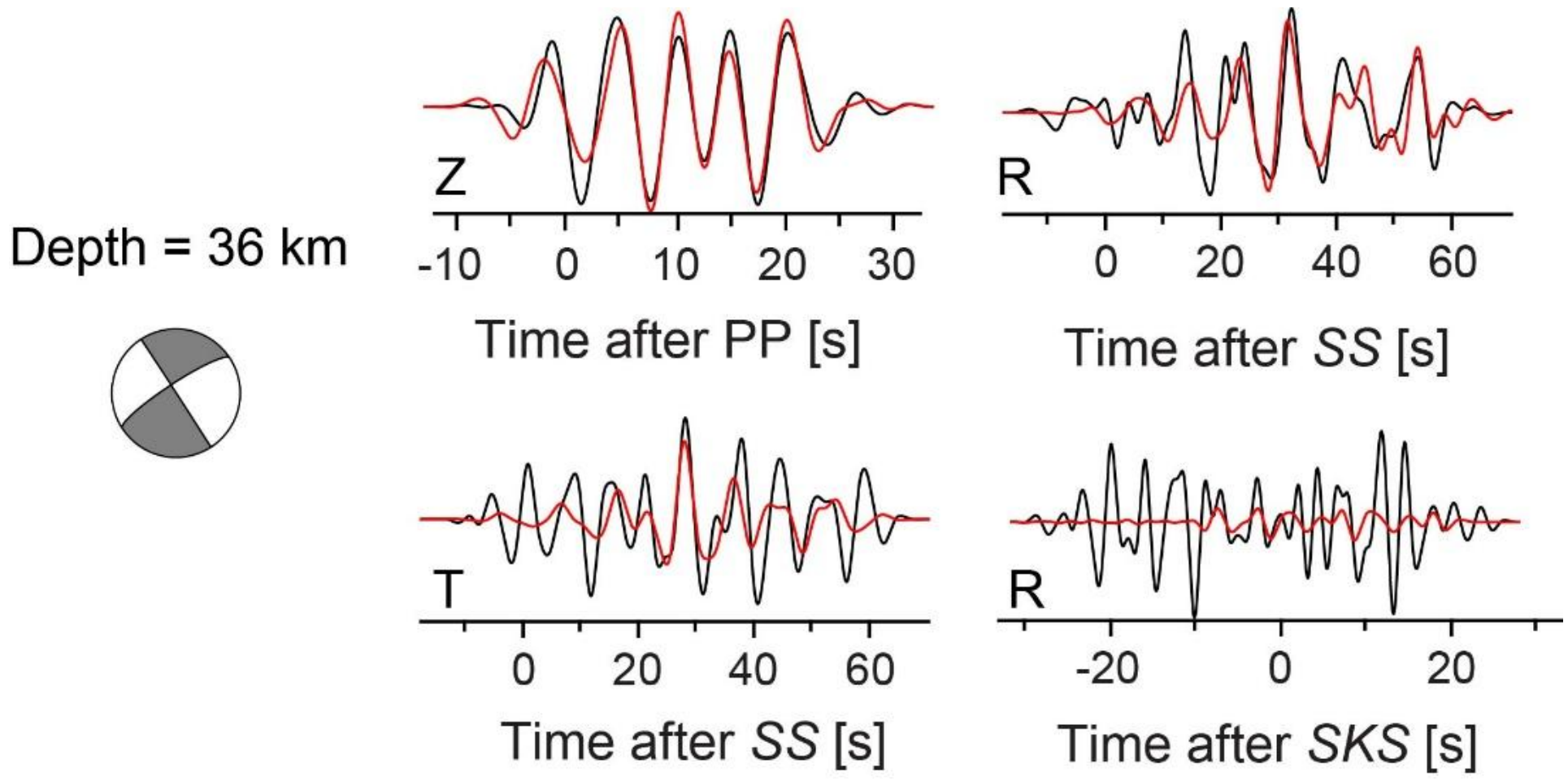

Supplementary Figure 15: The PP and SS waveforms were processed using the same frequency bands and time windows as in Fig. 2. For the SKS phase, the radial component was filtered in the 2–5 s period band and windowed around the arrival identified by frequency-dependent polarisation analysis (FDPA[24]). Observed and synthetic waveforms are shown in black and red, respectively. Inclusion of the SKS window yields a focal mechanism consistent with the preferred solution obtained without SKS, but does not improve the waveform fit or further constrain the source mechanism. This likely reflects the weak and ambiguous nature of the SKS arrival in the waveform records, which was identified primarily from polarisation analysis rather than as a distinct body-wave arrival. We therefore did not include SKS in the preferred inversion because it provides little additional constraint at the available signal-to-noise level and may introduce modelling bias from deep-mantle and core–mantle-boundary propagation not captured by the 1-D Green's functions.

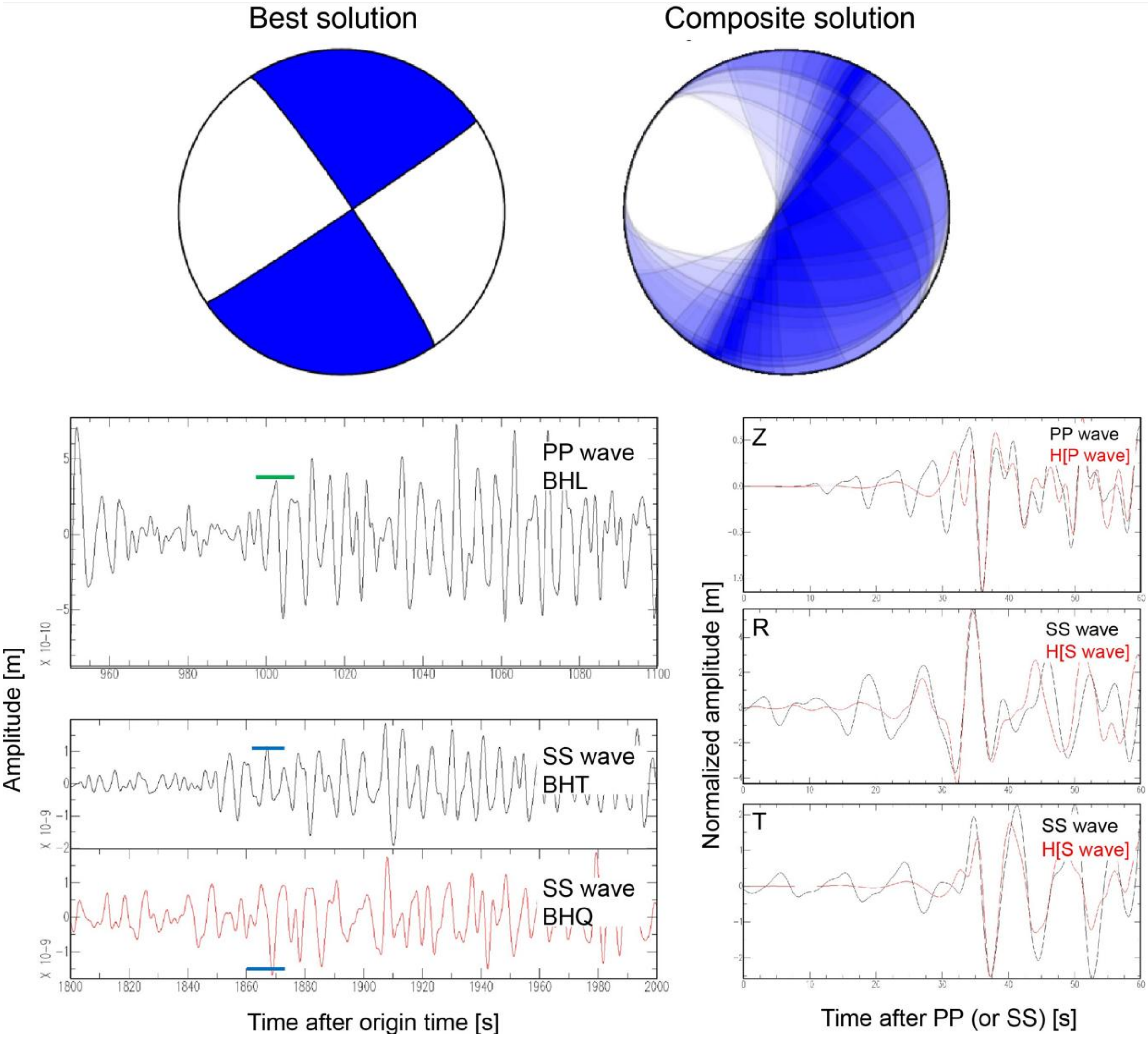

Supplementary Figure 16: Amplitude-based moment tensor inversion for S0976a. Amplitude-only moment tensor solutions following the method of Sita and van der Lee[25]. (Top) Best-fitting solution and ensemble of acceptable solutions. (Bottom left) Peak amplitudes of PP and SS phases are measured from displacement seismograms after rotation from NEZ to LQT components based on ray geometry. PP amplitudes are shown as green bars, and SS amplitudes on the transverse and radial components as blue bars. (Bottom right) Comparison between synthetic PP and SS waveforms at the full source–receiver distance (black) and Hilbert-transformed, amplitude-scaled direct P and S waveforms at half the distance (red). Prior to inversion, observed PP and SS amplitudes are rescaled using correction factors derived from this conversion to account for free-surface reflection effects.

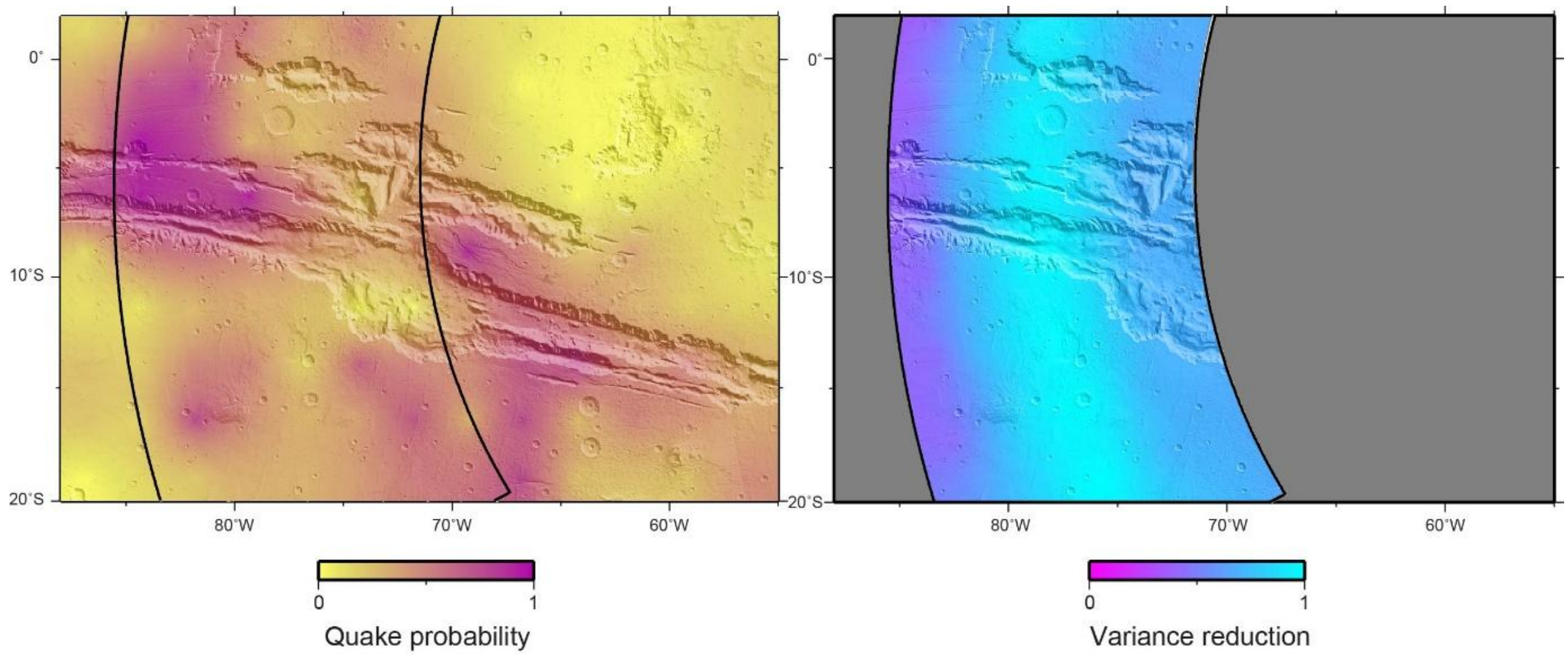

Supplementary Figure 17: Quake probability and variance reduction constraints on source location in Valles Marineris. Left: normalised quake probability map derived from fault density within Valles Marineris, where seismicity is assumed to scale with the spatial distribution of mapped tectonic structures[26]. The probability field is extracted from the global quake probability model and rescaled to unity over the study region. Right: normalised variance reduction field obtained from focal mechanism moment tensor inversions performed over a grid of candidate source locations defined in distance–azimuth space and projected onto geographic coordinates. Higher variance reduction values indicate better waveform fit. Black curves denote great-circle paths corresponding to the explored source–receiver geometry. Together, these fields provide independent constraints on source location that are combined to construct the joint location probability distribution shown in Fig. 2.

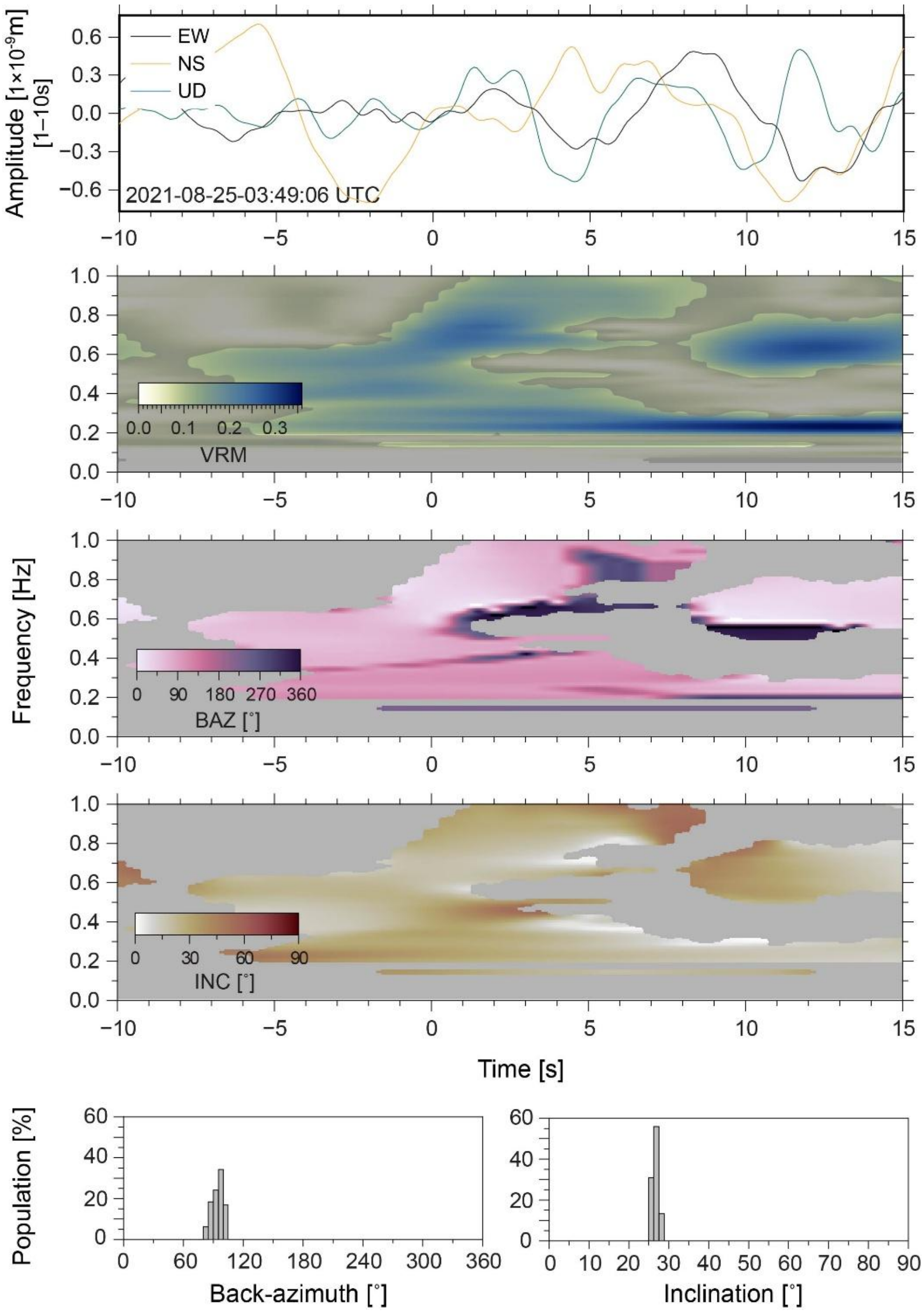

Supplementary Figure 18: Polarisation analysis of the PP wave from marsquake S0976a using three-component seismic data. From top to the fourth panel, the E–W, N–S, and U–D component seismograms and vertically rectilinear motion (VRM) are shown. The subsequent panels display back-azimuth (BAZ) and inclination (INC) estimated in the time–frequency domain. VRM was computed in the time–frequency domain, and its mean value was used as a threshold to mask (grey shaded) intervals of low rectilinearity. Back-azimuth and inclination were subsequently estimated only within high-rectilinearity regions exceeding this threshold. The BAZ and INC values shown in the lower panel were extracted from time–frequency windows spanning −3 to +5 s relative to the P-wave arrival.

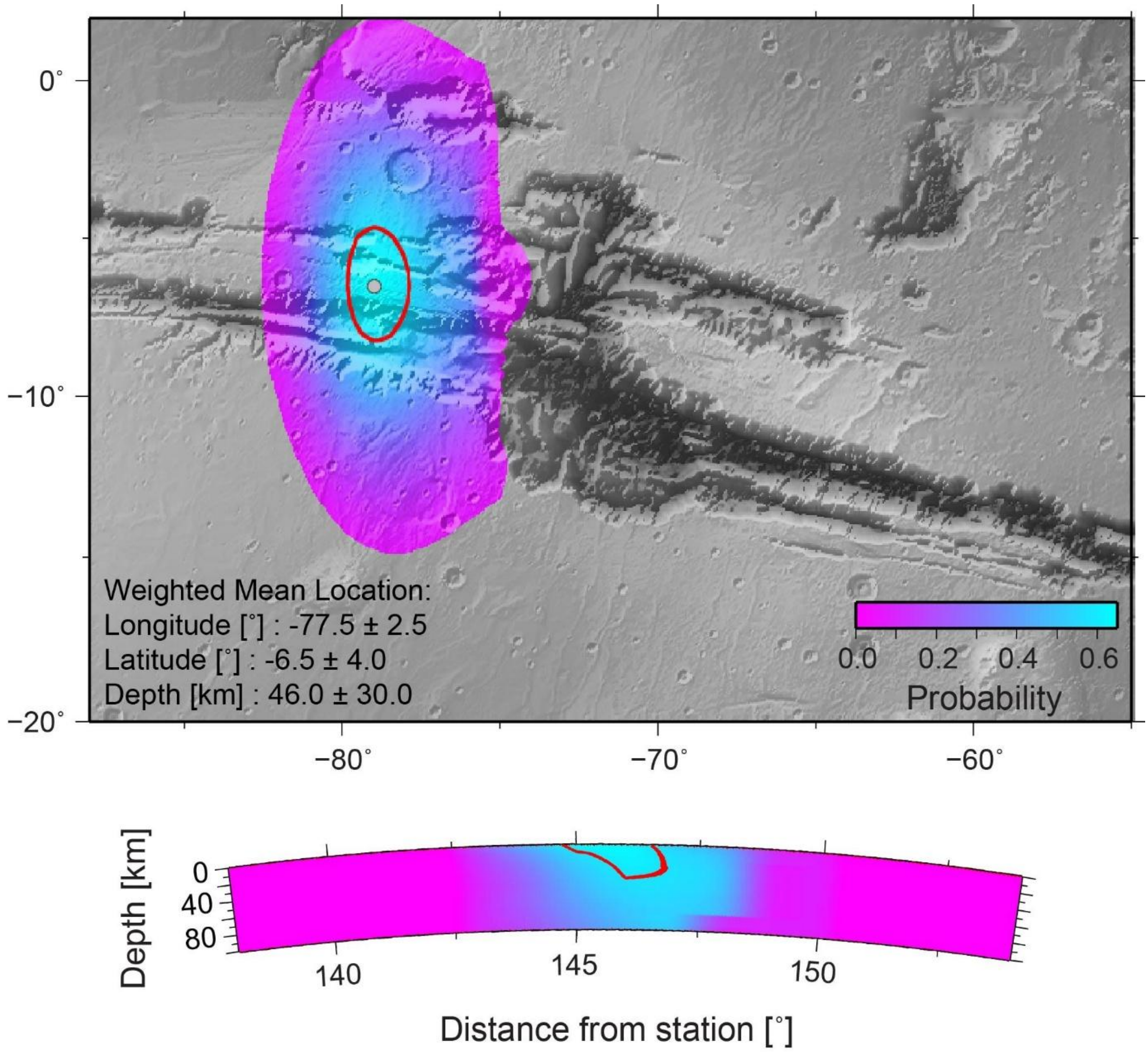

Supplementary Figure 19: Single-station location of marsquake S0976a. (Top) Probability distribution of the inferred source epicentre obtained from a global grid search using back-azimuth, inclination, and S−P differential travel-time constraints (Supplementary Fig. 18). Likelihoods were evaluated on a regular latitude–longitude–depth grid (0.5° lateral spacing; 0–100 km depth range) using Gaussian misfit functions, and combined across multiple one-dimensional Martian velocity models[16,17]. (Bottom) Depth distribution along the great-circle cross-section connecting the station and the maximum-likelihood source location. Contours indicate the 90% confidence region relative to the peak probability.

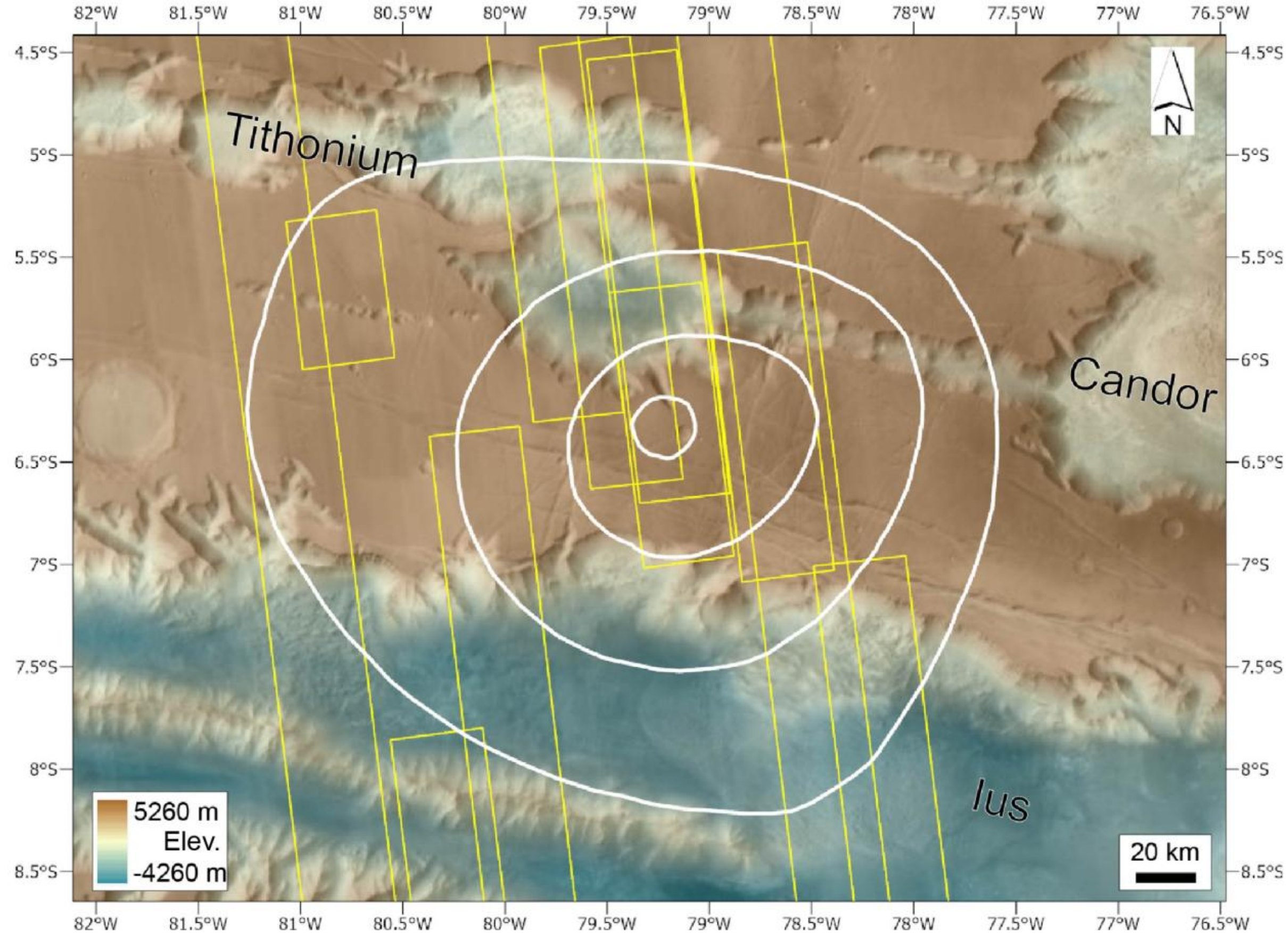

Supplementary Figure 20: Satellite image coverage for landslide assessment in the S0976a source region. The Context Camera (CTX) image footprints (yellow boxes) acquired after August 2021 are overlaid on topography of the Ius–Tithonium–Candor Chasma region. White contours indicate the probability distribution of the inferred source epicentre derived from single-station location analysis, with contours shown from 0.6 (outermost) to higher probabilities at 0.1 increments. The analysed CTX coverage corresponds to all available post-event images within the search region.

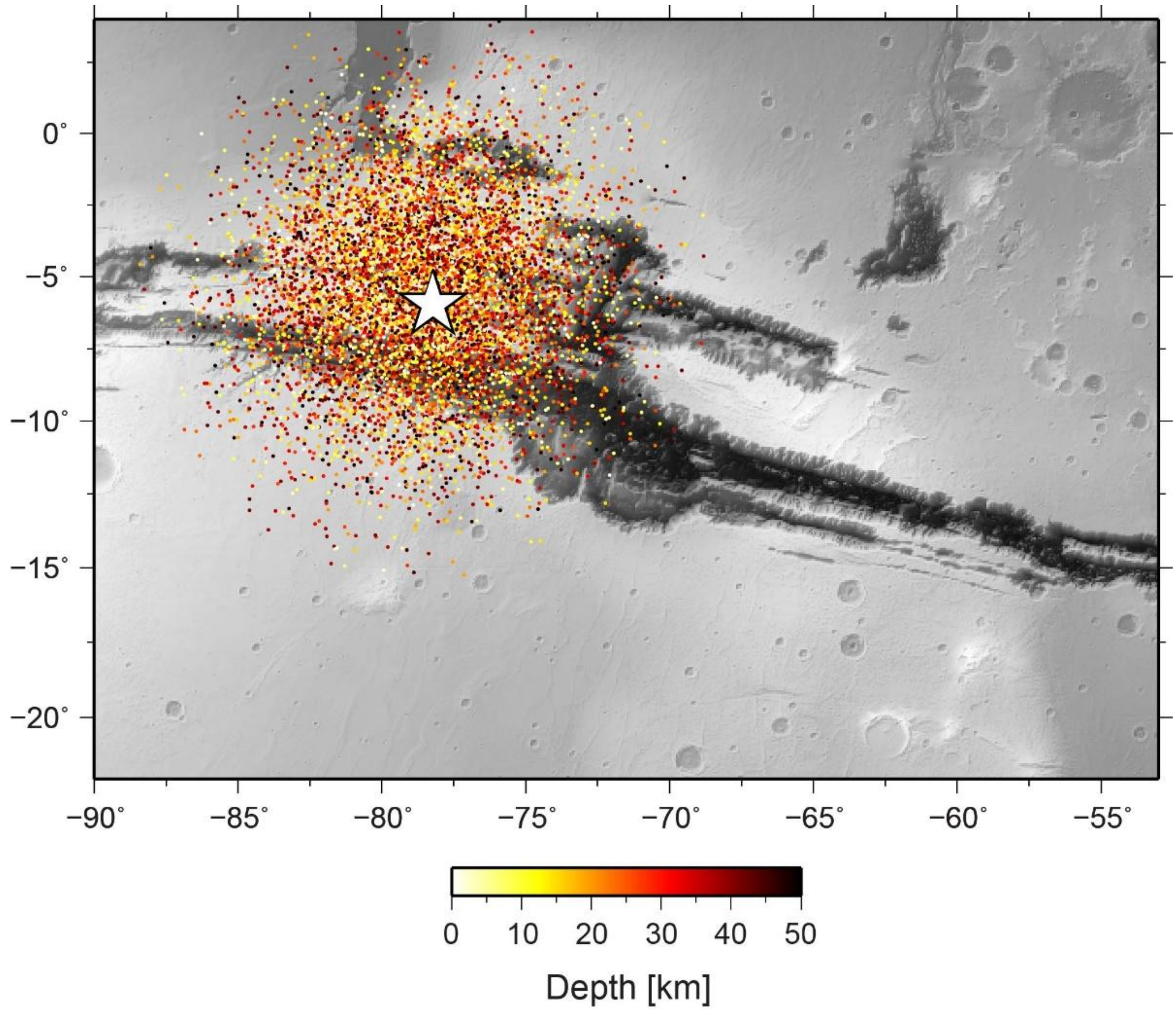

Supplementary Figure 21: Hypothetical aftershock distribution for S0976a. Spatial distribution of hypothetical aftershocks generated using the Epidemic-Type Aftershock Sequence (ETAS) model and conditioned on the S0976a mainshock (see "Methods"). Each point represents an event from Monte Carlo–generated catalogues, with colours indicating focal depth (km). The white star marks the preferred location of the mainshock. Event locations are sampled within a 10° radius of the mainshock epicentre with a Gaussian distance decay (σ = 3°), and depths are uniformly distributed between 0 and 50 km.

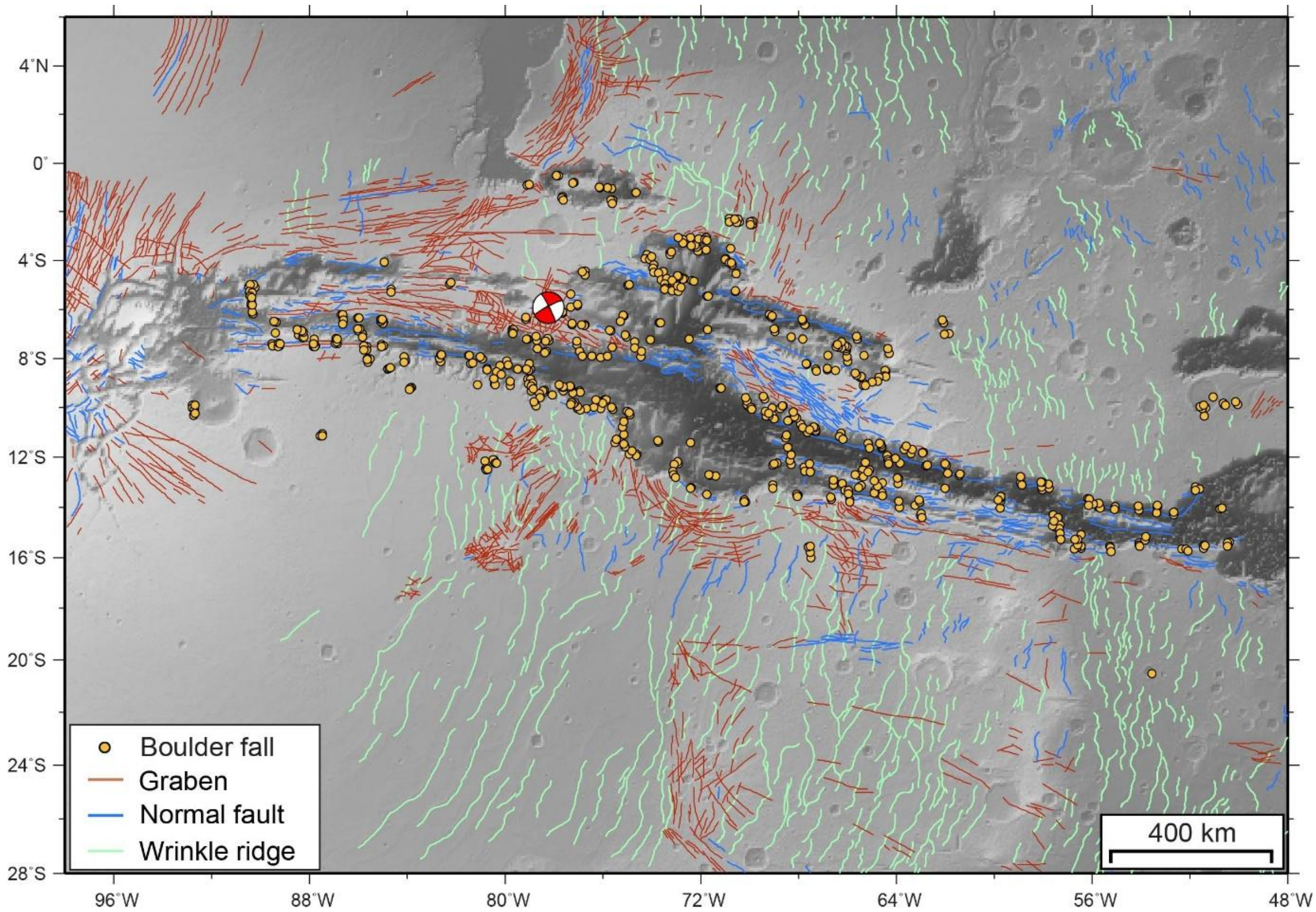

Supplementary Figure 22: Regional structural map of Valles Marineris and surrounding regions. Structural elements, including grabens (brown), normal faults (blue), wrinkle ridges (green), and boulder-fall sites (yellow circles), were digitised from CTX images following and re-evaluating the mapping of Kumar et al.[27]. The focal mechanism of S0976a is shown as a red beachball at the preferred source location. Fig. 4 shows a zoomed-in view of the source area within this region. Background colours represent shaded relief derived from the Mars Orbiter Laser Altimeter (MOLA) topography[28].

References

1. Ogata, Y. Statistical models for earthquake occurrences and residual analysis for point processes. *J. Am. Stat. Assoc.* **83**, 9–27 (1988).
2. Ogata, Y. Space–time point process models for earthquake occurrences. *Ann. Inst. Stat. Math.* **50**, 379–402 (1998).
3. Saichev, A., Helmstetter, A. & Sornette, D. Power-law distributions of offspring and generation numbers in branching models of earthquake triggering. *Pure Appl. Geophys.* **162***,* 1113–1134 (2005).
4. Utsu, T., Ogata, Y. & Matsu'ura, R. S. The centenary of the Omori formula for a decay law of aftershock activity. *J. Phys. Earth* **43**, 1–33 (1995).
5. Ogata, Y. Detection of precursory relative quiescence before great earthquakes through a statistical model. *J. Geophys. Res. Solid Earth* **97**, 19845–19871 (1992).
6. Zhang, L., Werner, M. J. & Goda, K. Variability of ETAS parameters in global subduction zones and applications to mainshock–aftershock hazard assessment. *Bull. Seismol. Soc. Am.* **110**, 191–212 (2020).
7. Helmstetter, A. & Sornette, D. Subcritical and supercritical regimes in epidemic models of earthquake aftershocks. *J. Geophys. Res. Solid Earth* **107**, 2237 (2002).
8. Gutenberg, B. & Richter, C. F. Frequency of earthquakes in California. *Bull. Seismol. Soc. Am.* **34**, 185–188 (1944).
9. Knapmeyer, M. *et al.* The global seismic moment rate of Mars after event S1222a. *Geophys. Res. Lett.* **50**, e2022GL102296 (2023).
10. Båth, M. Lateral inhomogeneities of the upper mantle. *Tectonophysics* **2**, 483–514 (1965).
11. Shcherbakov, R. & Turcotte, D. L. A modified form of Båth's law. *Bull. Seismol. Soc. Am.* **94**, 1968–1975 (2004).
12. Hanks, T. C. & Kanamori, H. A moment magnitude scale. *J. Geophys. Res. Solid Earth* **84**, 2348–2350 (1979).
13. Thurin, J. *et al.* MTUQ: a framework for estimating moment tensors, point forces, and their uncertainties. *Geophys. J. Int.* **241**, 1373–1390 (2025).
14. Horleston, A. C. *et al.* The far side of Mars: two distant marsquakes detected by InSight. *Seism. Rec.* **2**, 88–99 (2022).
15. Ekström, G., Nettles, M. & Dziewoński, A. M. The global CMT project 2004–2010: Centroid-moment tensors for 13,017 earthquakes. *Phys. Earth Planet. Inter.* **200–201**, 1–9 (2012).
16. Khan, A. *et al.* Evidence for a liquid silicate layer atop the Martian core. *Nature* **622**, 718–723 (2023).
17. Samuel, H. *et al.* Geophysical evidence for an enriched molten silicate layer above Mars's core. *Nature* **622,** 712–717 (2023).

18. Knapmeyer-Endrun, B. *et al.* Thickness and structure of the Martian crust from InSight seismic data. *Science* **373**, 438–443 (2021).
19. Khan, A. *et al.* Upper mantle structure of Mars from InSight seismic data. *Science* **373**, 434–438 (2021).
20. Stähler, S. C. *et al.* Seismic detection of the martian core. *Science* **373**, 443–448 (2021).
21. Li, J. *et al.* Constraints on the Martian crust away from the InSight landing site. *Nat. Commun.* **13**, 7950 (2022).
22. Kim, D. *et al.* Structure along the Martian dichotomy constrained by Rayleigh and Love waves and their overtones. *Geophys. Res. Lett.* **50**, e2022GL101666 (2023).
23. Crotwell, H. P., Owens, T. J. & Ritsema, J. The TauP Toolkit: Flexible seismic travel-time and ray-path utilities. *Seismol. Res. Lett.* **70**, 154–160 (1999).
24. Irving, J. C. E. *et al.* First observations of core-transiting seismic phases on Mars. *Proc. Natl. Acad. Sci. USA* **120**, e2217090120 (2023).
25. Sita, M. & van der Lee, S. Potential volcano-tectonic origins and faulting mechanisms of three low-frequency marsquakes detected by a single InSight seismometer. *J. Geophys. Res. Planets* **127**, e2022JE007309 (2022).
26. Knapmeyer, M. *et al.* Working models for spatial distribution and level of Mars' seismicity. *J. Geophys. Res. Planets* **111**, E11006 (2006).
27. Kumar, P. S. *et al.* Recent seismicity in Valles Marineris, Mars: Insights from young faults, landslides, boulder falls and possible mud volcanoes. *Earth Planet. Sci. Lett.* **505**, 51–64 (2019).
28. Smith, D. E. *et al.* The global topography of Mars and implications for surface evolution. *Science* **284**, 1495–1503 (1999).
