# Supplementary material for: Active crustal straining and fault reactivation at Valles Marineris
Source: Nat Commun. 2026 Oct 15;17:10530. doi: 10.1038/s41467-026-78193-6 (PMC13672341; doi:10.1038/s41467-026-78193-6)
Supplement: Supplementary file 2 [file 41467_2026_78193_MOESM2_ESM.pdf]

nature portfolio

Peer Review File

Active crustal straining and fault reactivation at Valles Marineris

Corresponding Author: Dr Jung-Hun Song

Parts of this Peer Review File have been redacted as indicated to remove third-party material.

This file contains all reviewer reports in order by version, followed by all author rebuttals in order by version.

Attachments originally included by the reviewers as part of their assessment can be found at the end of this file.

Version 0:

Reviewer comments:

Reviewer #1

(Remarks to the Author)
Please see the attached comment file.

Reviewer #2

(Remarks to the Author)
See attached pdf

Version 1:

Reviewer comments:

Reviewer #1

(Remarks to the Author)
Please see the attached comment file.

Reviewer #2

(Remarks to the Author)
See pdf

Version 2:

Reviewer comments:

Reviewer #1

(Remarks to the Author)
Minor comment on Fig. 5: I think it would be better to remove the label “Subordinate local transtension”. Given that the inferred mechanism is predominantly strike-slip, and the T-axis orientation alone does not necessarily indicate a significant extensional component, this label may overinterpret the seismic result. Removing it would also make Fig. 5 more consistent with the revised discussion in the manuscript.

Overall, the authors have satisfactorily addressed my previous comments, and I have no further concerns regarding the manuscript. I recommend acceptance for publication after this minor revision.

Open Access This Peer Review File is licensed under a Creative Commons Attribution 4.0 International License, which permits use, sharing, adaptation, distribution and reproduction in any medium or format, as long as you give appropriate credit to the original author(s) and the source, provide a link to the Creative Commons license, and indicate if changes were made.
In cases where reviewers are anonymous, credit should be given to 'Anonymous Referee' and the source.
The images or other third party material in this Peer Review File are included in the article's Creative Commons license, unless indicated otherwise in a credit line to the material. If material is not included in the article's Creative Commons license and your intended use is not permitted by statutory regulation or exceeds the permitted use, you will need to obtain permission directly from the copyright holder.
To view a copy of this license, visit https://creativecommons.org/licenses/by/4.0/

From Reviewer 1:

Review comments on “Active, persistent crustal straining at Valles Marineris” by Song et al.

This manuscript investigates the contemporary seismotectonics of Mars by analyzing the unique marsquake S0976a—the only event detected in the Martian western hemisphere. The authors utilize seismic focal mechanism to constrain present-day crustal deformation at Valles Marineris (VM). They deserve credit for addressing a highly compelling planetary evolution question and for attempting to bridge seismological observations with tectonics. The “paleo-to-modern” comparison is conceptually interesting; exploring whether the contemporary stress field resolved from S0976a aligns with Noachian-aged transtensional stress orientations (~90°–100°) is a valuable approach to evaluating long-term lithospheric stress maintenance on a stagnant-lid planet. Additionally, using the estimated 20–40 km centroid depth to rule out shallow wrinkle-ridge detachments and leveraging the strike-slip focal mechanism to contest traditional models of overthickened crustal gravitational collapse are useful discussion points.

However, the manuscript’s overarching geodynamic narrative hinges entirely on the precise orientation of the resolved P/T axes and the strike-slip nodal planes. Given that these focal parameters are derived from an underdetermined, single-station inversion of free-surface multiples (PP and SS) at a teleseismic distance, I have reservations regarding the accuracy, robustness, and uniqueness of the resolved focal mechanism solution. Single-station focal mechanism inversions on Mars are notorious for severe trade-offs, especially between fault strike and event back-azimuth. If the preferred focal mechanism is affected by minor inversion artifacts or trade-offs, the resulting geologic interpretations regarding selective fault reactivation would be compromised. This concern about solution accuracy, combined with major issues concerning data reproducibility and frequency band selection, means the manuscript requires necessary modifications and technical justification before it can be considered for publication.

1. We thank the reviewer for the detailed technical feedback and for recognising the value of our effort to link InSight seismic observations with the tectonic evolution of Valles Marineris. Before addressing the detailed comments below, we would like to clarify how we have treated the limitations inherent to single-station focal mechanism inversion.

We fully acknowledge that focal mechanism solutions derived from a single station are subject to important trade-offs, particularly among fault strike, epicentral distance, back azimuth, source depth, and velocity structure. For this reason, our analysis was designed to explore the admissible solution space as broadly as possible, rather than to search narrowly around a single preferred model, as is often done in previous moment tensor studies (Brinkman et al., 2021; Jacob et al., 2022; Maguire et al., 2023; Malytskyy et al., 2021; Sita & van der Lee, 2021).

In the present study, we build on our previous approach used in Maguire et al. (2023), extending it through additional tests and a more systematic evaluation of the robustness of the inferred mechanism. We test a wide range of plausible depths, source back azimuths, origin times, source physics, and crustal/interior models in order to identify solutions that remain stable across reasonable assumptions and are least dependent on any one modelling choice. Although trade-offs remain unavoidable, the revised manuscript and Supplementary Information now show more clearly that a strike-slip mechanism is recovered consistently across most tested cases, with a relatively stable strike direction oblique to the valley axis. Thus, our tectonic interpretation is not based on a single inversion result, but on the convergence of multiple seismological tests together with independent geological constraints.

We have revised the manuscript to make these tests, limitations, and supporting evidence more explicit, and have made the data and input parameters available online to improve reproducibility. Revisions made in response to the reviewer's suggestions are marked in **blue text** in the revised manuscript and Supplementary Information, and the relevant locations are indicated in this response letter by line number and figure number where appropriate. In addition, we have submitted a tracked-changes version of the Article, comparing the originally submitted manuscript with the revised version, as a related manuscript file. We hope that these additions address the reviewer's concerns regarding the robustness and reproducibility of the focal mechanism solution.

Below are my detailed comments, which I hope will assist the authors in improving the rigor and overall quality of their work.

2. Indeed, the comments were helpful, and we have addressed each of them in the revised manuscript. Please see also our point-by-point responses to the reviewer's comments below.

Major comments

1. Choice of frequency band, processing transparency, and reproducibility The authors filter the vertical component PP and two horizontal component SS waves between 0.08 and 0.2 Hz in their waveform inversion. However, I have reservations regarding the suitability of this specific frequency band, alongside major concerns about data reproducibility and phase utilization. Horleston et al. (2022) analyzed the spectral characteristics of the S0976a signal and found that "the PP phase includes energy between 2 and 6 s, whereas the SS phase is both higher in amplitude and wider in frequency, extending from 1 to 8 s". The authors' selected frequency range (0.08–0.2 Hz, or 5–12.5 s) only overlaps with a very small portion of the true signal energy documented for these phases (Fig. R1). By filtering out the higher-frequency energy (2–5 s) where the PP and SS amplitudes are most robust, the inversion might

be subject to background noise or artifacts (Fig. R2), thereby severely compromising the reliability of the resulting focal mechanism. The authors need to provide a clearer justification for why this specific range was chosen.

[REDACTED]

Fig. R1. Screenshot of the upper panel of Fig. 4 in Horleston et al. (2022). Shaded blue areas denote the 0.08–0.2 Hz range used in this manuscript.

Here, I filtered the three-component waveforms around the PP and SS arrivals into different frequency bands, and compared body wave energies (Fig. R2). The results show that body wave signal-to-noise ratios are relatively low in the period of 5–12.5 s, compared to those at higher frequencies (3–8 s). Specifically, a prominent low frequency noise artifact occurs between -30 s and 10 s relative to the PP arrival on the radial component. I suspect this localized noise contamination is the primary reason the authors omitted the radial component of the PP wave from their inversion, while all previous source inversions on Mars include radial-component P-waves to properly constrain the focal mechanism (Brinkman et al., 2021; Jacob et al., 2022; Maguire et al., 2023). From my perspective, the 3–8 s period is a better choice as the synthetic seismograms can be reliably computed down to 2 s. Although 3D heterogeneities may complicate the wavefield in this band, this effect requires further evaluation, as the higher signal-to-noise ratio might provide tighter constraints.

The data utilized to generate my replication plot (Fig. R2) were obtained from the data repository of Li et al. (2022), which provides the deglitched three-component displacement records for event S0976a (https://zenodo.org/records/7252035). The twopass, second-order Butterworth bandpass filter was applied. However, the resulting waveforms within the 0.08–0.2 Hz band are quite different from those "emergent yet distinct PP and SS phases" displayed in the manuscript Fig. 2b, except for the radial component SS wave (Fig. R3). Notably, my replicated vertical-component PP wave and transverse-component SS wave exhibit much more prominent, higher-frequency wiggles than the smooth phases presented in Fig. 2b. Are these differences the result of specific preprocessing choices? Or are we looking at quite different time windows? For instance, did the authors implement particular

downsampling, apply narrow-window tapering around the phase arrivals, or perform localized polarization filtering to clean the vertical and transverse component waveforms? To ensure transparency and reproducibility, I suggest the authors clarify these exact data processing steps or make their data publicly available.

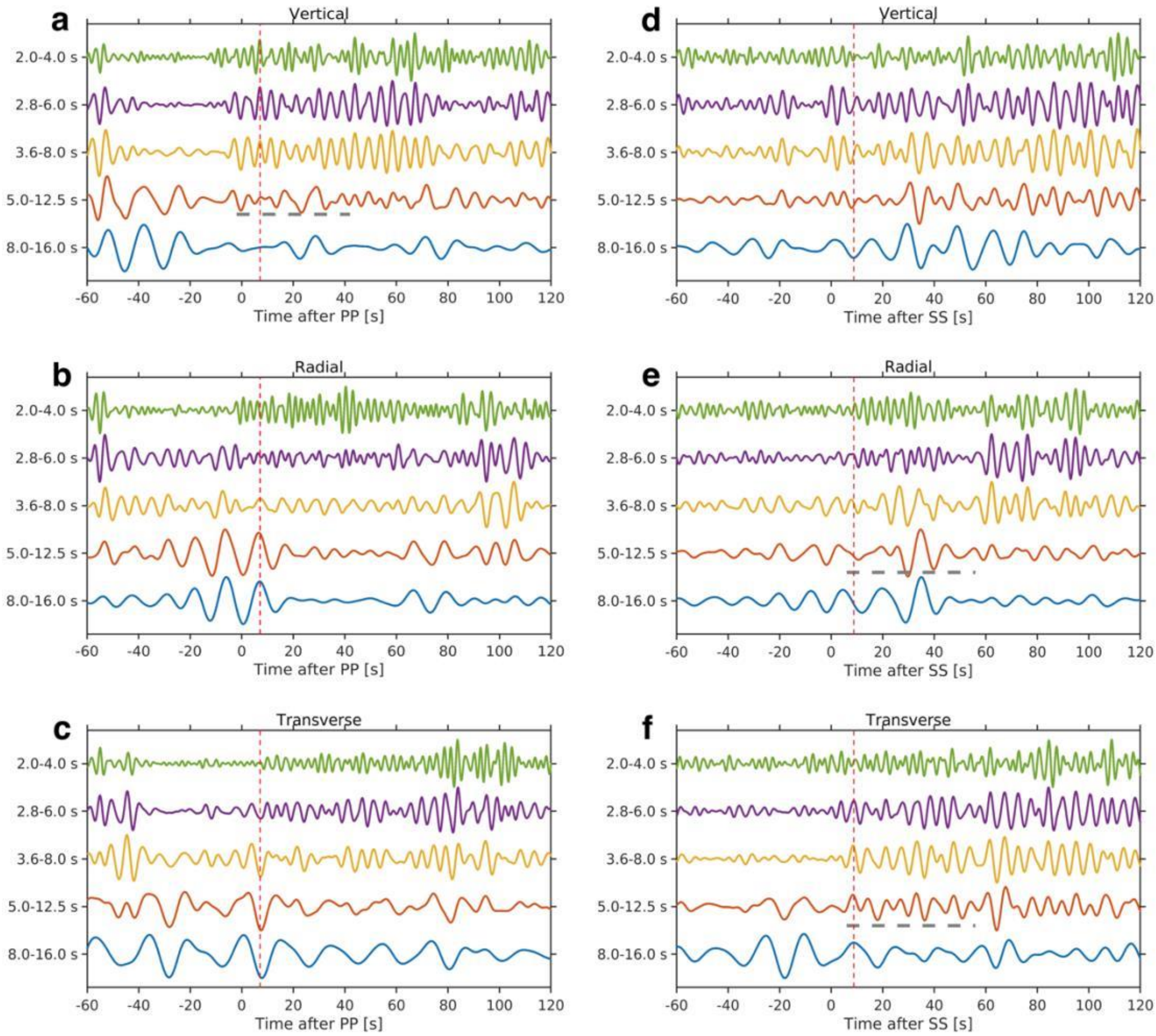

Fig. R2. Three-component waveforms around the PP and SS arrivals filtered into different frequency bands. A back azimuth of 101° is used for waveform rotation (Horleston et al., 2022). The zero times denote the picked PP and SS arrival times in Horleston et al. (2022), while vertical red dashed lines indicate the PP and SS arrival times in Li et al. (2022). Horizontal gray dashed lines represent the selected time windows for waveform comparison with those in Fig. 2b of the manuscript (see Fig. R3).

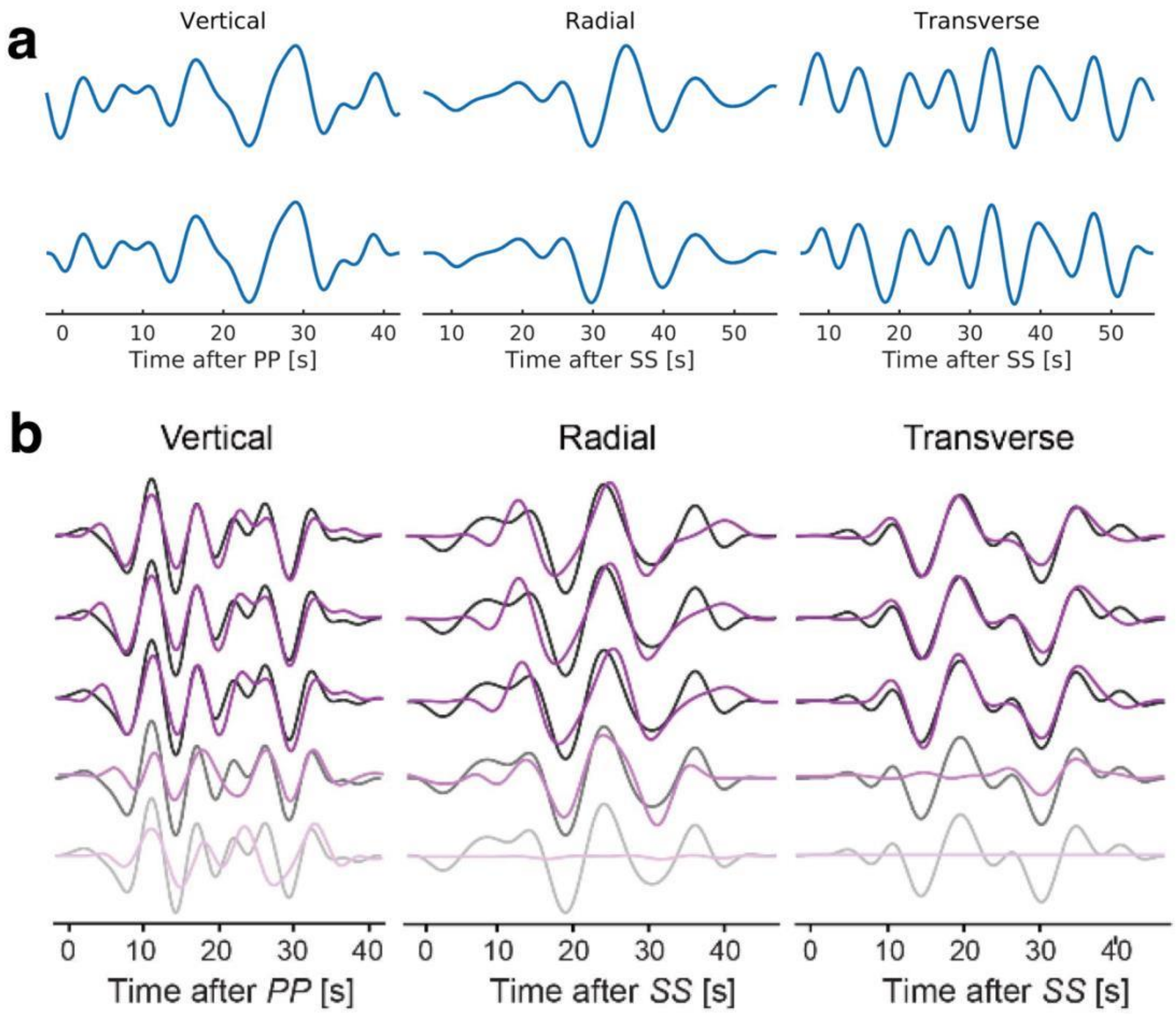

Fig. R3. Waveform comparison of the observed PP and SS waves extracted from my replication pipeline (using the deglitched displacement data from Li et al., 2022) (a) and the corresponding waveforms presented in Fig. 2b of the manuscript (b). The radial component SS wave are quite similar, but the vertical component PP wave and the transverse component SS wave show distinct discrepancies. The lower panels in (a) represent waveforms tapered within a Hanning window with a 10% width at both ends.

3. We agree with the reviewer that the rationale for selecting the 0.08–0.2 Hz frequency band, as well as the details of the waveform preprocessing, were not described sufficiently in the original manuscript. We have therefore expanded the methodological description, added frequency-band sensitivity tests (as a new Fig. 3 in the revised manuscript), and made the waveform data, processing parameters, and inversion inputs publicly available (https://figshare.com/s/f6a720142375dd8fa3d1).

Our initial use of 0.08–0.2 Hz was motivated by the objective of constraining the source mechanism using the coherent long-period energy of the low-frequency event S0976a. This band was not chosen to encompass the full spectral content of the PP and SS phases, nor to maximise their absolute amplitudes. Rather, it was selected to isolate the portion of the signal that we expected to be modelled most reliably with the available synthetic waveforms and one-dimensional velocity models. At higher frequencies, the observed waveforms become increasingly complex (Fig. Res. 1) because of scattering and

propagation effects associated with small-scale crustal heterogeneity that are not fully represented in the Green's functions used for the inversion. Such effects can improve the apparent signal-to-noise ratio (SNR) while still reducing the reliability of the source mechanism constraints.

In response to the reviewer's comment, however, we have now carried out additional inversions over a broader set of frequency bands spanning the available spectral content of S0976a. These tests include both the original 0.08–0.2 Hz band and wider, higher-frequency bands closer to the 0.25–0.5 Hz emphasised by the reviewer. The results, presented in Fig. Res. 1, indicate that waveform complexity increases at higher frequencies and produces localised degradation in the waveform fit. However, the preferred source geometry remains broadly consistent across the tested passbands. In particular, a predominantly strike-slip mechanism is recovered in all cases, with a similar strike orientation oblique to the Valles Marineris trough system. This demonstrates that the main focal mechanism interpretation is not an artefact of the original frequency band choice.

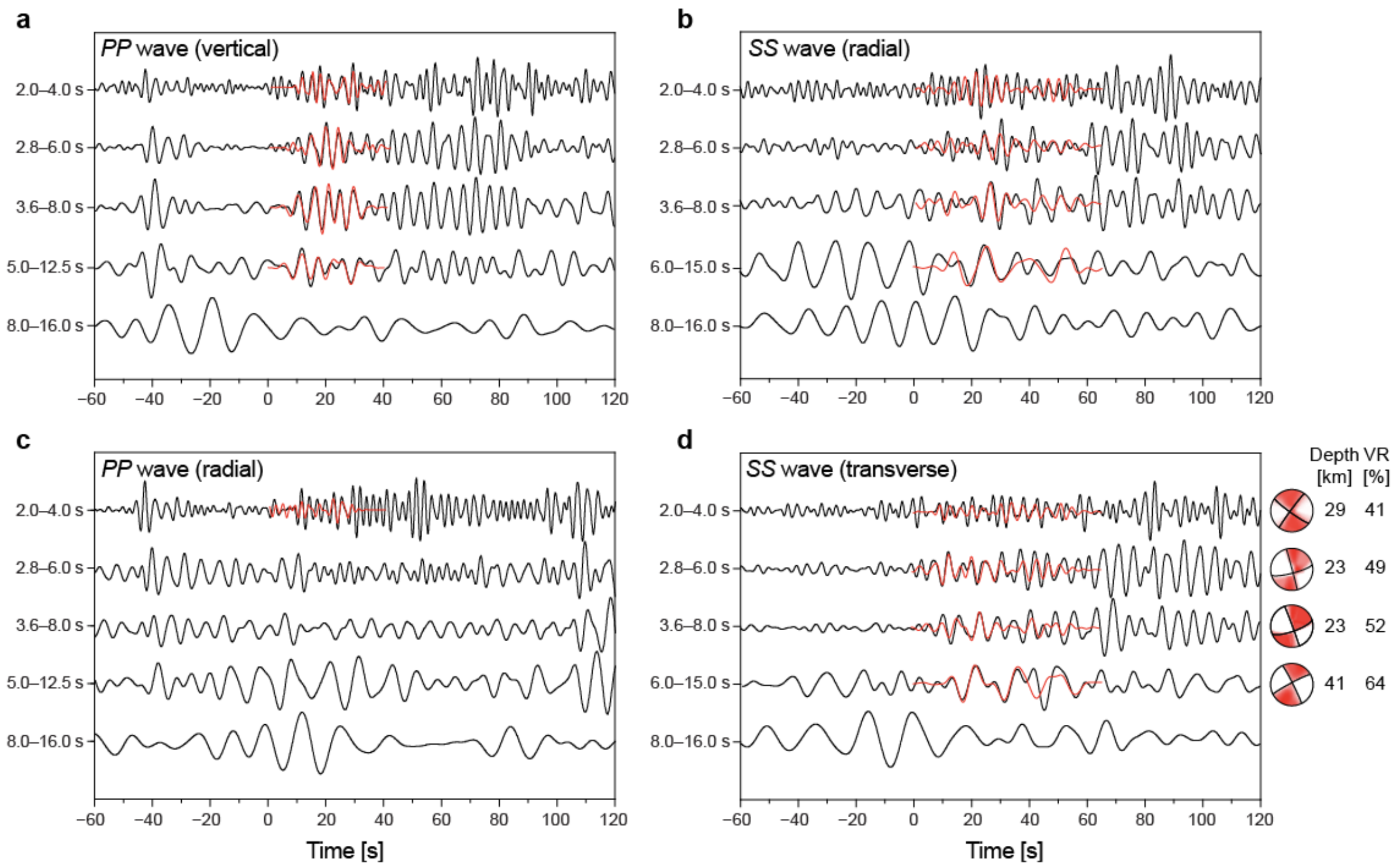

Figure Res. 1. Sensitivity of focal mechanism inversion to the choice of frequency band. Bandpass-filtered seismograms of PP and SS phases are shown for different components: vertical (a) and radial (c) for PP, and radial (b) and transverse (d) for SS. Observed and synthetic waveforms are shown in black and red, respectively. Each row corresponds to an independent inversion using a distinct frequency band: 2.0–4.0 s (top), 2.8–6.0 s, 3.6–8.0 s, and a mixed long-period case (bottom) using 5.0–12.5 s for PP and 6.0–15.0 s for SS. In each case, PP and SS waveforms are jointly inverted. Components or bands not used in the inversion are shown without synthetics. The preferred focal mechanism, centroid depth, and variance reduction (VR) are shown on the right of panel (d). The focal mechanism

represents the ensemble average of strike–dip–rake grid-search solutions with VR > 0.9 × VRmax (where VRmax is the maximum variance reduction obtained among all grid-search solutions), and red quadrants denote compressional (radiating first-motion) polarity. Time is referenced to the aligned synthetic waveform, such that 0 s marks the start of the inversion window after applying the time shift determined during the inversion. Delayed windowing of the long-period SS band (6–15 s) to avoid noise near the theoretical arrival causes an apparent advance of the observed waveforms. The figures are now included as Fig. 3a–d in the revised manuscript.

In our revised manuscript, we have revised the main analysis to use a broader spectral range that better incorporates the higher-frequency energy as suggested by the reviewer. Specifically, the updated inversions use a period range of 3.6–8.0 s for PP and 3.0–14.0 s for SS. We also extended the time windows to include sufficient duration for possible depth phases. These revisions are now reflected throughout the main text, Fig. 2–5, and Supplementary Information (Supplementary Figs. 1–3, 5–9). The updated inversions provide stable fits to PP and SS waveforms across the analysed windows, with fit quality comparable to previous Martian source studies using similar frequency bands, as shown in Fig. Res. 3.

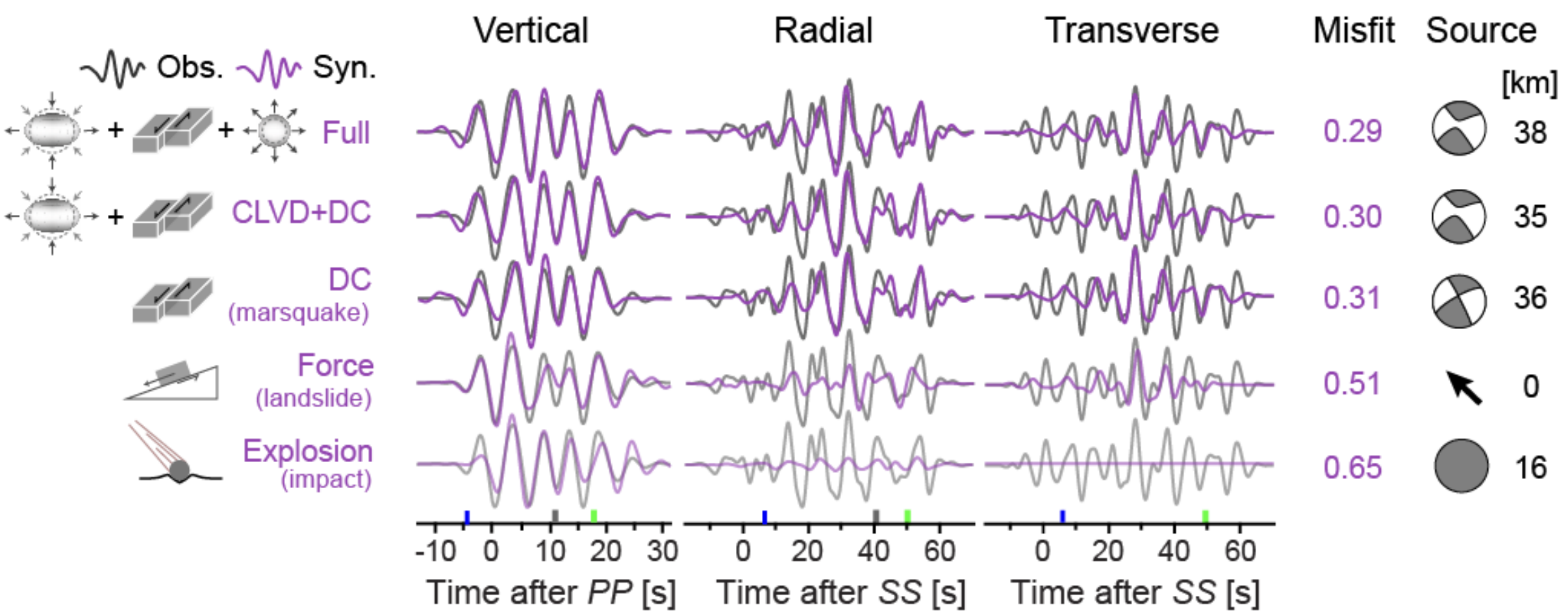

Figure Res. 2. Best-fitting waveform comparisons between observed (black) and synthetic (coloured) three-component seismograms for the source-scenario tests using the revised inversion settings (3.6–8.0 s for PP and 3.0–14.0 s for SS) and extended fitting windows that include possible depth phases. Vertical ticks mark the predicted arrivals of the principal PP/SS phases and possible depth phases, including pPP/sPP and pSS/sSS, computed for the preferred source geometry. For each phase group (PP, SS, and corresponding depth phases), only the earliest (first-arriving) predicted arrival is shown as a representative pick (Supplementary Fig. 13). Time is referenced to the PP and SS picks of Li et al. (2022). Values at right indicate waveform misfit. From top to bottom: full moment tensor inversion, including isotropic (ISO), double-couple (DC), and compensated linear vector dipole (CLVD); CLVD + DC; DC-only; force-based landslide model; and explosion, representing an impact-like source. The

best-fitting solution and corresponding source depth are shown in the rightmost column. This figure replaces the original Fig. 2b.

[REDACTED]

Figure Res. 3. Existing focal mechanism solutions and waveform fitting of Marsquakes constrained using a single seismic station. Shown are representative waveform inversions for events S1222a (Maguire et al., 2023; Malytskyy et al., 2023), S0235b (Brinkman et al., 2021), and S0173a (Jacob et al., 2022). In these studies, the focal mechanism solutions were primarily constrained by fitting the dominant seismic energy occurring ~20–40 s after the principal P- or S-wave arrivals, whereas smaller-scale waveform complexities were not reproduced in detail. To enhance inversion stability, long-period waveforms with maximum periods of 10 s or longer were generally used; for low-frequency events such as S0173a and S0235b, the dominant signal energy lies below ~0.2 Hz. These examples illustrate the level of waveform agreement typically achieved in single-station Marsquake focal mechanism studies, where robust solutions are obtained by reproducing the major phase arrivals and dominant energy packets rather than the complete waveform.

We additionally evaluated the sensitivity of the inversion to the selection of the waveform fitting window. One concern when using relatively emergent PP and SS arrivals is that the preferred focal mechanism could depend on an arbitrary choice of window position rather than on the signal itself. To assess this possibility, we systematically shifted the inversion window relative to the preferred PP–SS alignment by up to ±40 s while keeping the window length fixed. For each shift, the corresponding

synthetic waveform segments were shifted by the same amount, preserving the relative S–P travel-time geometry, and the inversion was repeated.

The results (Fig. Res. 4) show that both VR and focal mechanism stability decrease rapidly as the window moves away from the preferred alignment. Early shifts increasingly sample pre-arrival noise, whereas later shifts are progressively dominated by coda energy. In contrast, the highest VR values and the most stable strike-slip solutions are obtained when the fitting window is centred on the observed PP and SS energy arrivals. These tests indicate that the preferred inversion window is not an arbitrary choice, but corresponds to the portion of the waveform that contains the strongest and most self-consistent source information.

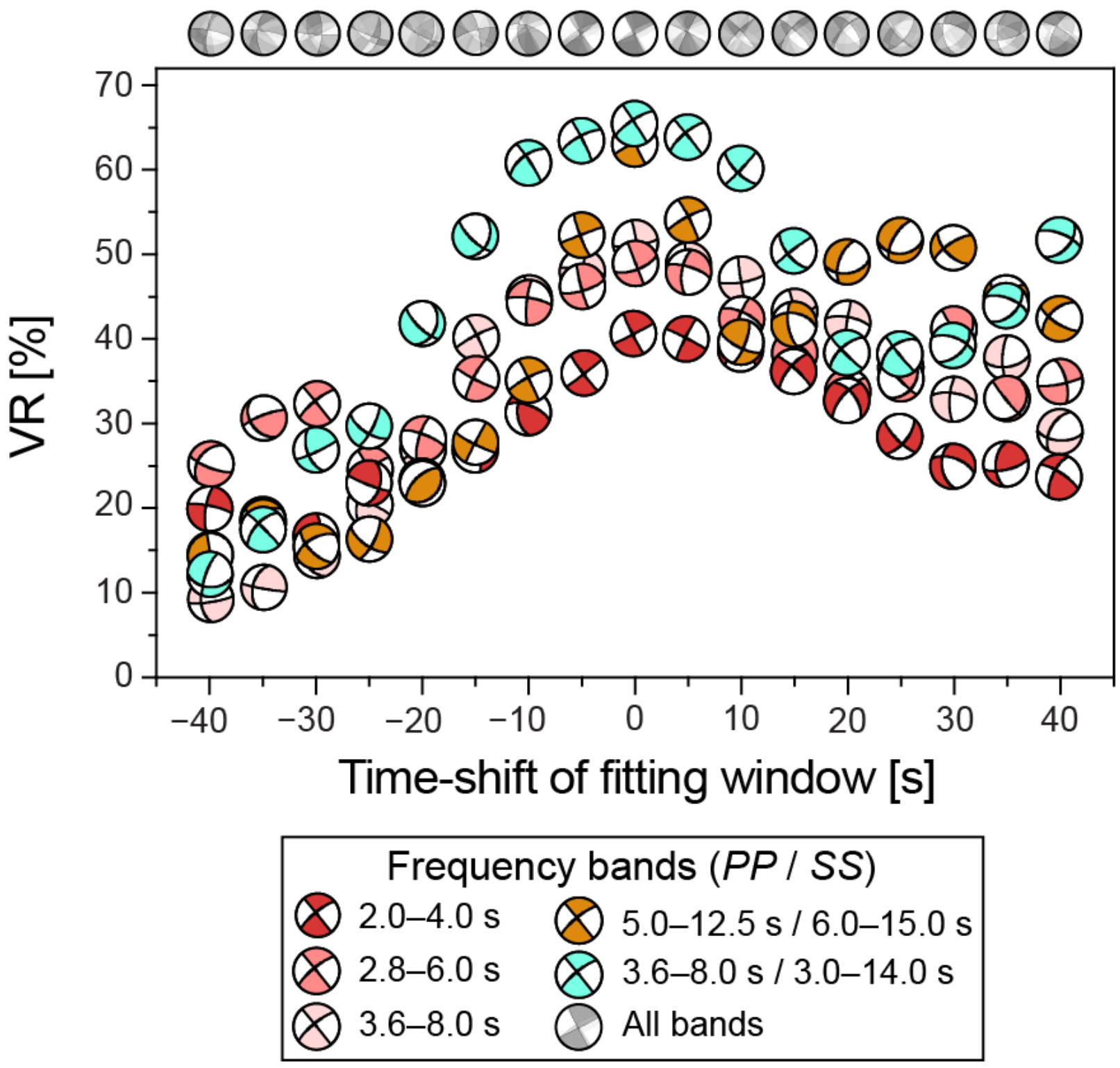

Figure Res. 4. Sensitivity of focal mechanism solutions to fitting-window selection. Focal mechanisms and corresponding variance reductions (VR) were obtained from waveform inversions using systematically shifted fitting windows relative to the preferred PP–SS alignment. Coloured focal mechanisms denote solutions obtained for the different frequency bands shown in Fig. 3a–d, using the preferred centroid depth for each frequency band. Grey, semi-transparent focal mechanisms shown above the panel represent the cumulative ensemble of solutions across all tested frequency bands. This figure is now included as Fig. 3e in the revised manuscript.

We examined the reviewer's Figures R1 and R2 carefully. We agree that the spectral Fig. 4 of Horleston et al. (2022) shows substantial PP and SS energy at periods shorter than those used in our original inversion. However, greater spectral amplitude at shorter periods does not necessarily imply that those frequencies provide more reliable constraints on the source mechanism. The spectral characterisation in Horleston et al. (2022), to which we contributed through the Marsquake Service (https://doi.org/10.12686/a21), was based on a single choice of signal and noise windows that was appropriate for that study. For the present work, we reevaluated the signal-to-noise characteristics using multiple signal and noise windows as part of a broader reassessment of source characteristics across the InSight marsquake catalogue (Song et al., manuscript under review, 2026, https://doi.org/10.22541/essoar.15005266/v1). These results (Fig. Res. 5) show that the PP and SS energy remain identifiable within the frequency bands used in our original analysis, and that their onset times remain stable across the range of tested filters. The differences between the reviewer's replicated waveforms in Fig. R2 from Li et al. (2022) and those shown in our manuscript likely arise from differences in preprocessing choices, including the deglitching/despiking method used, filter types, filter order and tapering.

In the revised manuscript, we now specify these processing steps explicitly (**lines 309–330** in the Methods). In brief, we used a one-pass causal bandpass filter and applied an additional high-pass filter near the lower corner of the passband to reduce long-period noise before the phase arrivals. We have also made the processed waveform data, parameter files, and inversion input files available online (https://figshare.com/s/f6a720142375dd8fa3d1) so that the waveform comparisons and focal mechanism inversions can be reproduced directly.

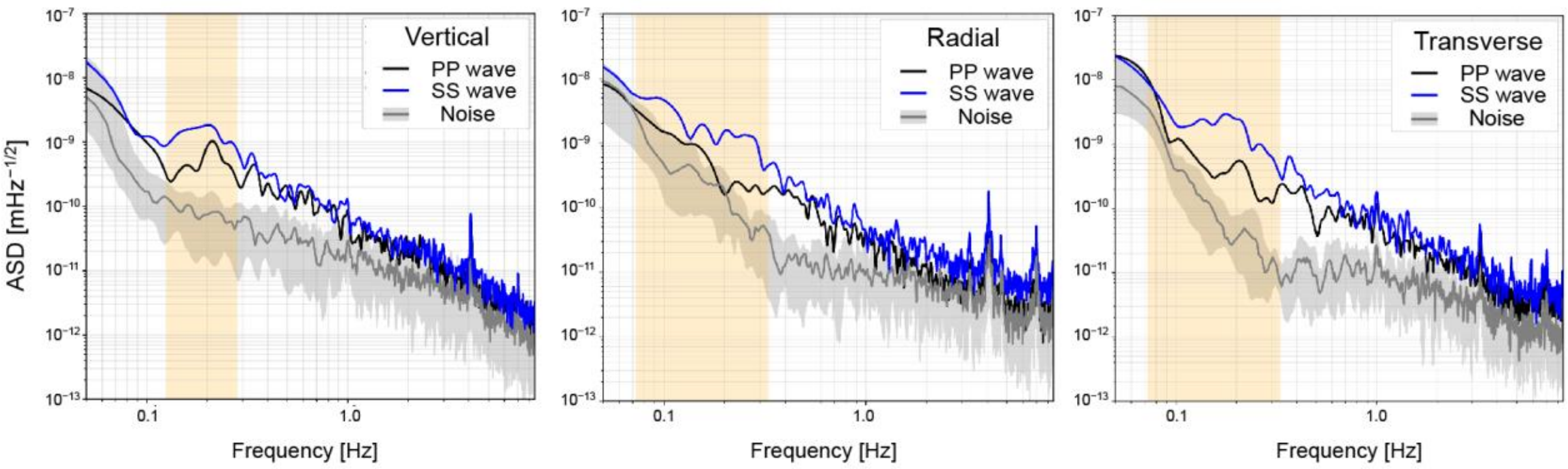

Figure Res. 5. Amplitude spectral density (ASD) of PP (black) and SS (blue) phases of event S0976a for the three components. The ASD of noise (grey) and its standard deviation (light grey) are shown for comparison. The noise window has a duration of ~100 s, and its variability is estimated using a sliding window between −500 s and −50 s relative to the first P-wave arrival, with a step of 20 s. The frequency band used for waveform inversion is highlighted.

Regarding the radial component of the PP arrival, we excluded it from the preferred inversion because the horizontal components are relatively strongly affected by wind-induced noise during this time interval. In addition, because S0976a is a distant event, the radial component contains limited P-wave energy relative to the vertical component. We have performed inversions that explicitly include the radial component to assess its potential contribution. These tests show only marginal improvement in waveform fit and no substantive change in the inferred source parameters or preferred focal mechanism. Furthermore, inclusion of the radial component does not significantly reduce trade-offs in solution space (Fig. Res. 6), particularly with respect to back azimuth. This limited additional constraint is expected for a single-station inversion, where source geometry remains partly tradeable with the assumed event back azimuth.

We have added these synthetic recovery tests to the Supplementary Information (Supplementary Fig. 10) and clarified the basis for excluding the radial PP component from the preferred solution in the Methods (**lines 322–324, lines 392–394**).

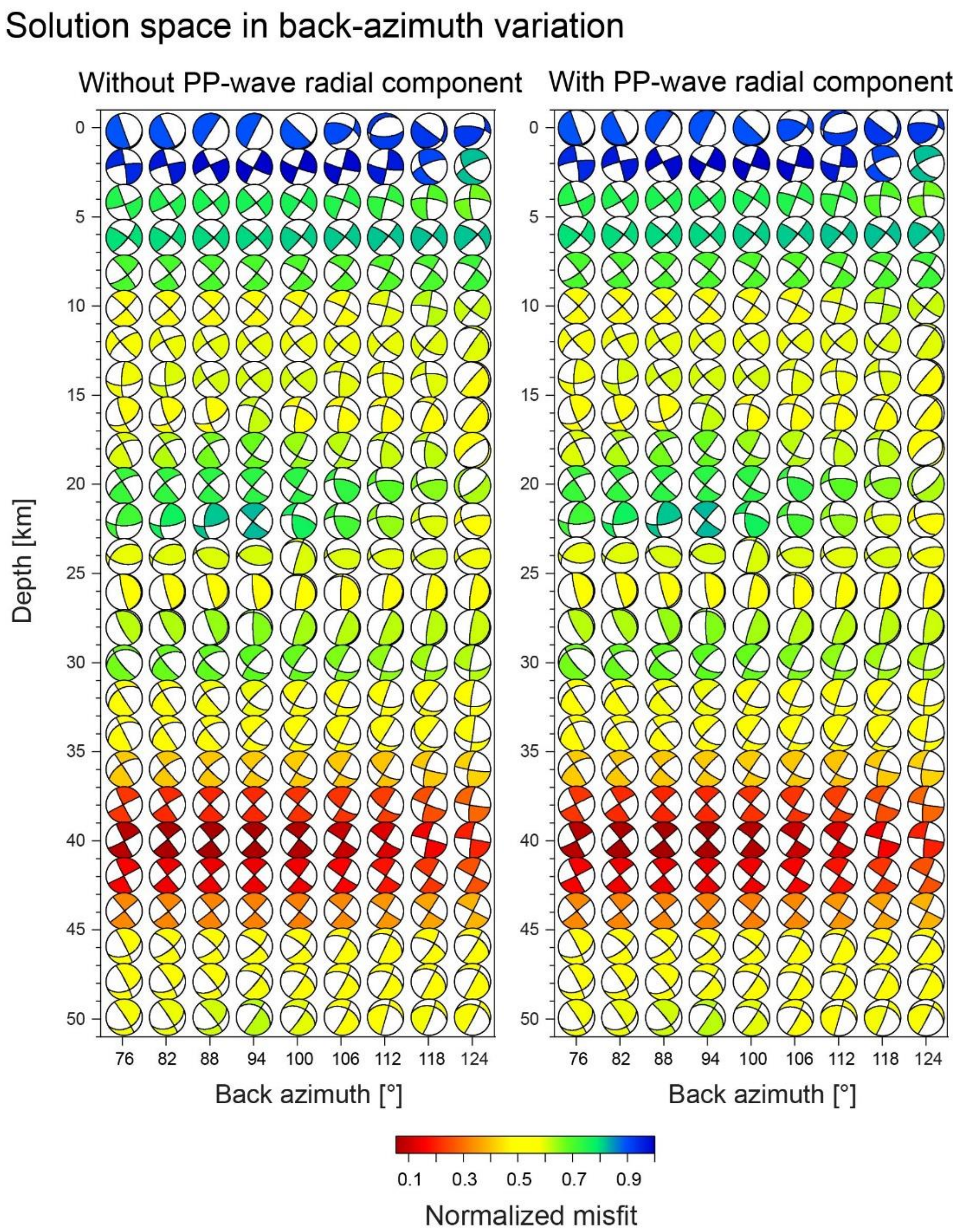

Figure Res. 6. Focal mechanism solution space comparison with and without inclusion of the radial component of the PP wave. Solutions are shown as a function of centroid depth and back azimuth, with epicentral distance held fixed. Beachball colours indicate normalised waveform misfit. Including the radial component results in only minor changes in the misfit distribution and does not significantly alter the preferred solution. The overall solution space remains broadly similar, reflecting the limited additional constraint provided by the radial PP waveform in a single-station inversion, where trade-offs with back azimuth persist. This figure is now included as Supplementary Fig. 10.

For event S0976a, the SKS phase was also identified and picked (Irving et al., 2023). Given that single-station focal mechanism inversions on Mars are severely underdetermined, incorporating additional

SKS phase is crucial, as it samples different take-off angle and provides independent constraints on the source radiation pattern. I suggest the authors include the picked SKS phase in their moment tensor inversion to make the resolved focal mechanism more convincing.

4. We are aware of the SKS phase identified for S0976a, and we agree that, in principle, including an additional phase that samples a different part of the focal sphere would be valuable for a single-station source inversion. However, the SKS arrival for S0976a is extremely weak and difficult to identify in the standard three-component waveforms. Its identification in Irving et al. (2023), to which we contributed, relied on frequency-dependent polarisation analysis in the time-frequency domain, specifically by enhancing horizontally rectilinear motion relative to vertically rectilinear motion. As shown in Fig. Res. 7, the feature interpreted as SKS occurs approximately 310 s after the PP arrival, but its amplitude is close to the noise level.

For this reason, we do not consider the SKS waveform sufficiently robust for inclusion in the moment tensor inversion. In addition to its low SNR, the SKS phase is expected to be strongly affected by propagation through the deep Martian mantle and by possible heterogeneity near the core-mantle boundary. These effects are not represented in the 1-D structural models and Green's functions used in our inversion, and could therefore introduce additional modelling bias rather than improve the source constraint. Moreover, the clearest spectral content of the reported SKS signal lies mainly between 0.5 and 0.8 Hz, which is outside the frequency range over which our synthetic waveforms are modelled.

We have clarified in the main text (**lines 90–92**) that SKS and other additional phases were tested but not included in the preferred inversion, and we include the additional inversion results in the Supplementary Information (Supplementary Fig. 15); these results show no improvement in fit or change in the focal mechanism.

[REDACTED]

Figure Res. 7. Seismic data and frequency-dependent polarisation analysis (FDPA) for S0976a (Irving et al., 2023). Top row: Vertical (Z), radial (R) and transverse (T) bandpass-filtered seismograms (0.3-0.8 Hz) for event S0976a. Second row: Seismograms from the top row windowed around the arrivals of PP (left), SKS (centre) and SS (right). Third row: Vertical component of FDPA analysis for PP, SKS and SS. PP is a vertically polarised signal (circled). Fourth row: Horizontal component of FDPA analysis for PP, SKS and SS. SS and SKS are horizontally polarised signals (circled). Bottom row: Excess linearly polarised energy for PP, SKS and SS.

We performed an additional waveform inversion that explicitly included the proposed SKS time window on the radial component. The inversion used the same frequency bands and fitting windows for PP and SS as in the revised preferred solution shown in Fig. 2b of the revised manuscript, while the source location and depth were fixed to the corresponding best-fitting values. An additional waveform window centred on the proposed SKS arrival was then included in the inversion. These tests showed that the SKS signal remains barely distinguishable from the surrounding noise (Fig. Res. 8). While synthetics can be loosely aligned with the observed fluctuations, the corresponding amplitudes are significantly lower relative to the noise level than those of the PP and SS phases, making the fit non-diagnostic. Incorporating SKS does not lead to any measurable change in the preferred focal mechanism, nor does it improve the overall waveform misfit. Instead, it introduces an additional fitting window whose quality cannot be evaluated robustly.

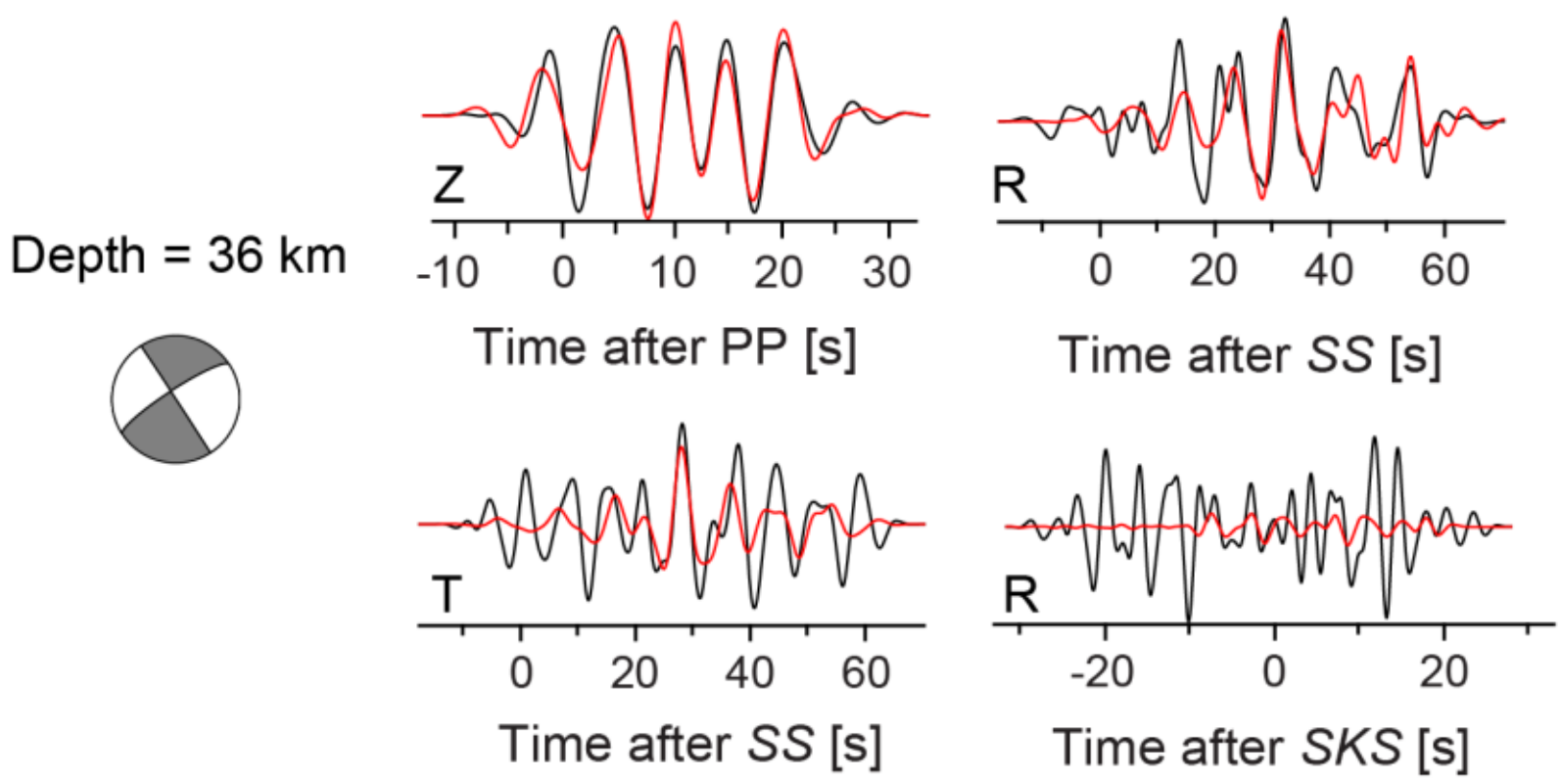

Figure Res. 8. Focal mechanism inversion including the SKS phase on the radial component. The frequency band and time windows for PP and SS are identical to those used in Fig. 2. For SKS, a band-pass filter of 2–5 s was applied, and the time window was selected based on the FDPA-derived arrival (Fig. Res. 7; Irving et al., 2023). Observed and synthetic waveforms are shown in black and red, respectively. The resulting focal mechanism (left) is consistent with the preferred solution obtained without SKS. Despite inclusion of the SKS window, the waveform fit in this phase remains ambiguous due to its low signal-to-noise ratio and does not provide additional constraints on the source. This figure is included as Supplementary Fig. 15.

2. Lack of rigorous uncertainty quantification for the focal mechanism solution. The manuscript heavily relies on the stable orientation of the P/T axes and the resolved strike-slip nodal planes to interpret the mechanical reactivation of the NE-striking rift-oblique faults. However, single-station body-wave moment tensor inversions on Mars are inherently underdetermined and subject to severe trade-offs between back-azimuth and strike (Supplementary Figs. 4, 6, and 7).

While the authors state that the solution ensemble is “stable” or “tightly clustered”, they do not provide a rigorous, quantitative uncertainty analysis for the focal parameters, especially the strike. Supplementary Fig. 4 shows that there exists a systematic variation between the strike and back azimuth. Considering the substantial location uncertainties inherent to Martian seismic events, particularly regarding single-station back-azimuth determinations, this covariance is deeply concerning. If the nodal plane orientations vary by ±15°–20° within a reasonable confidence interval (e.g., 90% or 95% VR thresholds), the kinematic interpretation could shift dramatically. To strengthen the manuscript’s conclusions, I suggest the authors explicitly quantify these parameter trade-offs and map out the formal confidence bounds of the focal mechanism solutions. For example, a minor perturbation in the nodal plane orientation could align the rupture plane subparallel to the main E-W/WNW-ESE surface faulting trends. Mechanically, if a nodal plane becomes subparallel to the Tharsis-driven σ1, the resolved shear stress drops to near-zero, which would completely alter the tectonic narrative proposed in this study. To demonstrate that their structural and geodynamic interpretations are robust against inherent inversion errors, the authors should supply a complete uncertainty and misfit landscape for the focal mechanism parameters.

5. As noted in our Response #1, we fully recognise that single-station moment tensor inversions are subject to important trade-offs, particularly between source back azimuth and fault strike. This concern motivated us to explore not only the source-geometry parameters, but also uncertainties associated with interior velocity structure and source physics, including different assumptions about the nature of the source. By expanding the search in this way, we aimed to reduce the risk that the preferred mechanism reflects a particular modelling assumption rather than a stable feature of the data.

The focal mechanism shown in Fig. 2a is not based on a single best-fitting solution, but on an ensemble of acceptable solutions with VR values greater than 0.9 × VRmax. Source locations were sampled across the published uncertainty range for S0976a, including epicentral distances of 146° ± 7° and back azimuths of 101° ± 25° (Horleston et al., 2022). Depths were explored from 0 to 50 km at 1-km intervals and source magnitudes were varied between Mw 3.3 and 4.0. For each source configuration, we performed a full focal-mechanism grid search using 60 × 60 × 60 strike-dip-rake combinations and calculated waveform fit variance reductions for each model. The accepted solutions span epicentral distances of 142°–150° and back azimuths of 76°–124°, showing that the preferred strike-slip mechanism is recovered for source locations covering a large fraction of the published uncertainty range.

To address the reviewer's concern about whether the nodal-plane orientations used in our structural interpretation are stable, we considered all acceptable focal mechanisms and projected them onto the Kaverina source-type diagram (Fig. Res. 9). This analysis shows that 82.2% of the acceptable solutions

fall within the strike-slip family, including the SS (strike-slip), SS-R (strike-slip reverse), and SS-N (strike-slip normal) classes. Thus, despite the expected uncertainty in the single-station inversion, the acceptable-solution ensemble is dominated by strike-slip motion.

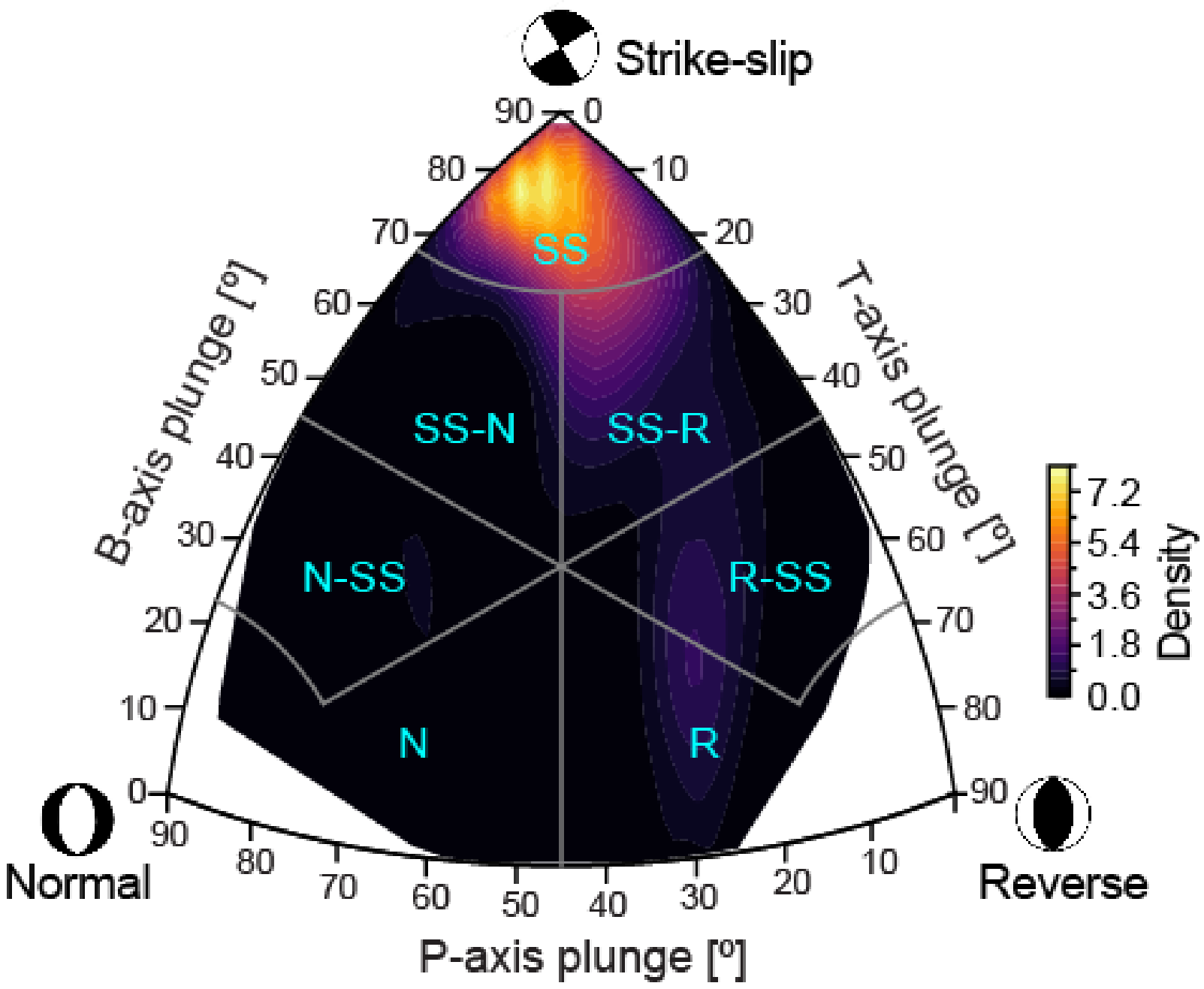

Figure Res. 9. Source-type distribution of acceptable focal mechanism solutions projected onto the Kaverina diagram (Kaverina et al., 1996). Solution density indicates the relative population of source types within the ensemble. The majority of solutions (82.2%) fall within the strike-slip domain, including SS (strike-slip), SS-N (strike-slip with normal component), and SS-R (strike-slip with reverse component) classes, indicating a clear dominance of strike-slip mechanisms despite trade-offs inherent to the single-station inversion. Other source types—N (normal), R (reverse), N-SS (normal with strike-slip component), and R-SS (reverse with strike-slip component)—are only sparsely populated. This figure is now included as Fig. 3f in the revised manuscript.

We also quantified the strike distributions of the nodal planes from the acceptable solution ensemble (Fig. Res. 10). Because strike-slip mechanisms produce two conjugate nodal planes, we separated the two strike populations using k-means clustering in axial-data space that accounts for the 180° periodicity of strike. We then calculated circular statistics for each population. The two nodal-plane strike clusters are centred at 48.0° ± 21.5° and 143.5° ± 22.5°, where the quoted uncertainties correspond to circular standard deviations. These values reflect the expected conjugate fault-plane ambiguity, but both strike populations remain concentrated around NE–SW and NW–SE orientations. The dip and rake distributions are similarly concentrated, with a median dip of 79.0° (16th–84th percentile range: 62.5°–

86.5°) and rake clusters centred at 8.0° ± 26.0° and 174.0° ± 24.5°, indicating that most acceptable solutions correspond to steeply dipping, predominantly strike-slip faulting.

Importantly, the acceptable solutions do not migrate toward the dominant E–W to WNW–ESE valley-parallel fault trends suggested by the reviewer, even when the full range of location, depth, back azimuth, and magnitude uncertainties is included. The trade-off between back azimuth and strike broadens the strike distributions, as expected, but it does not shift the ensemble toward an alternative fault geometry that would fundamentally change the tectonic interpretation.

We have updated the main text (**lines 95–102**) and Methods (**lines 355–362**) to include a detailed description of the ensemble-based inversion approach and the computation of parameter uncertainties. We have also revised Fig. 4 (originally Fig. 3) to show nodal-plane uncertainty bounds and the variability of acceptable solutions, added a source-type classification of the ensemble as Fig. 3f, and provided comprehensive quantitative statistics of the full ensemble, including the total number of explored models, the number of acceptable solutions, and the resulting strike, dip, and rake distributions, in Supplementary Table 1 and Supplementary Fig. 4.

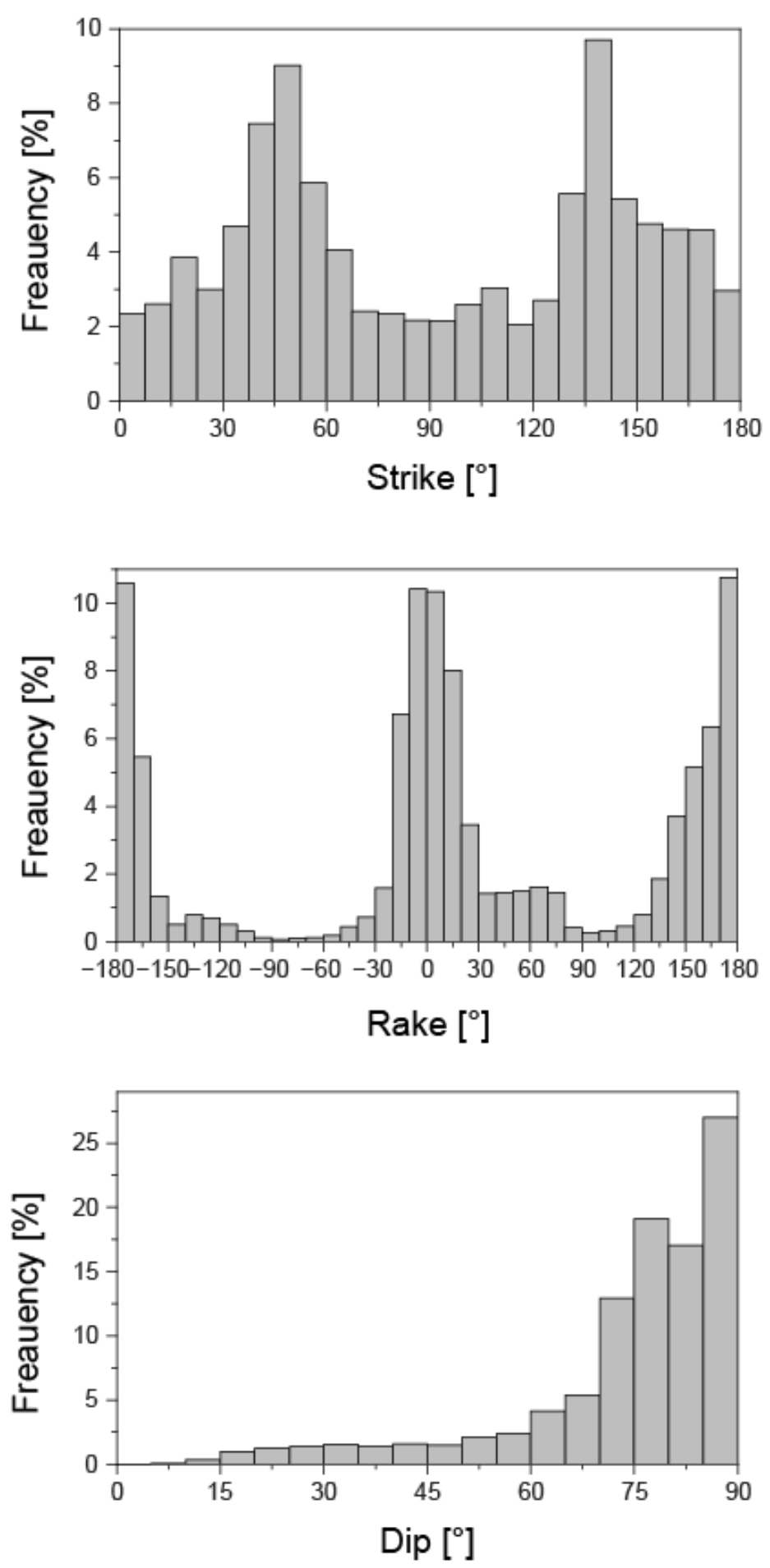

Figure Res. 10. Histograms showing the distributions of strike, dip, and rake for acceptable focal-mechanism solutions with VR > 0.9 × VRmax. The ensemble was extracted from the full grid search over strike, dip, rake, source depth, and epicentral location (see Methods for the complete parameter ranges explored). Summary statistics for the strike, dip, and rake distributions are provided in Supplementary Table 1. This figure is now included as Supplementary Fig. 4.

Minor comments

1. Lines 6–7: In addition to the cited references, Ceylan et al. (2023) investigated Martian seismicity and inferred that three distant events (S0395a, S0133a, and S0154a) may also be situated within the VM region. Given its direct relevance to distant marsquake seismicity, the authors should cite this study here.

6. Thank you for pointing out Ceylan et al. (2023), which is directly relevant to the discussion of distant marsquake seismicity and to which we contributed. We have now added this reference in the revised manuscript (**line 8** in the revised manuscript).

We note, however, that the possible association of S0395a, S0133a, and S0154a with the Valles Marineris region in Ceylan et al. (2023) was based primarily on envelope similarity between events, rather than on direct phase picking of both P and S arrivals. For this reason, we prefer not to treat these events as securely located within Valles Marineris in the same way as S0976a. In addition, ongoing re-evaluation within the Marsquake Service suggests that at least one of these events, S0133a, is now relocated substantially closer to the InSight landing site, at an epicentral distance (27.5°) more comparable to events from the Cerberus Fossae region. Because this updated catalog analysis is not yet published, we have avoided making a strong statement based on it in the manuscript.

2. Lines 34–35: What do the authors mean by the phrase "lithospheric structure of marsquake source regions"? To my understanding, current Martian interior structure models either focus beneath the InSight lander site or represent a broad, large-scale average of the Martian globe. The localized lithospheric structures specifically beneath source regions are generally not resolved independently.

7. We agree that localised lithospheric structures beneath individual marsquake source regions are not independently resolved by current Martian interior models. To avoid overstating the capability of single-station seismic observations, we replaced "lithospheric structure of marsquake source regions" with "marsquake source mechanisms and their tectonic context" (**line 39** in the revised manuscript).

3. Line 72: Please provide the specific value or range of the Moho depth in the source region for comparison.

8. We have revised the text to explicitly state the estimated depth of the crust–mantle boundary beneath western Valles Marineris. Gravity and topography inversions constrained by InSight seismic observations suggest a crustal thickness of approximately 55–65 km in this region (Kim et al., 2023).

The revised sentence now reads (**lines 74–75**):

"The best-fitting centroid depth lies within the middle-to-lower crust, above the estimated Moho depth of ~55–65 km beneath western VM (Kim et al., 2023)."

4. Lines 70–72 and Fig. 2: The authors state in the text that "The best-fitting centroid depth places the source within the mid-crust, at 20–40 km depth". However, according to Fig. 2, the best-fitting centroid depths vary with the velocity model used, resolving at 30–50 km for the Khan model and 20–40 km for the Samuel model. Because the crustal thickness in the Samuel model is substantially greater than that in the Khan model, these depth ranges imply that the source is located either within the lower crust for the Khan model or within the middle-to-lower crust for the Samuel model (Supplementary Fig. 10). The authors should correct the main text to reflect this mode dependence. Furthermore, are there any geomechanical and rheological implications of these differing structural contexts that warrant discussion?

9. The reviewer is correct that the inferred depth depends on the assumed velocity and crustal structure model, and that this model dependence was not stated clearly enough in the original manuscript. We have revised the text accordingly.

The depth range originally quoted in the main text was based primarily on our reference model from Khan et al. (2023), in which the crustal structure is constrained mainly by receiver-function analysis at the InSight landing site. Using the revised inversion settings and misfit definition, this model yields preferred centroid depths of approximately 15–45 km. In contrast, the model of Samuel et al. (2023), which is perhaps more representative of an average global crustal structure based on geodynamic parameterisation, yields preferred depths of approximately 20–40 km.

We have now revised the manuscript (**lines 75–77**) to state these model-dependent depth ranges explicitly and to clarify that they place the source within the lower crust for the Khan et al. (2023) model and within the middle-to-lower crust for the Samuel et al. (2023) model, as shown in Supplementary Fig. 12. The reported depth ranges were defined from the depth-dependent normalised misfit, derived from the VR using a common min–max scaling across all tested interior models. The preferred depth interval for each model was then identified as the range over which the normalised misfit remained

below 0.5. For completeness, we also tested an additional crustal model based on Li et al. (2022), who reported the depth to an intracrustal interface near the PP and SS bounce points of S0976a, combined with the mantle structure of Khan et al. (2023). This test yields source depths within the same broad range of 25–45 km and recovers a consistent strike-slip focal mechanism (Fig. Res. 11).

We have also added a brief discussion of the geomechanical implications of these model-dependent depth estimates in the revised manuscript (**lines 242–245**). Although the precise structural context depends on the assumed crustal model, the inferred depths of several tens of kilometres are mechanically plausible on Mars because its cold, thick stagnant-lid lithosphere can retain frictional strength into the middle-to-lower crust (Jiménez-Díaz et al., 2021).

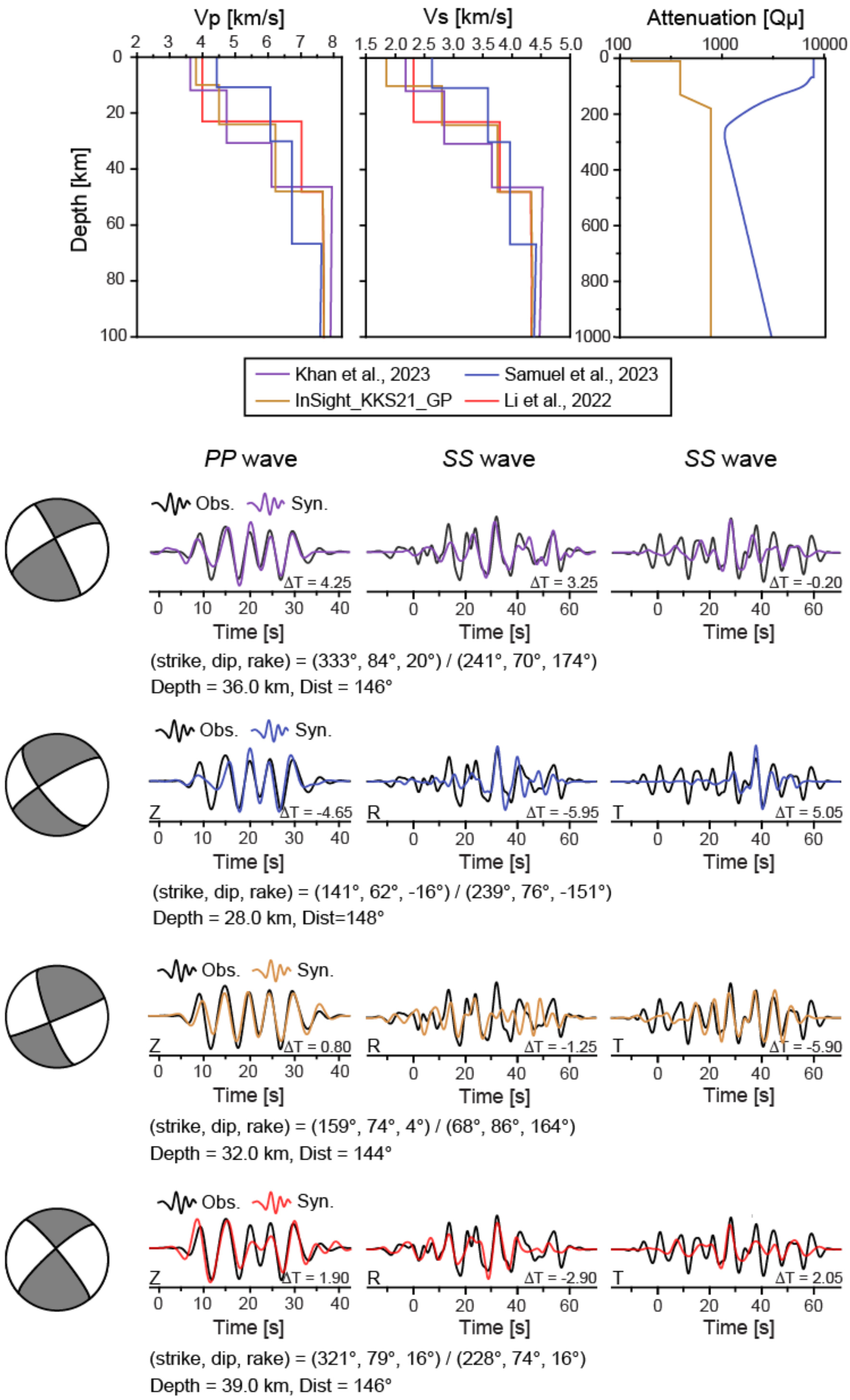

Figure Res. 11. Focal mechanism solution and waveform fitting using different Martian interior models. Comparison between observed and synthetic waveforms computed using four 1-D Martian velocity models (top): Khan et al. (2023), Samuel et al. (2023), the InSight KKS_GP21 model (a combined model based on early InSight mission data; Knapmeyer et al., 2021; Khan et al., 2021; Stähler et al., 2021), and Li et al. (2022). PP phases are shown on the vertical (Z) component, whereas SS phases are compared on the radial (R) and transverse (T) components. Δt indicates the optimal time shift. For each

velocity model, focal-mechanism solutions were searched over source distance and depth while fixing the back azimuth to the preferred value (~93°). The corresponding best-fitting source depth is indicated. For the shear quality factor (Qs) in the Khan et al. (2023) model, we adopt the parameterisation of Samuel et al. (2023). This figure is now included as Supplementary Fig. 12.

5. Fig. 2b: Please state explicitly in the figure caption which model (the Khan model or the Samuel model) is used to generate the displayed synthetic waveforms. For cases of "Full", "CLVD+DC", and "DC", the author should also provide the corresponding centroid depth values.

10. We have now explicitly stated in the Fig. 2b caption that the synthetic waveforms were generated using the Khan et al. (2023) model. We have also calculated and included the centroid depth values for each source scenario ("Full", "CLVD+DC", and "DC") in the revised figure caption.

6. Lines 77–82: I'm surprised that small shift in distance would lead to dramatic variation of the focal mechanism. Because a large time shift is explicitly permitted during the waveform fitting process, any minor travel-time discrepancies introduced by a small distance shift should be easily absorbed by the cross-correlation time alignment. If the focal mechanism remains this hyper-sensitive to minor distance variations despite the flexible time shifts, it may imply an instability or non-uniqueness in the inversion. To substantiate this behavior and demonstrate wavefield stability, the authors should include a supplementary figure displaying a record section of synthetic waveforms across a reasonable distance range. In addition, rather than relying on previously determined distance interval of 146° ± 7°, I suggest the authors adopt a distance range calculated directly from the observed SS-PP differential travel time (incorporating its picking uncertainty) with the adopted velocity models (the Khan and Samuel model).

11. We agree with the reviewer that, if a change in epicentral distance only introduced a simple arrival-time perturbation, this effect should be largely absorbed by the cross-correlation time alignment used in the waveform fitting. However, the distance dependence in our inversion is not limited to a uniform time shift. Changes in epicentral distance also affect the take-off angles, radiation pattern sampling, the timing of possible depth phases, and the relative amplitudes of the modelled phases. These effects cannot be fully corrected by allowing an independent time shift during waveform alignment, as illustrated by the apparent phase shifts in the synthetics shown in Fig. Res. 12. Importantly, solutions at distances further from the preferred event location generally yield lower VR values (Fig. Res. 13), which provides an additional basis for filtering them out of the acceptable solution ensemble.

The adopted distance range (139°–153°) was chosen to broadly explore the parameter space around the previously reported epicentral distance, rather than to rely on a specific prior model. Importantly, the distance estimates derived from recent velocity models fall well within this explored range. For example,

Samuel et al. (2023) reported 144.0° ± 5.3°, and the estimate based on SS–PP differential travel times using the Khan et al. (2023) model yields 145.0° ± 3.2°. These ranges are fully encompassed within our inversion grid and are therefore already represented in the ensemble of solutions. Accordingly, the preferred focal mechanism and the dominant characteristics of the acceptable solution ensemble are not sensitive to the choice of the initial distance range.

We have revised the Methods to clarify that the grid encompasses both the previously reported location uncertainty and the SS–PP differential-time distance estimates obtained using recent Martian interior models (**lines 459–460**). This makes explicit in the manuscript that the adopted search range already includes the model-based distance estimates requested by the reviewer. We also added the new Supplementary Figs. 13 and 14 to show distance-dependent waveform variations of PP and SS phases and the corresponding VR values.

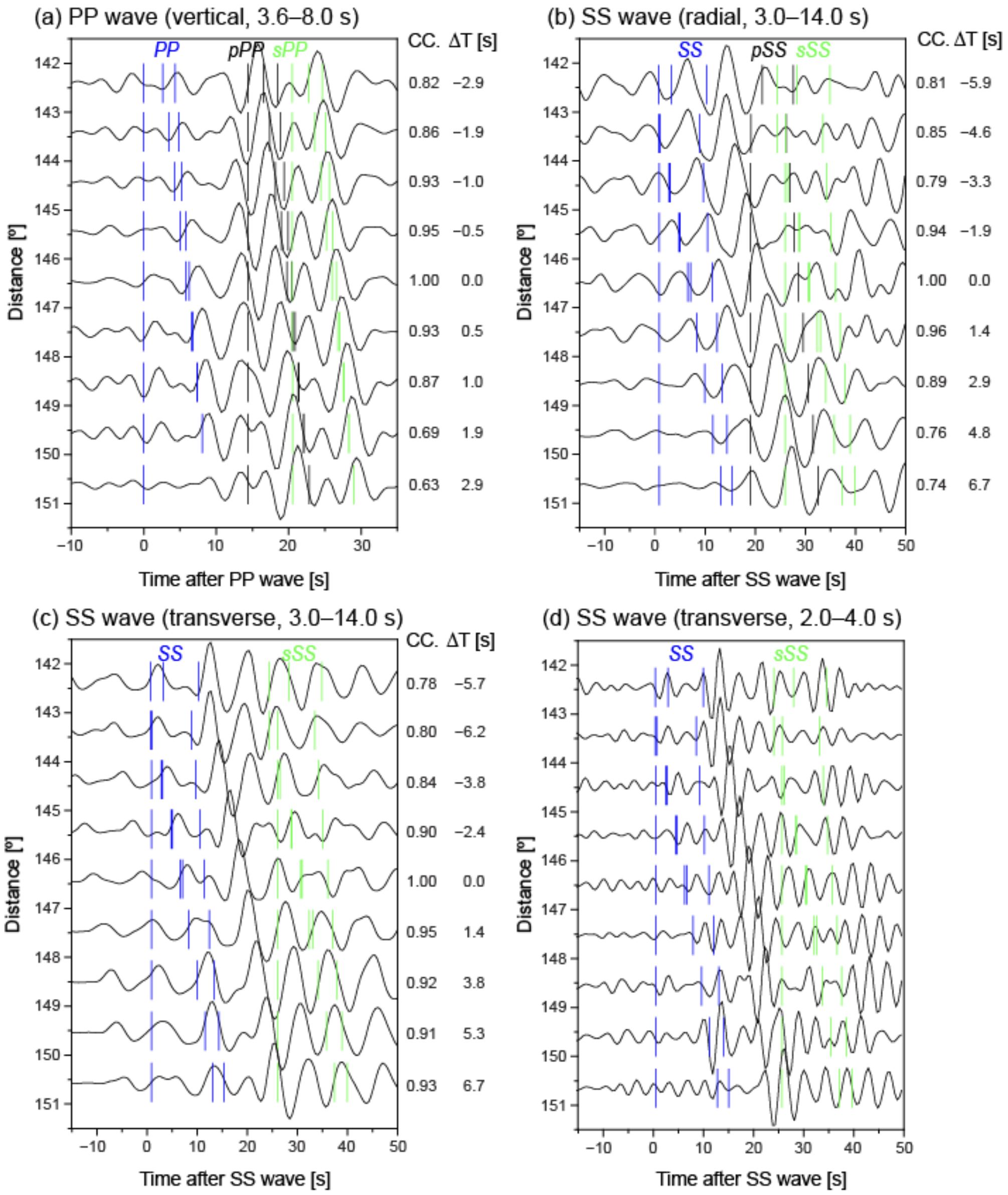

Figure Res. 12. Distance-dependent waveform variations of PP and SS phases. Synthetic seismograms are computed using the preferred focal mechanism solution (Fig. 2) at a fixed depth of 35 km and the interior velocity model of Khan et al. (2023). Waveforms are aligned on the predicted first arrivals of the PP or SS phases based on this velocity model. Vertical ticks indicate the theoretical arrival times of PP and SS phases (blue), as well as depth phases (pPP and pSS in grey; sPP and sSS in green). Multiple arrivals of PP, SS, or depth phases are present within a single trace; these are all predicted using the TauP travel-time toolkit (Crotwell et al., 1999), and all possible arrivals are displayed. To evaluate waveform stability with respect to epicentral distance, cross-correlation coefficients (CC) are calculated for each wavetrain relative to a reference waveform at 146.5°. The corresponding CC values and optimal time shifts are indicated on the right side of each panel. Apparent phase shifts and waveform

distortions with increasing distance demonstrate that distance variations introduce effects beyond a simple uniform time shift. This figure is now included as Supplementary Fig. 13 in the revised Supplementary Information.

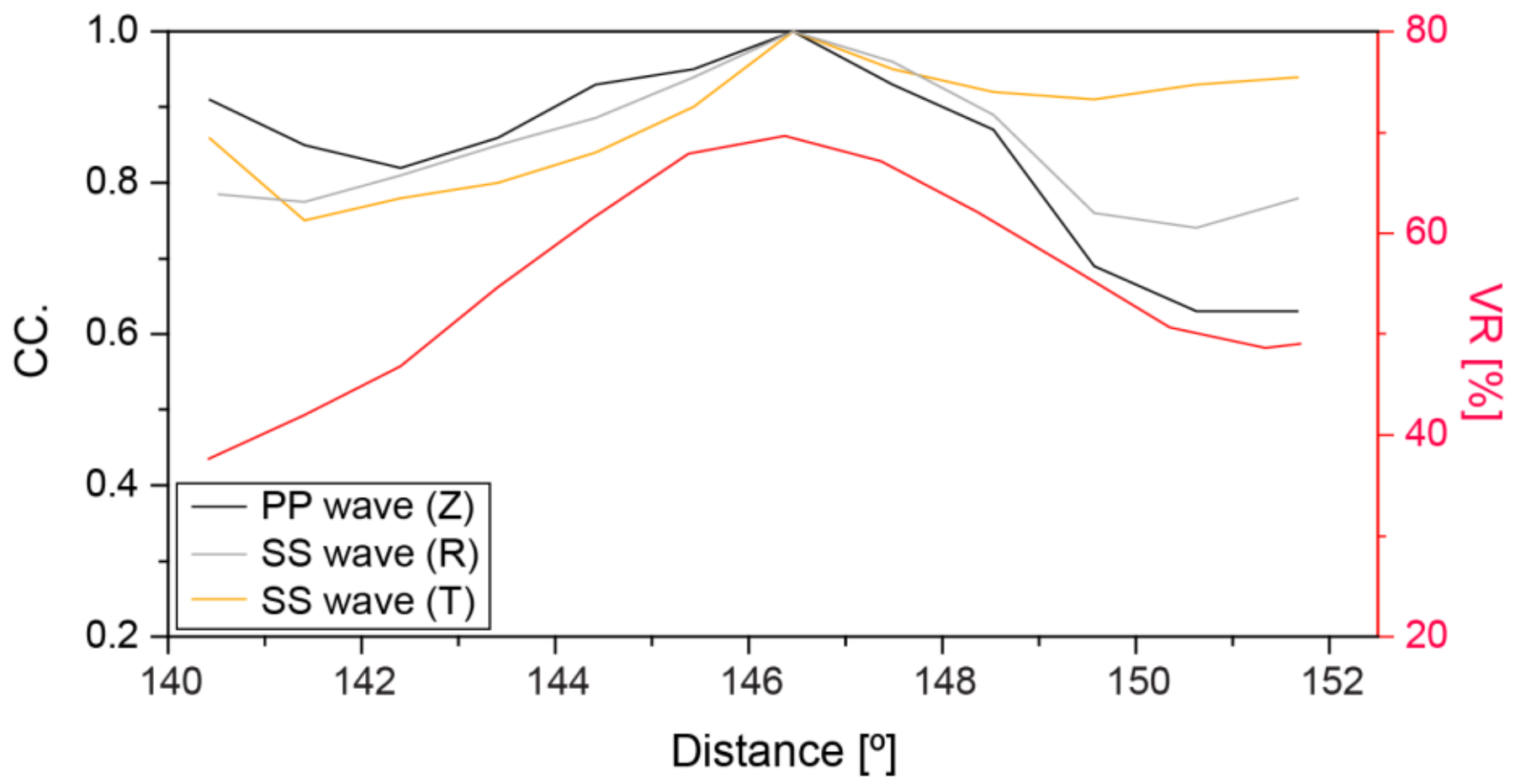

Figure Res. 13. Distance dependence of waveform similarity and waveform VR. Estimated CC values from the synthetic waveforms in Fig. Res. 12 and VR values from moment tensor inversion of the observed data are shown as a function of epicentral distance. This figure is included as Supplementary Fig. 14.

7. Lines 85–86: The cited ref. 45 investigated attenuation parameters within 0.05–0.09 Hz (Onodera et al., 2023), rather than "near 0.5 Hz" as stated in the text. The cited ref. 46 only includes marsquake data recorded before sol 478, meaning the S0976a event analyzed in this study is not part of that catalog (Clinton et al., 2021). Please update the citation to Ceylan et al. (2022).

12. We thank the reviewer for pointing this out. We have corrected the frequency range associated with ref. 45 and updated the citation as suggested.

The revised text now reads (**lines 93–95**):

"...which are derived from Martian teleseismic estimates at periods near 15 s[28,49] and are appropriate for modelling S0976a as a low-frequency event[50]."

Here, ref. 50 has been updated to Ceylan et al. (2022).

8. Lines 89–91: The authors should clarify the structural and geomechanical basis for using surface fault density as a direct proxy for the location of a deep-seated marsquake occurring at 20–40 km depth. Because a seismogenic depth of 20–40 km places the source firmly within the mid-to-lower crust,

localized surface fault traces may not directly map to the deep stress field or deep-seated fault geometries. The authors should provide a physical justification for this assumption or tone down the text to acknowledge the geometric separation between deep seismic slip and surface structural expressions.

13. Our use of surface fault density was intended as a first-order, regional weighting rather than as a direct proxy for the deep fault plane or local stress geometry. The structural rationale is that dense surface faulting in Valles Marineris marks the surface expression of long-lived crustal deformation zones associated with lithospheric extension, inherited weakness and cumulative strain localisation, as commonly observed in reactivated fault systems (e.g., Holdsworth et al., 2001) and as inferred for the Valles Marineris tectonic architecture (Andrews-Hanna, 2012). We have clarified and toned down the text accordingly.

The original text:

"Within western VM, the spatial density of previously mapped faults, used here as a proxy for regions prone to seismicity[42], provides an independent constraint on the location and points to a source near Ius Chasma (Methods; Fig. 2a). We note that this weighting is only approximate, but it is compatible with the inferred focal mechanism, as tectonic structures outside the canyon system more commonly include contractional features such as wrinkle ridges[47]."

has been revised to the following (**lines 107–114** in the revised manuscript):

"Within western VM, the spatial density of previously mapped faults, used here as a first-order proxy for regions prone to seismicity[48], provides an independent constraint on the location and points to a source near Ius Chasma (see "Methods"; Fig. 2a). In this region, mapped surface structures likely mark the surface expression of broader crustal deformation zones associated with long-term lithospheric extension and strain localisation[20]. We note that this weighting is approximate, as fault density informs the regional distribution of deformation rather than directly constraining the geometry of deep seismic slip."

We also explicitly note that the weighting is approximate and that fault density informs the regional distribution of deformation in the Methods (**lines 451–452**).

9. Lines 101–102: On the radial component, the synthetic SS waveform generated by the force-based source model exhibits an amplitude similar to the observed data (Fig. 2b).

14. We have revised the text to clarify this point and to more accurately describe the limitation of the force-based source model.

The revised text now reads (**lines 126–127** in the revised manuscript):

"In particular, force-based source models cannot account for the strong horizontal ground motion observed in the data."

10. Line 144: The citation for ref. 52 should be moved forward to where the CTX is first introduced in the manuscript (i.e., Line 108).

15. We appreciate the reviewer's comment. We have moved the citation for Ref. 52 to the first mention of CTX in the manuscript (**line 133** of the revised manuscript).

11. Fig. 3: The abbreviation of CTX should be defined in the main text where it is first mentioned at Line 108.

16. The abbreviation CTX has been defined at its first mention in the main text (**line 133** of the revised manuscript).

12. Fig. 3: "a boulder-fall site" or "boulder-fall sites"?

17. Thank you for pointing this out. Since multiple locations are referred to in Fig. 4 (originally Fig. 3), we changed "a boulder-fall site" to "boulder-fall sites" throughout the figure and caption for consistency and accuracy.

13. Fig. 3a: The authors should clarify what the circle outlined in black located at approximately 78.4°W/6°S represents, as its definition is missing from both the figure legend and the caption.

18. The black-outlined circle was an inadvertent remnant from an earlier version of the figure, in which we considered displaying the best-fitting focal-mechanism location. As this feature is not discussed in the manuscript and is not required for the final presentation, we have removed the circle from Fig. 4a to avoid confusion.

14. Line 242: I don't think ref. 65 (Knapmeyer-Endrun et al., 2021) is an appropriate reference to support the statement regarding "the clustering of marsquakes within localized deformation zones". The cited study focused fundamentally on constraining the crustal thickness and layering beneath the InSight landing site. It did not investigate marsquake epicentral clustering, spatial seismicity distribution, or

active deformation zones. The authors should replace this reference with literature that addresses seismicity on Mars, such as the InSight catalog papers or studies explicitly mapping event distributions.

19. We have removed this citation from the manuscript, and to better support the statement, we now cite studies that directly document the concentration of Martian seismicity within tectonically active regions, particularly Cerberus Fossae (Stähler et al., 2022). We have also revised the sentence to separately reference independent lines of evidence for a mechanically strong lithosphere, including large elastic thickness, gravity–topography relationships indicative of long-wavelength flexural support, and the localisation of marsquake activity within a limited number of tectonically active regions.

We revised the corresponding sentence as follows (**lines 269–271**):

"...supported by independent constraints, including large elastic thickness (Solomon & Head, 1990), gravity–topography relationships indicative of flexural support (McGovern et al., 2002), and the clustering of marsquakes within localised deformation zones (Stähler et al., 2022)."

15. Lines 268–269: The authors should explicitly label the main PP and SS waves, as well as their depth phases, directly on the displayed waveforms. Given that the synthetic waveforms achieve a highly satisfactory fit to the data in Fig. 2b, these phase arrivals can be easily identified and cross-verified using the synthetics. For example, are the large-amplitude arrivals occurring ~30 s after PP and SS the depth phases? If so, explicitly identifying them will significantly improve the readability of the figures and allow readers to clearly trace the key observational constraints.

20. We have indicated the main PP and SS waves and their depth phases on Fig. 2b together with predicted arrival times. The main and depth phases are identified based on Fig. Res. 12, where we modelled synthetic waveforms at different distances. We also clarify that the zero time in Fig. 2b is based on picking of Li et al. (2022), which is now indicated in the figure caption.

16. Line 301: Based on the parameter sampling described by the authors, the calculation at each location should be 60 * 60 * 60 * 8 * 50 (corresponding to the number of searched strike, dip, rake, scalar moment, and source depth increments, respectively). This yields 8.64 × 107 total trials, rather than the "5 × 107" stated in the text. Please double-check accordingly.

21. We thank the reviewer for catching this. Rather than retaining the previous approximate trial-count statement, we now describe the full grid-search ensemble and define the acceptable-solution ensemble explicitly. For each candidate source location, depth and scalar moment, we calculated VR for all strike–dip–rake combinations and retained solutions with VR > 0.9 × VRmax. The resulting ensemble is summarised in Supplementary Table 1.

17. Lines 345–361: I suggest the authors move the section titled "Amplitude-based source analysis" into the Supplementary Information. There appears to be no substantive discussion or corresponding statement interpreting the actual results of this method within the main text.

22. We appreciate the reviewer's suggestion. We have retained the amplitude-based analysis in the main manuscript and added a brief discussion of its results and limitations. The new text clarifies that, although the amplitude-ratio method suffers from the expected non-uniqueness of single-station observations, it independently recovers a strike-slip mechanism consistent with the preferred waveform-inversion solution.

The following short discussion has been added in the revised manuscript (**lines 102–104**):

"An independent amplitude-ratio method (Sita & van der Lee, 2022) yields a comparable strike-slip mechanism, although these solutions retain the expected fault-plane ambiguity associated with single-station amplitude methods (Supplementary Fig. 16)."

18. Supplementary Fig. 1: Why the displayed misfit values are so small (on the order of ~1e-12)? Are they normalized to the maximum misfit among all grid-search trials? Regarding the uncertainty analysis requested in the first major comment, could the authors provide the acceptable ranges of the resolved source parameters instead of presenting only a single best-fitting solution? Visualizing how the parameter space behaves near the minimum is critical for assessing potential trade-offs and evaluating the true robustness of the inversion.

23. The misfit values shown in Supplementary Fig. 1 are the raw L_2 waveform misfit values computed by MTUQ (eq. 7 in Thurin et al., 2025) and are not normalised by the maximum misfit among the grid-search trials. In MTUQ, the misfit is defined as the summed squared residuals between observed and synthetic waveforms, so its absolute magnitude depends on the waveform amplitudes and data units used in the inversion.

Following the reviewer's suggestion, we additionally quantified the uncertainty of the focal mechanism solution by examining the parameter space within the acceptable-solution ensemble, defined by VR > 0.9 × VRmax (see Response #5). The corresponding acceptable ranges of strike, dip, and rake are now provided in the revised Supplementary Information. These results allow a more direct assessment of parameter trade-offs and inversion robustness.

We added the following sentence to the caption of Supplementary Fig. 1:

“The misfit corresponds to the L_2 norm of waveform residuals, defined as the summed squared differences between observed and synthetic waveforms (Thurin et al., 2025).”

19. Supplementary Figs. 6–9: The x-axis label in the right panels should be “Back azimuth” instead of “Azimuth”

24. Thanks for pointing this out. The x-axis label has been corrected from “Azimuth” to “Back azimuth” in Supplementary Figs. 6–9.

20. Supplementary Fig. 10: Are the waveforms displayed here the best-fitting solutions obtained for each individual model case? If so, why do none of them match the synthetics shown in Fig. 2b, which the authors label as the “best-fitting waveform comparisons”? For example, the negative arrival at ~30 s after PP (a likely depth phase) is quite well fitted in Fig. 2b, yet it shows a clear deficit and is poorly reproduced here when using the Khan and the Samuel models.

25. The waveforms shown in the original figure corresponded to the best-fitting solutions obtained for each velocity model under the source-location constraints used in the depth-sensitivity analysis. In that experiment, the back azimuth and depth were searched, while the epicentral distance was fixed independently for each velocity model based on the PP–SS differential travel time.

In contrast, the waveform comparisons shown in Fig. 2b correspond to the preferred inversion presented in the main text, for which the source location parameters were not constrained in the same way and were optimised through waveform fitting. To avoid confusion, we have replaced the original Supplementary figure (now Supplementary Fig. 12) with new waveform comparisons obtained using the same back azimuth as the preferred solution in the main text (93°). For each velocity model, the epicentral distance and source depth were independently optimised while keeping the back azimuth fixed, and the resulting best-fitting solutions are now shown.

Regarding the negative arrival approximately 30 s after PP, we agree that this phase is more clearly reproduced by the deeper-source solutions, as discussed in our response to Comments #6 and #15. However, the waveform inversion minimises the overall misfit across both the PP and SS wavetrains. As a result, solutions that provide a better fit to the complete waveform dataset may be favoured even if they reproduce this particular depth-sensitive arrival less accurately. This trade-off explains why the negative arrival is less well matched in some of the Supplementary Fig. 12 solutions despite their lower overall waveform misfit.

21. Supplementary Fig. 13: This figure clearly shows that polarization information is lacking below 0.2 Hz. Consequently, the 0.08–0.2 Hz frequency band used for the waveform inversion may be operating in a regime where particle motions are poorly constrained due to low signal-to-noise ratios or environmental ambient noise. The authors should explain how they accounted for this observational deficit or explicitly discuss how the lack of low-frequency polarization impacts the overall uncertainty and stability of their focal mechanism solution.

26. We thank the reviewer for this important observation. To address this concern, we repeated the waveform inversions using an extended frequency band (3.6–8.0 s for PP and 3.0–14.0 s for SS) that incorporates more source energy. Following the reviewer's suggestion, we have replaced the main-text results and associated supplementary results with those obtained from the extended frequency-band inversion (see Response #2). We have also added a discussion of the frequency-band dependence and its implications for inversion uncertainty in the revised manuscript (**lines 87–90**).

References

1. Andrews-Hanna, J. C. The formation of Valles Marineris: 1. Tectonic architecture and the relative roles of extension and subsidence. J. Geophys. Res. Planets 117, E06002 (2012).
2. Brinkman, N. et al. First focal mechanisms of marsquakes. J. Geophys. Res. Planets 126, e2020JE006546 (2021).
3. Ceylan, S. et al. The marsquake catalogue from InSight, sols 0–1011. Phys. Earth Planet. Inter. 333, 106943 (2022).
4. Ceylan, S. et al. Mapping the seismicity of Mars with InSight. J. Geophys. Res. Planets 128, e2023JE007826 (2023).
5. Crotwell, H. P., Owens, T. J. & Ritsema, J. The TauP Toolkit: Flexible seismic travel-time and ray-path utilities. Seismol. Res. Lett. 70, 154–160 (1999).
6. Holdsworth, R. E., Stewart, M., Imber, J. & Strachan, R. A. The structure and rheological evolution of reactivated continental fault zones: A review and case study. In Continental Reactivation and Reworking (eds. Miller, J. A., Holdsworth, R. E., Buick, I. S. & Hand, M.) Geol. Soc. Lond. Spec. Publ. 184, 115–137 (2001).
7. Horleston, A. C. et al. The far side of Mars: Two distant marsquakes detected by InSight. Seism. Rec. 2, 88–99 (2022).
8. InSight Marsquake Service. Mars Seismic Catalogue, InSight Mission; V14 2023- 04-01 (2023). ETHZ, IPGP, JPL, ICL, Univ. Bristol. https://doi.org/10.12686/a21
9. Irving, J. C. et al. First observations of core-transiting seismic phases on Mars. Proc. Natl. Acad. Sci. USA 120, e2217090120 (2023).
10. Jacob, A. et al. Seismic sources of InSight marsquakes and seismotectonic context of Elysium Planitia, Mars. Tectonophysics 837, 229434 (2022).
11. Jiménez-Díaz, A., Egea-Gonzalez, I., Parro, L. M., Tasaka, M. & Ruiz, J. The thermal structure and mechanical behavior of the Martian lithosphere. Icarus 353, 113635 (2021).
12. Kaverina, A. N., Lander, A. V. & Prozorov, A. G. Global CMT distribution and its relation to earthquake-source geometry and tectonic origin. Geophys. J. Int. 125, 249–265 (1996).
13. Khan, A. et al. Upper mantle structure of Mars from InSight seismic data. Science 373, 434–438 (2021).
14. Khan, A. et al. Evidence for a liquid silicate layer atop the Martian core. Nature 622, 718–723 (2023).
15. Kim, D. et al. Global crustal thickness revealed by surface waves orbiting Mars. Geophys. Res. Lett. 50, e2023GL103482 (2023).
16. Knapmeyer-Endrun, B. et al. Thickness and structure of the Martian crust from InSight seismic data. Science 373, 438–443 (2021).

17. Li, J. et al. Constraints on the Martian crust away from the InSight landing site. Nat. Commun. 13, 7950 (2022).
18. Maguire, R. et al. Focal mechanism determination of event S1222a and implications for tectonics near the dichotomy boundary in southern Elysium Planitia, Mars. J. Geophys. Res. Planets 128, e2023JE007793 (2023).
19. Malytskyy, D. et al. High- and low-frequency waveform analysis the marsquake of Sol 1222: Focal mechanism, centroid moment tensor inversion and source time function. Earth Space Sci. 11, e2023EA003272 (2024).
20. McGovern, P. J. et al. Localized gravity/topography admittance and correlation spectra on Mars: Implications for regional and global evolution. J. Geophys. Res. Planets 107, 5115 (2002).
21. Samuel, H. et al. Geophysical evidence for an enriched molten silicate layer above Mars's core. Nature 622, 712–717 (2023).
22. Sita, M. & van der Lee, S. Potential volcano-tectonic origins and faulting mechanisms of three low-frequency marsquakes detected by a single InSight seismometer. J. Geophys. Res. Planets 127, e2022JE007309 (2022).
23. Solomon, S. C. & Head, J. W. Heterogeneities in the thickness of the elastic lithosphere of Mars: Constraints on heat flow and internal dynamics. J. Geophys. Res. Solid Earth 95, 11073–11083 (1990).
24. Song, J.-H. et al. New magnitude scaling and seismic source characterisation for marsquakes and impacts. Earth Space Sci. Open Archive. https://doi.org/10.22541/essoar.15005266/v1 (2026).
25. Stähler, S. C. et al. Seismic detection of the Martian core. Science 373, 443–448 (2021).
26. Stähler, S. C. et al. Tectonics of Cerberus Fossae unveiled by marsquakes. Nat. Astron. 6, 1376–1386 (2022).
27. Thurin, J. et al. MTUQ: a framework for estimating moment tensors, point forces, and their uncertainties. Geophys. J. Int. 241, 1373–1390 (2025).

From Reviewer 2:

Assessing whether a planet is geodynamically active today represents one major challenge of planetary science. Before NASA's InSight mission landed on Mars, whether the red planet is geodynamically active today remained unclear, and relied on observational evidence of geologically young lava flows. The InSight SEIS instrument recorded hundreds of marsquakes demonstrating the planet to be (at least regionally) tectonically active, and with the locus of seismic activity being related to the large Cerberus Fossae fissure system located in Elysium Planitia. Surprisingly, only a handful of marsquakes have been detected outside of Elysium Planitia, with a single one being located in the western hemisphere. Assessing the origin of this western hemisphere quake, and particularly whether it is related to surface (impact, landslide) or interior (geodynamic) processes would provide important information on Mars' tectonic activity.

In this work, the authors perform a detailed seismic investigation of the single western hemisphere marsquake recorded by InSight. In agreement with previous work, the quake was found to originate from the large Valles Marineris (VM) fissure system. Synthetic and inversion models are presented to characterize the seismic source of the quake, which is found to be consistent with a strike-slip reactivation of pre-existing faults. Surface sources including impacts and landslides are demonstrated to be inconsistent with the data. The authors then suggest that the quake is related to lithospheric stresses generated early in Mars' evolution, and argue that such stresses can be preserved in the crust for billions of years.

In general, I find the manuscript well written. Importantly, I note that I am not a seismologist and have little expertise with analyses of InSight-detected marsquakes. Although the inversion methodology and marsquake-specific results seemed sound, I emphasize that I don't have the expertise to have a detailed critical outlook on these aspects. However, and as further described below, I have some major concerns regarding the (often unclear) authors' interpretations of the source of stresses. In addition, I believe the authors largely inflate the conclusion of their work, which "indicate that lithospheric stresses generated early in planetary evolution can persist over billions of years on a stagnant-lid planet". Across planetary bodies, faults are known to reactivate over billions of year and paleo-stress fields are also known to affect fault patterns. One important aspect of the work that was not sufficiently explored is related to the origin of the present-day stress that triggered the quake. A very simple figure and discussion explain that Tharsis could be the source, but further consideration (uplift? subsidence?) is lacking (see below). For these reasons, I recommend the paper be returned for major revisions. The review is organized as follows: first, I list my major concerns and then provide minor line-numbered comments. All must be addressed thoroughly.

1. We thank the reviewer for their thoughtful and constructive comments on our manuscript. We are pleased that the reviewer found the manuscript well written and considered the marsquake-specific seismic analysis and inversion methodology to be sound. On reflection, we agree that the reviewer's two main concerns helped us to substantially improve the framing of the tectonic interpretation.

In particular, we recognise that some statements in the original manuscript concerning "early lithospheric stresses" could have been read as implying that ancient stresses alone directly drive present-day seismicity. We also agree that the possible origin of the contemporary stress field responsible for S0976a was not discussed in sufficient depth.

We have therefore revised the manuscript to address these issues more clearly. The revised discussion now distinguishes between (1) inherited tectonic structures and fault fabrics, (2) residual or near-critical lithospheric stress states that may influence how close faults are to failure, and (3) contemporary stress fields that are required to reactivate present-day seismic slip. In doing so, we no longer unintentionally imply that inherited lithospheric stresses alone drive present-day tectonics. Instead, the revised text makes clear that ancient tectonic fabrics and heterogeneous lithospheric stress states may persist in a cooling stagnant-lid lithosphere and help localise later seismic failure when acted upon by subsequent or ongoing stress sources.

Following the reviewer's suggestion, we have also expanded the discussion of possible sources of the present-day stress field, including the role of Tharsis-related loading and lithospheric flexure, and have clarified the distinction between long-lived tectonic inheritance and active triggering mechanisms. We have further added discussion of relevant previous work on long-lived fault reactivation and inherited tectonic fabrics on Mars and Mercury. These changes are highlighted in **blue** in the revised manuscript and Supplementary Information, and the corresponding line numbers and figure references are provided throughout this response where relevant. In addition, we have submitted a tracked-changes version of the Article, comparing the originally submitted manuscript with the revised version, as a related manuscript file. We believe these revisions provide a more physically sound and conceptually coherent interpretation of the relationship between ancient tectonic structures, present-day stress, and the source mechanism of S0976a.

Major concerns:

1. There is a critical unclear aspect of the work and a potential important misconception regarding early stresses, such as in statement like these (found throughout the text):

A. Abstract: "stresses generated during early planetary evolution remain capable of driving present-day tectonics has not been directly tested": This is very unclear. Stress is the measure of an external force

acting over an area. If the external force is still ongoing, stress can build up leading to tectonic activity (providing the stresses exceeded a critical threshold). One cannot "generate" a stress early, stop it, and then get some tectonic activity (as suggested by this sentence). Maybe the authors mean "initiated" and not "generated"? The crust can however preserve a "memory" of paleostresses that can be reactivated by a new active stress period. But this doesn't mean the new active stress is related to the early stress. Note also that previous work, for example Klimczak et al. (2025) [https://www.sciencedirect.com/science/article/abs/pii/S0012821X2500130X] has shown indicating that Mercury's global contraction has reactivated a tectonic fabric inherited from past stress events (e.g., basin formation). Re-activation of faults has also been extensively covered in the literature (e.g., Woodley et al., (2024); 10.1029/2023GL107757; Andrews-Hanna et al. (2008); https://doi.org/10.1029/2007JE002980. and many others).

2. We agree with the reviewer that our original wording was misleading. We did not mean to imply that stresses generated early in Mars' history directly drive present-day seismicity, but rather that ancient tectonic structures may persist in the lithosphere and localise failure when subjected to a contemporary stress field. We have therefore revised the Abstract.

The original sentence:

"Whether stresses generated during early planetary evolution remain capable of driving present-day tectonics…"

has been replaced with:

"Whether the ancient tectonic fabrics can localise present-day seismic failure under contemporary stress fields remains poorly tested by direct geophysical observations."

As discussed in our response to the general comment above (Response #1), we have revised the Introduction (**lines 5–17**) to make this distinction clear. We have also added discussion of fault reactivation in the context of the previous studies suggested by the reviewer (**lines 216–225, 268–269**).

B. Line 9 (also Line 11): "but the alternative hypothesis is that stresses generated early in planetary evolution are stored within the lithosphere and released episodically": As noted above, this is also unclear. One cannot release stresses episodically without a driving force. Early stresses would be stored in the crust up to some failure threshold (e.g., Byerlee for friction) and cannot be released. However, any subsequent stress on a crust that is already at a failure threshold would generally lead to strain.

3. We agree with the reviewer that this statement was similarly misleading. We have revised this part of the Introduction.

The original sentence:

"...stresses generated early in planetary evolution are stored within the lithosphere and released episodically."

has been replaced by a revised paragraph that now states (**lines 10–12** in the revised manuscript):

"At the same time, ancient tectonic structures and heterogeneous, near-critical lithospheric stress states may remain preserved, leaving inherited faults susceptible to reactivation by subsequent or ongoing stress sources."

C. Line 46: "likely driven by inherited lithospheric stresses" Inherited lithospheric stresses cannot drive tectonic activity. It can affect the local activity, providing another external acting stress exists.

4. We thank the reviewer for the careful reading. We have revised this statement.

The original sentence:

"Together, these observations provide converging geophysical and geological evidence that VM remains tectonically active, likely driven by inherited lithospheric stresses."

has been replaced with (**lines 49–52** in the revised manuscript):

"Together, these observations provide converging geophysical and geological evidence that VM remains tectonically active today, with seismicity localised by inherited fault systems reactivated under contemporary lithospheric stress fields."

2. One important aspect of the work relates to the secondary component of the deformation. Strike slip is interpreted to be dominant, but the authors suggest (Line 68) some extension, indicating transtension. I think this aspect needs to be discussed further. A compressional component would generally be consistent with a globally cooling Mars, where stresses are stored passively over time. Some extension would instead require a more exotic stress source. Depending on the rigidity of the lithosphere, extension at VM could be associated with ongoing Tharsis subsidence (downward directed load). Interestingly, it could also be related to uplift in the Tharsis plateau (upward directed load), which could be associated with some ongoing magmatic activity (a plume?). I think this aspect would be very important and interesting to dive in. I see that the authors provide a very simple story of Tharsis loading (Figure 4), but more quantitative estimates are critically lacking.

5. We have clarified in the revised manuscript that the focal mechanism solution for S0976a is characterised primarily by horizontal compression, consistent with dominantly strike-slip motion. The

extensional component is secondary and is inferred mainly from the orientation of the T axis in the focal mechanism solution. We agree, however, that interpretation of this component benefits from a more detailed discussion of the different stress sources that could produce the observed mechanism.

We have therefore expanded the discussion (**lines 216–225**) to place our interpretation in the context of previous geodynamic studies. These additions provide a more quantitative, model-informed discussion of how different stress contributions may account for both the dominant strike-slip component and the secondary extensional component of the S0976a mechanism. In the revised manuscript, we discuss why global contraction alone may be insufficient to explain the possible extensional component, although it may still contribute to strike-slip reactivation on favourably oriented inherited faults. We now cite Andrews-Hanna et al. (2008), who showed that combined Tharsis loading and planetary contraction can favour strike-slip faulting on Mars. This provides a plausible explanation in which contractional or compressional stresses may reactivate inherited faults in strike-slip motion.

For the possible transtensional component at Valles Marineris, we now discuss additional stress sources (**lines 226–241**), including uplift or dynamic support of the Tharsis plateau, which could generate extensional stresses depending on the geometry and rigidity of the lithosphere (Andrews-Hanna, 2012b). We also discuss the possibility of plume-related or buoyant mantle support beneath Tharsis, following Root et al. (2026), as well as present-day convective stresses, which may be non-negligible according to the thermal evolution models of Plesa et al. (2018).

The revised schematic (now Fig. 5 in the revised manuscript) shows long-term planetary contraction and Tharsis-related loading as candidate regional stresses, while indicating subordinate local transtension near VM. Dynamic support, flexure and subsidence are discussed in the Discussion as possible contributors to the local transtensional component, rather than shown as a uniquely resolved stress source.

3. I am not sure I understand the title of the paper. Crustal strain at VM is observed and it must be persistent. Activity on the other hand is important to note.

6. Agreed. We modified the title to:

“Active crustal straining and fault reactivation at Valles Marineris.”

to emphasise the present-day reactivation of an inherited fault system at Valles Marineris.

Minor concerns:

Abstract: “wane as lithospheres cool and thicken”: This is not always true, as planets cool they also record substantial contraction (from shrinking) that will cause tectonic activity. Abstract: “stresses generated early in planetary evolution can persist over billions of years on a stagnant-lid planet. Our results suggest that tectonic activity on rocky planets without plate tectonics may persist longer than previously assumed, reflecting their contemporary thermal states.”: See my comment above regarding the fact that this work “suggests” [it has been shown already in the past!].

7. We have revised the Abstract to avoid implying that cooling and lithospheric thickening necessarily lead to monotonic tectonic decline. We have also removed the statement that “lithospheric stresses generated early in planetary evolution can persist over billions of years” and replaced it with wording that emphasises inherited fault systems, contemporary stress, and localised reactivation. We further moderated the final sentence to indicate direct seismic evidence for present-day fault reactivation at Valles Marineris. The revised Abstract now reads as follows:

“Tectonic deformation on stagnant-lid planets reflects the interaction between long-lived lithospheric structures and evolving stress fields. Whether the ancient tectonic fabrics can localise present-day seismic failure under contemporary stress fields remains poorly tested by direct geophysical observations. Here we show that the farside seismic event, S0976a, detected by InSight in the western hemisphere is a marsquake that occurred in Valles Marineris and involved strike-slip faulting in the deep crust. The stress orientation inferred from the focal mechanism is consistent with reactivation of pre-existing fault systems under a present-day stress field shaped primarily by Tharsis-related loading and long-term planetary cooling, with a subordinate transtensional component. These observations provide direct seismic evidence that inherited fault systems on Mars can remain mechanically favourable for reactivation over billion-year timescales, suggesting that slip on deeply rooted faults remains a key mechanism of long-term strain release in a cooling single-lid planet.”

Line 5: “activity typically decreases as the lithosphere cools and thickens” Please see the first minor comment above.

8. We agree and have revised this sentence. The Introduction (**lines 5–8** in the revised manuscript) now states that stagnant-lid planets lack global lithospheric recycling and that stresses can arise from volcanic loading, global contraction, lithospheric flexure, mantle convection, and magmatic activity.

Line 7: “deformation continues well beyond the main phase of planetary cooling” same as above

9. We revised the corresponding phrase (**lines 8–10** in the revised manuscript), which now states that geologically young faults and seismic activity on Mars indicate that deformation can continue long after

early planetary evolution, without implying that deformation is unexpected solely because cooling has proceeded.

Line 22: “primary” -> “primordial”?

10. We have revised the wording to avoid ambiguity (**line 27**). The original phrase “primary tectonic structures” has been replaced with “primordial tectonic structures”, which better conveys that these structures are associated with the early stages of Valles Marineris deformation.

Line 65: Here the authors discuss and provide only the best-fit. But looking at Fig S1, the misfit seems rather flat for some parameters (e.g. dip going to 60°? Strike to 160? Slip of ±20°?).

11. We modified the text to describe the preferred mechanism in terms of the acceptable-solution ensemble rather than only the single best-fitting solution, with the aim of providing objective statistical ranges for the acceptable strike, dip, and rake values (**lines 74–77**). Specifically, we defined acceptable focal-mechanism solutions as those with variance reduction (VR) values greater than 0.9 × VRmax, where VRmax is the maximum variance reduction obtained from the full grid search (Please see our Response #5 to Reviewer 1 for the detailed statistical analysis and a description of how the acceptable-solution ensemble was defined and constructed.). We then used this acceptable-solution ensemble to quantify the distributions of strike, dip, and rake. We also indicate the strike ranges of the two nodal-plane populations in Fig. 4a and include the distribution of acceptable source types using the Kaverina diagram (Kaverina et al., 1996) in Fig. 3f.

Fig S2: It is great that the best-fit reproduce the data. But what about the sensitivity of the inversion to specific parameters? It would be great to show a Figure similar to Fig S1 for this test case.

12. We have now added a plot (now in Fig. S11c) showing that acceptable solutions remain concentrated in the strike-slip domain, supporting the robustness of the inferred source type.

Line 94: Small graben above wrinkle ridges have been observed on Mercury, indicating that extension can also occur at wrinkle ridges [https://www.nature.com/articles/s41561-023-01281-5].

13. We have clarified that the fault-density weighting is used as a first-order spatial proxy rather than a direct kinematic constraint. The preferred source region remains more consistent with the inferred

mechanism because it coincides with mapped graben, normal-fault, and oblique fabrics near Ius Chasma, whereas structures outside the canyon system are more commonly contractional.

The sentence around line 94 of the original manuscript,

"…it is compatible with the inferred focal mechanism, as tectonic structures outside the canyon system more commonly include contractional features such as wrinkle ridges[47]"

has been revised to (**lines 110–112** of the revised manuscript):

"In this region, mapped surface structures likely mark the surface expression of broader crustal deformation zones associated with long-term lithospheric extension and strain localisation[20]."

Here, ref. 20 refers to Man et al., 2023.

Line 188: Please note that there is a debate regarding the origin of wrinkle ridges. For example, work by Montési & Zuber (2003; https://agupubs.onlinelibrary.wiley.com/doi/10.1029/2002JE001974) argue for deep-seated wrinkle ridges.

14. We thank the reviewer for this point. We have revised the text to cite Montési and Zuber (2003) and to distinguish shallow-detachment models from deeper-seated interpretations. The revised manuscript now treats the focal depth as one line of evidence, considered together with the oblique strike-slip mechanism and the mapped structural fabrics in VM, in support of reactivation of pre-existing structures under the present-day stress field.

The original sentence:

"The focal depth of S0976a (20–40 km) further implies that the event is unlikely to have originated on the detachment systems of wrinkle ridges, which commonly occur at shallower depths (<10 km)[56,57], and instead points to deep-seated deformation within a mechanically strong lithosphere.",

has been revised to the following (**lines 203–205** of the revised manuscript):

"The focal depths of S0976a, ~20–40 km, are deeper than the inferred shallow wrinkle-ridge detachments, <10 km[62,63], although deeper-seated wrinkle-ridge faulting has also been proposed[64]."

Here, ref. 64 refers to Montési & Zuber (2003).

Line 200: Ref 61 must be a mis-citation here. Maybe Plesa et al. (2018) [https://agupubs.onlinelibrary.wiley.com/doi/full/10.1002/2017GL076124] would be more adequate here. Actually, present-day mantle convection does lead to non-negligible convective stresses. I also note that recent work by Root et al. 2026 [https://agupubs.onlinelibrary.wiley.com/doi/full/10.1029/2024JE008765] argues for the existence of a present-day large plume below Tharsis, which would generate surface uplift and stresses.

15. We have removed the original statement and replaced the previous citation with Plesa et al. (2018), which is more appropriate for discussing present-day Martian seismicity and stresses associated with mantle convection, planetary cooling, and contraction (**lines 216–217** in the revised manuscript**)**. We also added discussion (**lines 226–232**) of Root et al. (2026), including the possibility that Tharsis uplift or dynamic support associated with buoyant mantle material or plume-like upwelling could contribute to extensional or transtensional stresses at Valles Marineris.

Line 203: I disagree here. If there is a pre-existing tectonic fabric, global contraction could reactivate faults, leading to strike-slip.

16. We have revised this interpretation to clarify that global contraction may provide a planet-wide compressive background stress and that, when superposed on Tharsis-induced loading stresses or acting on favourably oriented inherited faults, it can contribute to strike-slip reactivation. We cite Andrews-Hanna et al. (2008) in support of this interpretation (**lines 217–225**).

Line 212: The link between faulting and strength is unclear.

17. We have revised the relevant sentence to clarify that brittle faulting is consistent with a cold, mechanically strong lithosphere capable of supporting differential stresses at middle-to-lower crustal depths, while pre-existing fault zones provide mechanically weak surfaces that focus later seismic deformation under slowly evolving stress fields. This revised wording avoids implying a simple one-to-one link between the occurrence of faulting and bulk lithospheric strength.

The original sentence:

“The persistence of brittle faulting beneath VM implies that the Martian lithosphere retains sufficient strength to preserve long-lived zones of weakness and sustain differential stresses over geological timescales.”

has been revised to (**lines 242–245**):

“The occurrence of brittle faulting beneath VM is consistent with a cold, mechanically strong Martian lithosphere capable of supporting differential stresses at middle-to-lower crustal depths[74], while pre-existing fault zones provide mechanically weak surfaces that focus later seismic deformation under slowly evolving lithospheric stress fields.”

Here, ref. 74 refers to Jiménez-Díaz et al. (2021).

References

1. Andrews-Hanna, J. C. et al. Strike-slip faults on Mars: Observations and implications for global tectonics and geodynamics. J. Geophys. Res. Planets 113, E08002 (2008).
2. Andrews-Hanna, J. C. The formation of Valles Marineris: 2. Stress focusing along the buried dichotomy boundary. J. Geophys. Res. Planets 117, E04009 (2012).
3. Jiménez-Díaz, A., Egea-Gonzalez, I., Parro, L. M., Tasaka, M. & Ruiz, J. The thermal structure and mechanical behavior of the Martian lithosphere. Icarus 353, 113635 (2021).
4. Kaverina, A. N., Lander, A. V. & Prozorov, A. G. Global creepex distribution and its relation to earthquake-source geometry and tectonic origin. Geophys. J. Int. 125, 249–265 (1996).
5. Klimczak, C., Crane, K. T. & Byrne, P. K. Mercury has multiple, superposed global tectonic patterns. Earth Planet. Sci. Lett. 658, 119331 (2025).
6. Man, B. et al. Widespread small grabens consistent with recent tectonism on Mercury. Nat. Geosci. 16, 856–862 (2023).
7. Montési, L. G. & Zuber, M. T. Clues to the lithospheric structure of Mars from wrinkle ridge sets and localization instability. J. Geophys. Res. Planets 108, E06006 (2003).
8. Plesa, A. C. et al. Present-day Mars' seismicity predicted from 3-D thermal evolution models of interior dynamics. Geophys. Res. Lett. 45, 2580–2589 (2018).
9. Root, B. et al. Describing the global gravity field of Mars with lithospheric flexure and deep mantle flow. J. Geophys. Res. Planets 131, e2024JE008765 (2026).
10. Watters, T. R. et al. Shallow seismic activity and young thrust faults on the Moon. Nat. Geosci. 12, 411–417 (2019).
11. Woodley, S. Z. et al. Repeated and long-lasting fault activation on Amazonian Mars as demonstrated by tectonically induced landslides. Geophys. Res. Lett. 51, e2023GL107757 (2024).

From Reviewer 1:

Review comments on "Active crustal straining and fault reactivation at Valles Marineris" by Song et al.

General Assessment

The authors have provided thorough and rigorous responses to the previous reviews. They have addressed my technical concerns regarding the single-station focal mechanism inversion with extensive additional sensitivity tests and data transparency.

In particular, I commend the authors for performing systematic tests on frequency bands, window time-shifts, 1D velocity models, and incorporating potential SKS phase. Their new ensemble-based quantitative uncertainty analysis demonstrates that the focal mechanism of S0976a is constrained as a steeply dipping strike slip solution with nodal planes oblique to the VM trough system, despite the intrinsic trade-offs of single-station data.

In response to Reviewer 2's comments, the authors have reframed the geodynamic narrative to properly distinguish between inherited tectonic structures and contemporary stress fields. Replacing the simplistic "Tharsis loading" scenario with a combined mechanism of global contraction, Tharsis flexure/loading, and potential dynamic support from mantle upwelling provides a more comprehensive and geodynamically sound context for the inferred strike-slip reactivation.

Overall, I have no major doubts regarding the primary focal mechanism solution. However, several specific technical and interpretative points warrant further clarification and discussion before the manuscript can be considered for publication. I detail these comments below.

We appreciate the reviewer's positive assessment of the revised manuscript. We address the additional technical and interpretive points below and have revised the manuscript accordingly.

Detailed Comments

1. Fig.1: The authors state that "Beachballs show focal mechanisms for Cerberus Fossae events (blue,[35,37,40]) and S0976a (red, this study)." However, not all blue beachballs belong to Cerberus Fossae events, such as S1222a and the two located in the southern highlands. Please revise the caption.

We have corrected the caption for Figure 1. The revised caption reads:

"Blue beachballs show previously published focal mechanisms[35,37,40], whereas the red beachball shows the focal mechanism of S0976a determined in this study."

2. Line 96: "The preferred focal mechanism yields fault strikes of ~60° and ~330°." These values differ from the clustering results (Lines 124–125, 48.0° ± 21.5° and 143.5° ± 22.5°), even accounting for the 180° periodicity. Please check and update for consistency.

We have clarified the distinction between the nodal-plane strikes of the single best-fitting solution and the ensemble-based strike statistics reported later in the manuscript.

The original text:

"The preferred focal mechanism yields fault strikes of ~60° and ~330°."

has been revised to (**lines 95–96** in the revised manuscript):

"The **best-fitting** focal mechanism yields fault strikes of ~60° and ~330°."

3. Fig. 2a: The x-axis label of the bottom right inset figure should be "Back azimuth" instead of "Azimuth".

Revised as suggested.

4. Fig. 2b: While the addition of arrival ticks for the first arriving PP/SS and their depth phases is helpful, identifying individual phase arrivals in the observed waveforms remains challenging. Indeed, synthetics indicate that over such long windows (~45 s for PP and ~75 s for SS), the waveform becomes complex, including triplications, depth phases, and potential

precursors and crustal reverberations (Supplementary Fig. 13). Nevertheless, for all cases, the PP wave is much better fit than the SS wave trains. For example, on transverse component, only the arrival near ~30 s is cleanly matched. This discrepancy appears partly tied to passband selection: in the higher-frequency bands (2.8–6.0 s and 3.6–8.0 s), the transverse SS wave achieves a visually superior fit (Fig. 3d), even though the preferred 3.0–14.0 s band yields the highest overall VR (Fig. 3e). Could the author elaborate more on this?

We have clarified this point in the manuscript. The preferred band is selected because it provides the highest joint variance reduction (VR) across the vertical and horizontal components used to constrain the focal mechanism. The normalised correlation coefficients are 0.93 for PP, 0.74 for radial SS, and 0.59 for transverse SS. For the 3.6–8.0 s and 2.8–6.0 s bands, the corresponding coefficients are 0.83, 0.54, and 0.76, and 0.84, 0.44, and 0.65, respectively. Thus, although the shorter-period bands better reproduce the transverse SS waveform, the preferred passband provides the best simultaneous fit to PP and radial SS.

The apparent discrepancy arises because the shorter-period synthetics reproduce selected early transverse SS arrivals but fit later arrivals of comparable or greater amplitude less well. Because VR measures amplitude residuals over the full fitting windows, these later mismatches reduce the overall VR even when individual phases appear well aligned. We have clarified the basis for the passband selection in the Methods (**lines 354–356**).

We further tested the sensitivity to crustal structure using the velocity model derived from Rayleigh-wave group-velocity dispersion (Kim et al., 2023). Although this model still favours a steeply dipping strike-slip mechanism, it provides a poorer fit to parts of the later PP and SS waveforms, suggesting that intra-crustal velocity contrasts contribute to the observed waveform complexity. We have added this result to the Results (**lines 120–124**), updated Fig. 2 and Supplementary Fig. 12 accordingly, and expanded the Methods (**lines 450–463**) to describe the tested velocity models in greater detail.

5. Following up on the previous point, I wonder whether the better fit to PP over SS stems from different weighting in their misfits. While Line 367 states that equal weighting was assigned to PP and SS, I find that in the inversion script provided by the authors, a higher weight for PP misfit over SS is set (Lines 175–179 in mtuq_script.py):

```
#manually assigned weights
```

weight_P = 31.9557

weight_S = 1.0

weight_ST=weight_S

weight_SR=weight_ST

Applying a higher weight to PP naturally forces the grid search to prioritize PP fitting at the expense of SS. I appreciate the authors for making their code and data publicly available. However, please clarify whether this weighting scheme was applied to all reported inversions. If equal weighting was intended, please discuss how re-balancing the PP/SS weights impacts the fit to horizontal SS components and the resulting focal mechanism ensemble.

We have clarified this point in the Methods. The reviewer appears to have misinterpreted weight_P = 31.9557 as an empirical preference for PP over SS. It is instead an energy-normalisation factor that compensates for the lower waveform energy of PP relative to the combined radial and transverse SS waveforms. Here, $E\_PP = \Sigma d\_PP^2$ is the sum of the squared amplitudes of the processed observed PP waveform over all samples and traces within the PP inversion windows. Similarly, $E\_SS = \Sigma d\_SR^2 + \Sigma d\_ST^2$ is the summed squared amplitude of the radial and transverse SS waveforms within the SS inversion windows. The preferred inversion uses E_SS/E_PP = 31.9557, so the objective is proportional to:

R_PP/E_PP + (R_SR + R_ST)/E_SS,

where R denotes the raw L_2 residual.

Therefore, after normalising the waveforms, PP and the combined SS phase group contribute equally to the joint objective. Applying identical numerical weights without normalisation would cause the higher-energy SS waveforms to dominate. This normalisation procedure was applied consistently to all reported inversions.

The original Methods text:

"Misfit was quantified using an amplitude-normalised L_2 norm, in which each component was weighted by the norm of the observed waveform to ensure equal contribution from PP and SS components."

has been revised to (**lines 367–370** in the revised manuscript):

"Misfit was quantified using an amplitude-normalised L_2 norm. Because the observed PP waveform has lower energy than the radial and transverse SS waveforms, the PP residual was scaled by the SS-to-PP energy ratio, giving PP and SS equal influence on the overall misfit."

6. Lines 131–132: "Although single-station geometry permits some trade-off between back azimuth and fault strike[37], the stable solution ensemble remains confined to western VM." Interestingly, looking at Fig. 2a and Supplementary Fig. 3, the VR exhibits strong sensitivity to epicentral distance but remains relatively flat across a wide range of back azimuths. Notably, when the back azimuth approaches ~110°, one of the nodal-plane strikes in the best-fitting solution becomes nearly subparallel to the VM trough system (Supplementary Fig. 3). If considered in isolation, a single solution at this back azimuth could alter the geological interpretation. However, I appreciate that the authors' overarching conclusions are based on an ensemble that systematically integrates uncertainties in location, velocity structure, and source depth. From a statistical standpoint, the ensemble robustly demonstrates that S0976a is dominated by strike-slip faulting oblique to VM (Supplementary Fig. 4), but explicitly noting this trade-off around 110° BAZ would further enhance the manuscript's transparency.

The reviewer correctly points out that the ensemble solution presented in the main text robustly indicates that S0976a is dominated by strike-slip faulting oblique to VM. The local solution near a back azimuth of 110°, with one VM-parallel nodal plane, is an outlier and should not be interpreted in isolation.

We added the following sentences in the revised manuscript (**lines 131–134**):

"The expected back-azimuth–strike trade-off permits an outlying local best-fit solution with a VM-parallel nodal plane (Supplementary Fig. 3), while the ensemble as a whole consists predominantly strike-slip mechanisms with nodal planes oblique to VM."

7. Fig. 4a: "The orange line marks the inferred E–W to WNW–ESE (~090–100°) orientation of the maximum principal compressive stress associated with Hesperian aged deformation[59]." Lines 219–221: "Kinematic analyses of the Noachian transtensional evolution of VM suggest a broadly E–W-oriented maximum compressive axis of ~090–100°[59], …" Lines

285–286: “the axis of maximum horizontal stress proposed for the Noachian development of the canyon system”.

There’s a discrepancy in the assigned geological age for the ancient stress field. Please check the original source and ensure consistency regarding the geologic epoch (Noachian vs. Hesperian) across the text and Fig. 4a.

We thank the reviewer for noting this point. We have revised the text and Fig. 4a correctly and now use “Hesperian” consistently throughout.

8. Line 20: “…, with a subordinate transtensional component.” Lines 97–99: “…, whereas the tension axis (T axis) is broadly perpendicular to it, reflecting horizontal extension across the canyon combined with shear parallel to its axis.” Lines 257–259: “the alignment of the S0976a T axis with mapped graben and normal fault fabrics near Ius Chasma does not imply pervasive present-day extension across VM.” The authors invoke “a subordinate transtensional component” and suggest that the T axis orientation reflects “horizontal extension across the canyon”, while noting later that this “does not imply pervasive present-day extension”. However, as shown in Fig. 3f, the vast majority of acceptable solutions correspond to a steeply dipping, predominantly pure strike-slip mechanism, which accommodates horizontal shear strain rather than crustal extension. While the horizontal projection of the T axis is indeed perpendicular to the trough, invoking regional “transtension” or “extension across the canyon” without a significant normal-fault component from the resolved mechanism might be a slight overinterpretation.

We agree that the geometric implication of the inferred T-axis orientation alone does not imply pervasive present-day crustal extension or transtension across VM. We have revised the text to address this.

Abstract **lines 20–21** now read: “…under a present-day stress field shaped primarily by long-term planetary cooling, regional Tharsis-related loading, and surface erosion-related local unloading.”

Discussion **lines 268–271**:

"In these interpretations, the alignment of the S0976a T axis with mapped graben and ancient normal-fault fabrics near Ius Chasma implies that present-day crustal extension or transtension across VM is not pervasive."

9. Lines 249–250: "a near strike-slip stress state in which a horizontal σ_1 is imposed primarily by global contraction while σ_3 is locally reduced by Tharsis-related stresses." By citing Andrews-Hanna et al. (2008; ref. 71), the authors now provide a more physically sound model for S0976a. Under Tharsis loading stress, the σ_1 is vertical, which predicts dominant normal faulting. Superimposing planet-wide isotropic horizontal compressive stress from global contraction is essential to rotate σ_1 into the horizontal plane, thereby favoring strike slip on favorably oriented faults. It is worth noting that this combined model is proposed in Andrews-Hanna et al. (2008) to explain the strike slip faulting in regions southwest and northwest of Tharsis from Noachian to Early Amazonian. They explicitly excluded the Coprates region (cited in Fig. 4a for VM paleo-stress) from their global model predictions, stating that "stresses in this region would have been strongly influenced by VM tectonism and cannot be reliably predicted by the model." This raises an intriguing geodynamic question: is the "global contraction + Tharsis loading" model the main explanation of the present-day stress field for S0976a, or does regional tectonism surrounding VM also contribute to shaping the modern E–W to WNW–ESE σ_1 and N–S to NNE–SSW σ_3 orientations?

This is an interesting point. However, given that we only have a single focal mechanism from the region, it is difficult to effectively resolve the modern-day distribution of stress regimes around the Tharsis to determine if and how the stresses change at the VM to infer the possible local contributions to the stress pattern. One potential important local contribution to the present-day stresses in the VM would be the unloading effect of the deep-incised erosion of the VM. Such unloading would decrease the elevated vertical stresses imposed by the Tharsis and thereby result in horizontal compression.

We have revised the Discussion to include this explanation to honour the reviewer's interesting question:

Lines 265–271: "Regional stress perturbations associated with VM tectonic architecture may also influence the local stress field and fault reactivation. Deep canyon incision through surface erosion of VM could have produced an unloading effect which would decrease the

vertical stress and result in horizontal compression in the crust. In these interpretations, the alignment of the S0976a T axis with mapped graben and ancient normal-fault fabrics near Ius Chasma implies that present-day crustal extension or transtension across VM is not pervasive"

We also added a brief statement in the Discussion acknowledging that the relative contributions of the candidate stress sources cannot be uniquely determined (**lines 278–281**).

10. Lines 250–251. I'm not sure whether the phrasing "σ_3 is reduced"/"reducing σ_3" is precise. It is the N–S horizontal stress that is reduced makes it the σ_3 axis.

We have revised the wording to refer explicitly to the reduction in N–S horizontal compressive stress, which defines the σ_3 direction in the resulting stress state.

The original text:

"This confining pressure would tend to suppress tensile opening and favour brittle shear failure, consistent with a near strike-slip stress state in which a horizontal σ_1 is imposed primarily by global contraction while σ_3 is locally reduced by Tharsis-related stresses."

has been revised to (**lines 254–258** in the revised manuscript):

"This confining pressure would tend to suppress tensile opening and favour brittle shear failure, consistent with a near strike-slip stress state in which a horizontal σ_1 is imposed primarily by global contraction, while regional Tharsis-related stresses preferentially reduce N–S horizontal compression, making this the σ_3 direction."

11. Lines 253–254. In response to Reviewer 2, the authors introduced upward buoyant mantle material beneath Tharsis as a candidate mechanism for "reducing σ_3", while retaining Tharsis loading as the main explanation. My understanding is that, both mechanisms, when superimposed onto the global contraction, can produce W–E σ_1 and N–S σ_3 orientation at western VM. Could the authors clarify the theoretical or observational basis for favoring Tharsis lithospheric loading over active mantle dynamic support as the primary regional stress source? Is there a quantitative difference in the magnitude or orientation of the resolved stress tensor between flexural loading versus dynamic mantle upwelling at VM, or should both be treated as co-equal candidates of the stress field?

We have revised the text to avoid implying that the relative contributions of flexural loading and dynamic support are resolved by the present data. We emphasise Tharsis loading and flexure because their effects on the regional stress field have been quantified in published models (Andrews-Hanna, 2012a–c). Dynamic support from buoyant mantle material is supported by recent gravity–topography modelling (e.g., Root et al., 2026), but no corresponding local stress prediction is yet available for western VM. We therefore treat both mechanisms as plausible, while noting that their relative contributions cannot be distinguished from the S0976a focal mechanism alone.

We added the following text at **lines 278–281** in the revised manuscript:

"Although the relative contributions of flexural loading and dynamic support cannot be uniquely determined, the stress orientation recorded by S0976a provides observational evidence against which geodynamic models of their combined influence on the present-day stress field can be tested."

We have also revised the Abstract and Fig. 5 caption to avoid identifying Tharsis lithospheric loading specifically as the primary regional forcing.

References

Andrews-Hanna, J. C. The formation of Valles Marineris: 1. tectonic architecture and the relative roles of extension and subsidence. *J. Geophys. Res. Planets* **117,** E03006 (2012).

Andrews-Hanna, J. C. The formation of Valles Marineris: 2. stress focusing along the buried dichotomy boundary. *J. Geophys. Res. Planets* **117**, E04009 (2012).

Andrews-Hanna, J. C. The formation of Valles Marineris: 3. trough formation through super-isostasy, stress, sedimentation, and subsidence. *J. Geophys. Res. Planets* **117**, E06002 (2012).

Kim, D. *et al.* Structure along the Martian dichotomy constrained by Rayleigh and Love waves and their overtones. *Geophys. Res. Lett.* **50**, e2022GL101666 (2023).

Root, B. *et al.* Describing the global gravity field of Mars with lithospheric flexure and deep mantle flow. *J. Geophys. Res. Planets* **131**, e2024JE008765 (2026).

From Reviewer 2:

I thank the authors for their detailed reply and for addressing my concerns. I think the manuscript has improved substantially compared to the earlier version and I am now happy to recommend publication after some corrections.

We are pleased that the reviewer found the revised manuscript substantially improved.

A general point that deserves some discussion is (especially given that the authors discuss the broad implication of their results for stagnant lid tectonics): Why haven't we seen more quakes originating from Tharsis (or elsewhere)?

As the authors note and show, there is a widespread tectonic fabric in the region and on Mars in general, which, per the authors' argumentation can be easily reactivated by passive global contraction (superimposed on a pre-existing stress field). So, the source of stress (global contraction) is here and the crust is at near-failure criterion, then why a single Tharsis event, and why only a handful of seismic loci on Mars? Does it mean that global contraction is non-isotropic or that the strain rate is exceedingly low? Does it mean that crustal stresses healed nearly everywhere but in a geodynamically long-lived Tharsis? (this still wouldn't explain why there was a single Tharsis event).

We have added a statement to the Discussion to address this point. We do not interpret S0976a as evidence that it is the only seismically active event in Tharsis or western Valles Marineris; rather, it is presently the only event in this region for which the available location and waveform quality permit the focal-mechanism analysis undertaken here.

The locations of most seismic events from InSight remain poorly constrained, particularly for low-magnitude events with low signal-to-noise ratios and substantial contamination from wind and atmospheric pressure noise. Their waveform quality is therefore insufficient for the detailed inversion performed in this study. Recent relocation studies suggest that marsquakes may be more widely distributed than the few well-established source regions, including events in the northern lowlands between the dichotomy boundary and Olympus Mons as well as in the southern highlands (Drilleau et al., 2024; Sun & Tkalcic, 2025). For western Valles Marineris specifically, our aftershock calculation provides an indirect illustration of the observational limitation of InSight: displacement recordings from small events (<Mw 3.0) at the distance of S0976a would rarely exceed ambient noise levels (see Fig. 4b). The absence of

a detected event sequence therefore does not necessarily indicate that no additional small events occurred in the region.

We agree that an extremely low strain rate is a plausible contributor, consistent with the global seismic moment rate estimated by Knapmeyer et al. (2023) from the Marsquake Catalogue. However, the four-year InSight mission may be too short to capture recurrence times associated with such a low Martian seismic moment rate. Spatially variable fault healing, regional stress heterogeneity, or non-uniform loading may also play a role, but the present single-station catalogue is not sufficient to distinguish among these possibilities. We therefore added the following brief statement to the Discussion (**lines 289–292** in the revised manuscript):

"However, the limited number of well-constrained source regions and focal mechanisms may also reflect Mars' low seismic moment rate (Knapmeyer et al., 2023), InSight's short single-station record, and poor detectability of small distant events, rather than an absence of seismicity elsewhere."

Line 46: "its" -> "Valles Marineris" ?

We have replaced:

"Its formation and early deformation…"

with:

"The formation and early deformation of VM…"

Line 80: not clear why there is a "however" here

Agreed. "However" was unnecessary in this sentence and has been removed.

The revised sentence now begins:

"Constraining the source mechanism requires sampling the source radiation pattern…"

Line 141: my earlier comment might have been misunderstood. There is no need to cite 42 here.

We have removed this citation from the sentence.

Fig. 4 is the Rose diagram weighted by fault length?

The rose diagram is not weighted by fault length. Each continuous mapped graben or normal fault was assigned a representative strike and counted once, irrespective of its length. The wedge areas are proportional to the number of mapped features in each orientation bin. We have clarified this in the figure caption.

Line 290-295: I'm not sure I understand the large elastic thickness part. Actually, Tharsis probably has some of the lowest elastic thicknesses and Mars, and should also be relatively warm, as there is evidence for young volcanism in the region (see Hauber https://doi.org/10.1029/2011GL047310). Maybe the authors should clarify what they mean by cold (colder than what?) and large (larger than what?).

We have revised this passage to make the claim more precise and to avoid implying that our inference applies to the thermal and mechanical state of the entire Tharsis province. Our intended inference is more limited: brittle failure at depths of several tens of kilometres indicates that at least part of the lithosphere beneath western VM retains sufficient frictional strength to support differential stress at middle-to-lower crustal depths. We also agree that elastic-thickness estimates vary across Tharsis and depend on location, spatial scale, loading history, and the assumed loading model.

Accordingly, we removed the broad characterisation of the Martian lithosphere as "cold" from the preceding Discussion paragraph and deleted "large elastic thickness" from the list of supporting observations. We also revised the text at **lines 306–310** to read:

"Deep brittle faulting beneath western VM indicates that, at least locally, Martian lithosphere remains mechanically active despite extremely low strain rates. Such behaviour suggests that mechanically strong domains can persist within a laterally heterogeneous stagnant lid, allowing inherited structures to localise later deformation over geological timescales."

We also removed somewhat qualitative terms "cold" and "large" from this paragraph.

References

Drilleau, M. et al. Constraints on lateral variations of Martian crustal thickness from seismological and gravity field measurements. *Geophys. Res. Lett.* **51**, e2023GL105701 (2024).

Knapmeyer, M. et al. The global seismic moment rate of Mars after event S1222a. *Geophys. Res. Lett.* **50**, e2022GL102296 (2023).

Sun, W., & Tkalčić, H. Constraints on the origin of the Martian dichotomy from southern highlands marsquakes. *Geophys. Res. Lett.* **52**, e2024GL110921 (2025).

Review comments on “Active, persistent crustal straining at Valles Marineris” by Song et al.

This manuscript investigates the contemporary seismotectonics of Mars by analyzing the unique marsquake S0976a—the only event detected in the Martian western hemisphere. The authors utilize seismic focal mechanism to constrain present-day crustal deformation at Valles Marineris (VM). They deserve credit for addressing a highly compelling planetary evolution question and for attempting to bridge seismological observations with tectonics. The “paleo-to-modern” comparison is conceptually interesting; exploring whether the contemporary stress field resolved from S0976a aligns with Noachian-aged transtensional stress orientations (~90°–100°) is a valuable approach to evaluating long-term lithospheric stress maintenance on a stagnant-lid planet. Additionally, using the estimated 20–40 km centroid depth to rule out shallow wrinkle-ridge detachments and leveraging the strike-slip focal mechanism to contest traditional models of overthickened crustal gravitational collapse are useful discussion points.

However, the manuscript’s overarching geodynamic narrative hinges entirely on the precise orientation of the resolved P/T axes and the strike-slip nodal planes. Given that these focal parameters are derived from an underdetermined, single-station inversion of free-surface multiples (PP and SS) at a teleseismic distance, I have reservations regarding the accuracy, robustness, and uniqueness of the resolved focal mechanism solution. Single-station focal mechanism inversions on Mars are notorious for severe trade-offs, especially between fault strike and event back-azimuth. If the preferred focal mechanism is affected by minor inversion artifacts or trade-offs, the resulting geologic interpretations regarding selective fault reactivation would be compromised.

This concern about solution accuracy, combined with major issues concerning data reproducibility and frequency band selection, means the manuscript requires necessary modifications and technical justification before it can be considered for publication. Below are my detailed comments, which I hope will assist the authors in improving the rigor and overall quality of their work.

Major comments

1. Choice of frequency band, processing transparency, and reproducibility

The authors filter the vertical component PP and two horizontal component SS waves between 0.08 and 0.2 Hz in their waveform inversion. However, I have reservations regarding the suitability of this specific frequency band, alongside major concerns about data reproducibility and phase utilization.

Horleston et al. (2022) analyzed the spectral characteristics of the S0976a signal and found that “the PP phase includes energy between 2 and 6 s, whereas the SS phase is

both higher in amplitude and wider in frequency, extending from 1 to 8 s". The authors' selected frequency range (0.08–0.2 Hz, or 5–12.5 s) only overlaps with a very small portion of the true signal energy documented for these phases (Fig. R1). By filtering out the higher-frequency energy (2–5 s) where the PP and SS amplitudes are most robust, the inversion might be subject to background noise or artifacts (Fig. R2), thereby severely compromising the reliability of the resulting focal mechanism. The authors need to provide a clearer justification for why this specific range was chosen.

[REDACTED]

Fig. R1. Screenshot of the upper panel of Fig. 4 in Horleston et al. (2022). Shaded blue areas denote the 0.08–0.2 Hz range used in this manuscript.

Here, I filtered the three-component waveforms around the PP and SS arrivals into different frequency bands, and compared body wave energies (Fig. R2). The results show that body wave signal-to-noise ratios are relatively low in the period of 5–12.5 s, compared to those at higher frequencies (3–8 s). Specifically, a prominent low-frequency noise artifact occurs between -30 s and 10 s relative to the PP arrival on the radial component. I suspect this localized noise contamination is the primary reason the authors omitted the radial component of the PP wave from their inversion, while all previous source inversions on Mars include radial-component P-waves to properly constrain the focal mechanism (Brinkman et al., 2021; Jacob et al., 2022; Maguire et al., 2023). From my perspective, the 3–8 s period is a better choice as the synthetic seismograms can be reliably computed down to 2 s. Although 3D heterogeneities may complicate the wavefield in this band, this effect requires further evaluation, as the higher signal-to-noise ratio might provide tighter constraints.

The data utilized to generate my replication plot (Fig. R2) were obtained from the data repository of Li et al. (2022), which provides the deglitched three-component displacement records for event S0976a (https://zenodo.org/records/7252035). The two-pass, second-order Butterworth bandpass filter was applied. However, the resulting waveforms within the 0.08–0.2 Hz band are quite different from those "emergent yet distinct PP and SS phases" displayed in the manuscript Fig. 2b, except for the radial component SS wave (Fig. R3). Notably, my replicated vertical-component PP wave and transverse-component SS wave exhibit much more prominent, higher-frequency wiggles than the smooth phases presented in Fig. 2b. Are these differences the result of specific preprocessing choices? Or are we looking at quite different time windows? For

instance, did the authors implement particular downsampling, apply narrow-window tapering around the phase arrivals, or perform localized polarization filtering to clean the vertical and transverse component waveforms? To ensure transparency and reproducibility, I suggest the authors clarify these exact data processing steps or make their data publicly available.

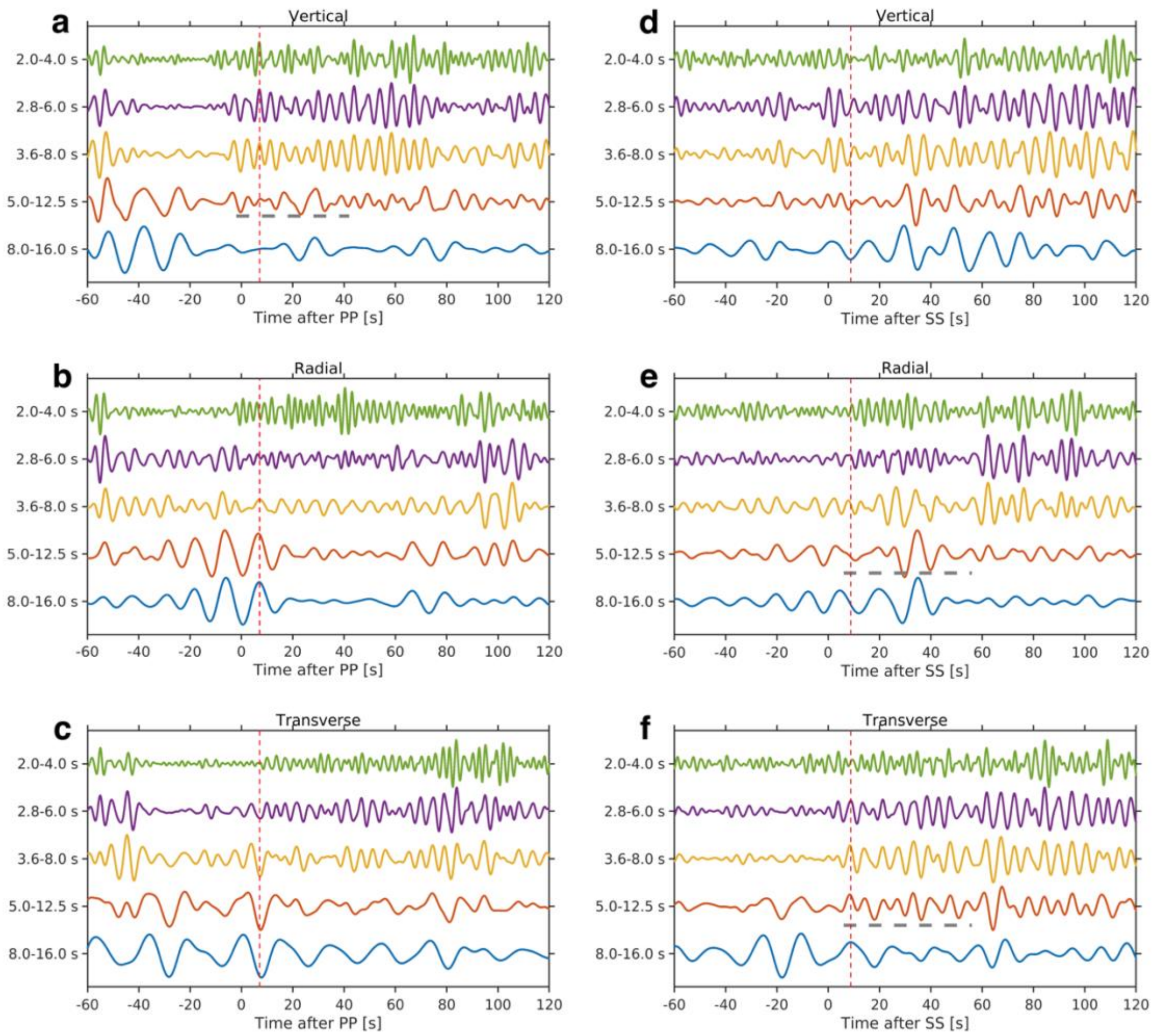

Fig. R2. Three-component waveforms around the PP and SS arrivals filtered into different frequency bands. A back azimuth of 101° is used for waveform rotation (Horleston et al., 2022). The zero times denote the picked PP and SS arrival times in Horleston et al. (2022), while vertical red dashed lines indicate the PP and SS arrival times in Li et al. (2022). Horizontal gray dashed lines represent the selected time windows for waveform comparison with those in Fig. 2b of the manuscript (see Fig. R3).

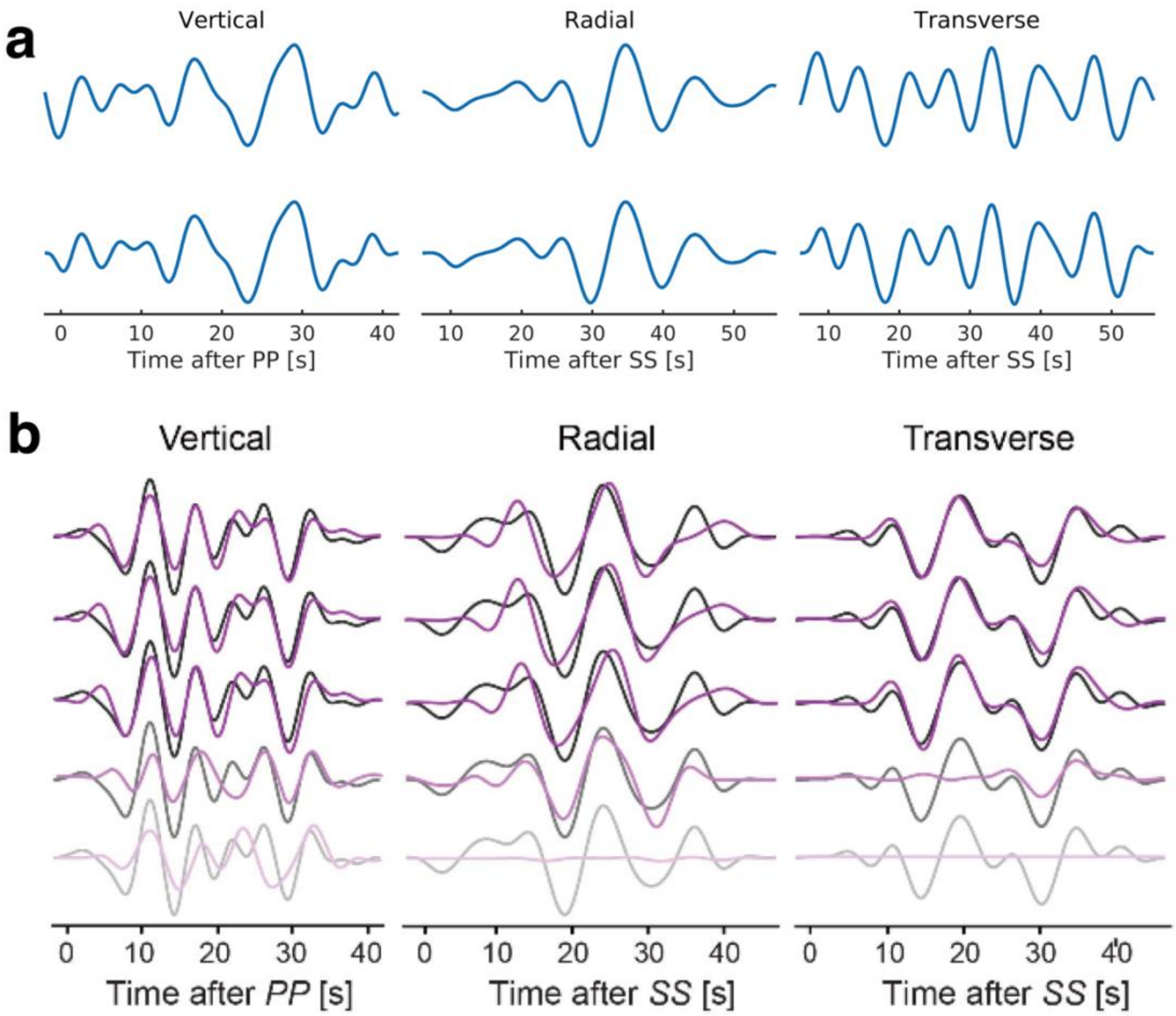

Fig. R3. Waveform comparison of the observed PP and SS waves extracted from my replication pipeline (using the deglitched displacement data from Li et al., 2022) (a) and the corresponding waveforms presented in Fig. 2b of the manuscript (b). The radial component SS wave are quite similar, but the vertical component PP wave and the transverse component SS wave show distinct discrepancies. The lower panels in (a) represent waveforms tapered within a Hanning window with a 10% width at both ends.

For event S0976a, the SKS phase was also identified and picked (Irving et al., 2023). Given that single-station focal mechanism inversions on Mars are severely underdetermined, incorporating additional SKS phase is crucial, as it samples different take-off angle and provides independent constraints on the source radiation pattern. I suggest the authors include the picked SKS phase in their moment tensor inversion to make the resolved focal mechanism more convincing.

2. Lack of rigorous uncertainty quantification for the focal mechanism solution
The manuscript heavily relies on the stable orientation of the P/T axes and the resolved strike-slip nodal planes to interpret the mechanical reactivation of the NE-striking rift-oblique faults. However, single-station body-wave moment tensor inversions on Mars are inherently underdetermined and subject to severe trade-offs between back-azimuth and strike (Supplementary Figs. 4, 6, and 7).

While the authors state that the solution ensemble is “stable” or “tightly clustered”, they do not provide a rigorous, quantitative uncertainty analysis for the focal parameters, especially the strike. Supplementary Fig. 4 shows that there exists a systematic variation between the strike and back azimuth. Considering the substantial location uncertainties inherent to Martian seismic events, particularly regarding single-station back-azimuth determinations, this covariance is deeply concerning. If the nodal plane orientations vary by ±15°–20° within a reasonable confidence interval (e.g., 90% or 95% VR thresholds), the kinematic interpretation could shift dramatically. To strengthen the manuscript’s conclusions, I suggest the authors explicitly quantify these parameter trade-offs and map out the formal confidence bounds of the focal mechanism solutions.

For example, a minor perturbation in the nodal plane orientation could align the rupture plane subparallel to the main E-W/WNW-ESE surface faulting trends. Mechanically, if a nodal plane becomes subparallel to the Tharsis-driven σ_1, the resolved shear stress drops to near-zero, which would completely alter the tectonic narrative proposed in this study. To demonstrate that their structural and geodynamic interpretations are robust against inherent inversion errors, the authors should supply a complete uncertainty and misfit landscape for the focal mechanism parameters.

Minor comments

1. Lines 6–7: In addition to the cited references, Ceylan et al. (2023) investigated Martian seismicity and inferred that three distant events (S0395a, S0133a, and S0154a) may also be situated within the VM region. Given its direct relevance to distant marsquake seismicity, the authors should cite this study here.

2. Lines 34–35: What do the authors mean by the phrase “lithospheric structure of marsquake source regions”? To my understanding, current Martian interior structure models either focus beneath the InSight lander site or represent a broad, large-scale average of the Martian globe. The localized lithospheric structures specifically beneath source regions are generally not resolved independently.

3. Line 72: Please provide the specific value or range of the Moho depth in the source region for comparison.

4. Lines 70–72 and Fig. 2: The authors state in the text that “The best-fitting centroid depth places the source within the mid-crust, at 20–40 km depth”. However, according to Fig. 2, the best-fitting centroid depths vary with the velocity model used, resolving at 30–50 km for the Khan model and 20–40 km for the Samuel model. Because the crustal thickness in the Samuel model is substantially greater than that in the Khan model, these depth ranges imply that the source is located either within the lower crust for the Khan model or within the middle-to-lower crust for the Samuel model (Supplementary Fig. 10). The authors should correct the main text to reflect this model

dependence. Furthermore, are there any geomechanical and rheological implications of these differing structural contexts that warrant discussion?

5. Fig. 2b: Please state explicitly in the figure caption which model (the Khan model or the Samuel model) is used to generate the displayed synthetic waveforms. For cases of “Full”, “CLVD+DC”, and “DC”, the author should also provide the corresponding centroid depth values.

6. Lines 77–82: I’m surprised that small shift in distance would lead to dramatic variation of the focal mechanism. Because a large time shift is explicitly permitted during the waveform fitting process, any minor travel-time discrepancies introduced by a small distance shift should be easily absorbed by the cross-correlation time alignment. If the focal mechanism remains this hyper-sensitive to minor distance variations despite the flexible time shifts, it may imply an instability or non-uniqueness in the inversion. To substantiate this behavior and demonstrate wavefield stability, the authors should include a supplementary figure displaying a record section of synthetic waveforms across a reasonable distance range. In addition, rather than relying on previously determined distance interval of 146° ± 7°, I suggest the authors adopt a distance range calculated directly from the observed SS-PP differential travel time (incorporating its picking uncertainty) with the adopted velocity models (the Khan and Samuel model).

7. Lines 85–86: The cited ref. 45 investigated attenuation parameters within 0.05–0.09 Hz (Onodera et al., 2023), rather than “near 0.5 Hz” as stated in the text. The cited ref. 46 only includes marsquake data recorded before sol 478, meaning the S0976a event analyzed in this study is not part of that catalog (Clinton et al., 2021). Please update the citation to Ceylan et al. (2022).

8. Lines 89–91: The authors should clarify the structural and geomechanical basis for using surface fault density as a direct proxy for the location of a deep-seated marsquake occurring at 20–40 km depth. Because a seismogenic depth of 20–40 km places the source firmly within the mid-to-lower crust, localized surface fault traces may not directly map to the deep stress field or deep-seated fault geometries. The authors should provide a physical justification for this assumption or tone down the text to acknowledge the geometric separation between deep seismic slip and surface structural expressions.

9. Lines 101–102: On the radial component, the synthetic SS waveform generated by the force-based source model exhibits an amplitude similar to the observed data (Fig. 2b).

10. Line 144: The citation for ref. 52 should be moved forward to where the CTX is first introduced in the manuscript (i.e., Line 108).

11. Fig. 3: The abbreviation of CTX should be defined in the main text where it is first

mentioned at Line 108.

12. Fig. 3: “a boulder-fall site” or “boulder-fall sites”?

13. Fig. 3a: The authors should clarify what the circle outlined in black located at approximately 78.4°W/6°S represents, as its definition is missing from both the figure legend and the caption.

14. Line 242: I don’t think ref. 65 (Knapmeyer-Endrun et al., 2021) is an appropriate reference to support the statement regarding “the clustering of marsquakes within localized deformation zones”. The cited study focused fundamentally on constraining the crustal thickness and layering beneath the InSight landing site. It did not investigate marsquake epicentral clustering, spatial seismicity distribution, or active deformation zones. The authors should replace this reference with literature that addresses seismicity on Mars, such as the InSight catalog papers or studies explicitly mapping event distributions.

15. Lines 268–269: The authors should explicitly label the main PP and SS waves, as well as their depth phases, directly on the displayed waveforms. Given that the synthetic waveforms achieve a highly satisfactory fit to the data in Fig. 2b, these phase arrivals can be easily identified and cross-verified using the synthetics. For example, are the large-amplitude arrivals occurring ~30 s after PP and SS the depth phases? If so, explicitly identifying them will significantly improve the readability of the figures and allow readers to clearly trace the key observational constraints.

16. Line 301: Based on the parameter sampling described by the authors, the calculation at each location should be 60 * 60 * 60 * 8 * 50 (corresponding to the number of searched strike, dip, rake, scalar moment, and source depth increments, respectively). This yields 8.64×10^7 total trials, rather than the “5×10^7” stated in the text. Please double-check accordingly.

17. Lines 345–361: I suggest the authors move the section titled “Amplitude-based source analysis” into the Supplementary Information. There appears to be no substantive discussion or corresponding statement interpreting the actual results of this method within the main text.

18. Supplementary Fig. 1: Why the displayed misfit values are so small (on the order of ~1e-12)? Are they normalized to the maximum misfit among all grid-search trials? Regarding the uncertainty analysis requested in the first major comment, could the authors provide the acceptable ranges of the resolved source parameters instead of presenting only a single best-fitting solution? Visualizing how the parameter space behaves near the minimum is critical for assessing potential trade-offs and evaluating the true robustness of the inversion.

19. Supplementary Figs. 6–9: The x-axis label in the right panels should be “Back azimuth” instead of “Azimuth”.

20. Supplementary Fig. 10: Are the waveforms displayed here the best-fitting solutions obtained for each individual model case? If so, why do none of them match the synthetics shown in Fig. 2b, which the authors label as the “best-fitting waveform comparisons”? For example, the negative arrival at ~30 s after PP (a likely depth phase) is quite well fitted in Fig. 2b, yet it shows a clear deficit and is poorly reproduced here when using the Khan and the Samuel models.

21. Supplementary Fig. 13: This figure clearly shows that polarization information is lacking below 0.2 Hz. Consequently, the 0.08–0.2 Hz frequency band used for the waveform inversion may be operating in a regime where particle motions are poorly constrained due to low signal-to-noise ratios or environmental ambient noise. The authors should explain how they accounted for this observational deficit or explicitly discuss how the lack of low-frequency polarization impacts the overall uncertainty and stability of their focal mechanism solution.

References

Brinkman, N., Stähler, S. C., Giardini, D., Schmelzbach, C., Khan, A., Jacob, A., et al. (2021). First Focal Mechanisms of Marsquakes. *Journal of Geophysical Research: Planets, 126*(4), e2020JE006546. https://doi.org/10.1029/2020JE006546

Ceylan, S., Clinton, J. F., Giardini, D., Stähler, S. C., Horleston, A., Kawamura, T., et al. (2022). The marsquake catalogue from InSight, sols 0–1011. *Physics of the Earth and Planetary Interiors, 333*, 106943. https://doi.org/10.1016/j.pepi.2022.106943

Ceylan, S., Giardini, D., Clinton, J. F., Kim, D., Khan, A., Stähler, S. C., et al. (2023). Mapping the Seismicity of Mars With InSight. *Journal of Geophysical Research: Planets, 128*(8), e2023JE007826. https://doi.org/10.1029/2023JE007826

Clinton, J. F., Ceylan, S., van Driel, M., Giardini, D., Stähler, S. C., Böse, M., et al. (2021). The Marsquake catalogue from InSight, sols 0–478. *Physics of the Earth and Planetary Interiors, 310*, 106595. https://doi.org/10.1016/j.pepi.2020.106595

Horleston, A. C., Clinton, J. F., Ceylan, S., Giardini, D., Charalambous, C., Irving, J. C. E., et al. (2022). The Far Side of Mars: Two Distant Marsquakes Detected by InSight. *The Seismic Record, 2*(2), 88-99. https://doi.org/10.1785/0320220007

Irving, J. C. E., Lekić, V., Durán, C., Drilleau, M., Kim, D., Rivoldini, A., et al. (2023). First observations of core-transiting seismic phases on Mars. *Proceedings of the National Academy of Sciences, 120*(18), e2217090120. https://doi.org/10.1073/pnas.2217090120

Jacob, A., Plasman, M., Perrin, C., Fuji, N., Lognonné, P., Xu, Z., et al. (2022). Seismic sources of InSight marsquakes and seismotectonic context of Elysium Planitia, Mars. *Tectonophysics, 837*, 229434. https://doi.org/10.1016/j.tecto.2022.229434

Knapmeyer-Endrun, B., Panning, M. P., Bissig, F., Joshi, R., Khan, A., Kim, D., et al. (2021). Thickness and structure of the martian crust from InSight seismic data. *Science, 373*(6553), 438-443. https://doi.org/10.1126/science.abf8966

Li, J., Beghein, C., McLennan, S. M., Horleston, A. C., Charalambous, C., Huang, Q., et al. (2022). Constraints on the martian crust away from the InSight landing site. *Nature Communications, 13*(1), 7950. https://doi.org/10.1038/s41467-022-35662-y

Maguire, R., Lekić, V., Kim, D., Schmerr, N., Li, J., Beghein, C., et al. (2023). Focal Mechanism Determination of Event S1222a and Implications for Tectonics Near the Dichotomy Boundary in Southern Elysium Planitia, Mars. *Journal of Geophysical Research: Planets, 128*(9), e2023JE007793. https://doi.org/10.1029/2023JE007793

Onodera, K., Maeda, T., Nishida, K., Kawamura, T., Margerin, L., Menina, S., et al. (2023). Seismic Scattering and Absorption Properties of Mars Estimated Through Coda Analysis on a Long-Period Surface Wave of S1222a Marsquake. *Geophysical Research Letters, 50*(13), e2022GL102716. https://doi.org/10.1029/2022GL102716

Assessing whether a planet is geodynamically active today represents one major challenge of planetary science. Before NASA's InSight mission landed on Mars, whether the red planet is geodynamically active today remained unclear, and relied on observational evidence of geologically young lava flows. The InSight SEIS instrument recorded hundreds of marsquakes demonstrating the planet to be (at least regionally) tectonically active, and with the locus of seismic activity being related to the large Cerberus Fossae fissure system located in Elysium Planitia. Surprisingly, only a handful of marsquakes have been detected outside of Elysium Planitia, with a single one being located in the western hemisphere. Assessing the origin of this western hemisphere quake, and particularly whether it is related to surface (impact, landslide) or interior (geodynamic) processes would provide important information on Mars' tectonic activity.

In this work, the authors perform a detailed seismic investigation of the single western hemisphere marsquake recorded by InSight. In agreement with previous work, the quake was found to originate from the large Valles Marineris (VM) fissure system. Synthetic and inversion models are presented to characterize the seismic source of the quake, which is found to be consistent with a strike-slip reactivation of pre-existing faults. Surface sources including impacts and landslides are demonstrated to be inconsistent with the data. The authors then suggest that the quake is related to lithospheric stresses generated early in Mars' evolution, and argue that such stresses can be preserved in the crust for billions of years.

In general, I find the manuscript well written. Importantly, I note that I am not a seismologist and have little expertise with analyses of InSight-detected marsquakes. Although the inversion methodology and marsquake-specific results seemed sound, I emphasize that I don't have the expertise to have a detailed critical outlook on these aspects. However, and as further described below, I have some major concerns regarding the (often unclear) authors' interpretations of the source of stresses. In addition, I believe the authors largely inflate the conclusion of their work, which "indicate that lithospheric stresses generated early in planetary evolution can persist over billions of years on a stagnant-lid planet". Across planetary bodies, faults are known to reactivate over billions of year and paleo-stress fields are also known to affect fault patterns. One important aspect of the work that was not sufficiently explored is related to the origin of the present-day stress that triggered the quake. A very simple figure and discussion explain that Tharsis could be the source, but further consideration (uplift? subsidence?) is lacking (see below). **For these reasons, I recommend the paper be returned for major revisions.**

The review is organized as follows: first, I list my major concerns and then provide minor line-numbered comments. All must be addressed thoroughly.

Major concerns:

1. There is a critical unclear aspect of the work and a potential important misconception regarding early stresses, such as in statement like these (found throughout the text):

A. Abstract: "*stresses generated during early planetary evolution remain capable of driving present-day tectonics has not been directly tested*": This is very unclear. Stress is the measure of an external force acting over an area. If the external force is still ongoing, stress can build up leading to tectonic activity (providing the stresses exceeded a critical threshold). One cannot "generate" a stress early, stop it, and then get some tectonic activity (as suggested by this sentence). Maybe the authors mean "initiated" and not "generated"? The crust can however preserve a "memory" of paleostresses that can be reactivated by a new active stress period. But this doesn't mean the new active stress is related to the early stress. Note also that previous work, for example Klimczak et al. (2025) [https://www.sciencedirect.com/science/article/abs/pii/S0012821X2500130X] has shown indicating that Mercury's global contraction has reactivated a tectonic fabric inherited from past stress events (e.g., basin formation). Re-activation of faults has also been extensively covered in the literature (e.g., Woodley et al., (2024); 10.1029/2023GL107757; Andrews-Hanna et al. (2008); https://doi.org/10.1029/2007JE002980. and many others).

B. Line 9 (also Line 11): "*but the alternative hypothesis is that stresses generated early in planetary evolution are stored within the lithosphere and released episodically*": As noted above, this is also unclear. One cannot release stresses episodically without a driving force. Early stresses would be stored in the crust up to some failure threshold (e.g., Byerlee for friction) and cannot be released. However, any subsequent stress on a crust that is already at a failure threshold would generally lead to strain.

C. Line 46: "*likely driven by inherited lithospheric stresses*" Inherited lithospheric stresses cannot drive tectonic activity. It can affect the local activity, providing another external acting stress exists.

2. One important aspect of the work relates to the secondary component of the deformation. Strike slip is interpreted to be dominant, but the authors suggest (Line 68) some extension, indicating transtension. I think this aspect needs to be discussed further. A compressional component would generally be consistent with a globally cooling Mars, where stresses are stored passively over time. Some extension would instead require a more exotic stress source. Depending on the rigidity of the lithosphere, extension at VM could be associated with ongoing Tharsis subsidence (downward directed load). Interestingly, it could also be related to uplift in the Tharsis plateau (upward directed load), which could be associated with some ongoing magmatic activity (a plume?). I think this aspect would be very important and interesting to dive in. I see that the authors provide a very simple story of Tharsis loading (Figure 4), but more quantitative estimates are critically lacking.

3. I am not sure I understand the title of the paper. Crustal strain at VM is observed and it must be persistent. Activity on the other hand is important to note.

Minor concerns:

Abstract: “wane as lithospheres cool and thicken”: This is not always true, as planets cool they also record substantial contraction (from shrinking) that will cause tectonic activity.

Abstract: “*stresses generated early in planetary evolution can persist over billions of years on a stagnant-lid planet. Our results suggest that tectonic activity on rocky planets without plate tectonics may persist longer than previously assumed, reflecting their contemporary thermal states.*”: See my comment above regarding the fact that this work “suggests” [it has been shown already in the past!].

Line 5: “*activity typically decreases as the lithosphere cools and thickens*” Please see the first minor comment above.

Line 7: “*deformation continues well beyond the main phase of planetary cooling*” same as above

Line 22: “*primary*” -> “primordial”?

Line 65: Here the authors discuss and provide only the best-fit. But looking at Fig S1, the misfit seems rather flat for some parameters (e.g. dip going to 60°? Strike to 160? Slip of ±20°?).

Fig S2: It is great that the best-fit reproduce the data. But what about the sensitivity of the inversion to specific parameters? It would be great to show a Figure similar to Fig S1 for this test case.

Line 94: Small graben above wrinkle ridges have been observed on Mercury, indicating that extension can also occur at wrinkle ridges [https://www.nature.com/articles/s41561-023-01281-5].

Line 188: Please note that there is a debate regarding the origin of wrinkle ridges. For example, work by Montési & Zuber (2003; https://agupubs.onlinelibrary.wiley.com/doi/10.1029/2002JE001974) argue for deep-seated wrinkle ridges.

Line 200: Ref 61 must be a mis-citation here. Maybe Plesa et al. (2018) [https://agupubs.onlinelibrary.wiley.com/doi/full/10.1002/2017GL076124] would be more adequate here. Actually, present-day mantle convection does lead to non-negligible convective stresses. I also note that recent work by Root et al. 2026 [https://agupubs.onlinelibrary.wiley.com/doi/full/10.1029/2024JE008765] argues for the existence of a present-day large plume below Tharsis, which would generate surface uplift and stresses.

Line 203: I disagree here. If there is a pre-existing tectonic fabric, global contraction could reactivate faults, leading to strike-slip.

Line 212: The link between faulting and strength is unclear.

Line 230: I think it is possible to have a homogeneous stress field leading to a heterogeneous strain, providing the crust is fractured (see discussion above).

Line 236: I don't think strain rates are constrained by this work. They could very well be higher than expected.

Line 240: I don't think the ref 19 discusses this, though I could be wrong. In addition, the way viscoelastic relaxation affects fault reactivation is not as trivial as explained here. As soon as a fault is formed, it can be reactivated. One way to prevent this reactivation is (I think), if the fault itself gets substantially deformed by relaxation, but this is probably tricky to model.

Review comments on “Active crustal straining and fault reactivation at Valles Marineris” by Song et al.

General Assessment

The authors have provided thorough and rigorous responses to the previous reviews. They have addressed my technical concerns regarding the single-station focal mechanism inversion with extensive additional sensitivity tests and data transparency.

In particular, I commend the authors for performing systematic tests on frequency bands, window time-shifts, 1D velocity models, and incorporating potential SKS phase. Their new ensemble-based quantitative uncertainty analysis demonstrates that the focal mechanism of S0976a is constrained as a steeply dipping strike slip solution with nodal planes oblique to the VM trough system, despite the intrinsic trade-offs of single-station data.

In response to Reviewer 2’s comments, the authors have reframed the geodynamic narrative to properly distinguish between inherited tectonic structures and contemporary stress fields. Replacing the simplistic “Tharsis loading” scenario with a combined mechanism of global contraction, Tharsis flexure/loading, and potential dynamic support from mantle upwelling provides a more comprehensive and geodynamically sound context for the inferred strike-slip reactivation.

Overall, I have no major doubts regarding the primary focal mechanism solution. However, several specific technical and interpretative points warrant further clarification and discussion before the manuscript can be considered for publication. I detail these comments below.

Detailed Comments

1. Fig.1: The authors state that “Beachballs show focal mechanisms for Cerberus Fossae events (blue,[35,37,40]) and S0976a (red, this study).” However, not all blue beachballs belong to Cerberus Fossae events, such as S1222a and the two located in the southern highlands. Please revise the caption.

2. Line 96: “The preferred focal mechanism yields fault strikes of ~60° and ~330°.” These values differ from the clustering results (Lines 124–125, 48.0° ± 21.5° and 143.5° ± 22.5°), even accounting for the 180° periodicity. Please check and update for consistency.

3. Fig. 2a: The x-axis label of the bottom right inset figure should be “Back azimuth” instead of “Azimuth”.

4. Fig. 2b: While the addition of arrival ticks for the first arriving PP/SS and their depth phases is helpful, identifying individual phase arrivals in the observed waveforms remains challenging. Indeed, synthetics indicate that over such long windows (~45 s for PP and ~75 s for SS), the waveform becomes complex, including triplications, depth phases, and potential precursors and crustal reverberations (Supplementary Fig. 13). Nevertheless, for all cases, the PP wave is much better fit than the SS wave trains. For example, on transverse component, only the arrival near ~30 s is cleanly matched. This discrepancy appears partly tied to passband selection: in the higher-frequency bands (2.8–6.0 s and 3.6–8.0 s), the transverse SS wave achieves a visually superior fit (Fig. 3d), even though the preferred 3.0–14.0 s band yields the highest overall VR (Fig. 3e). Could the author elaborate more on this?

5. Following up on the previous point, I wonder whether the better fit to PP over SS stems from different weighting in their misfits. While Line 367 states that equal weighting was assigned to PP and SS, I find that in the inversion script provided by the authors, a higher weight for PP misfit over SS is set (Lines 175–179 in mtuq_script.py):

> *#manually assigned weights*
> *weight_P = 31.9557*
> *weight_S = 1.0*
> *weight_ST=weight_S*
> *weight_SR=weight_ST*

Applying a higher weight to PP naturally forces the grid search to prioritize PP fitting at the expense of SS. I appreciate the authors for making their code and data publicly available. However, please clarify whether this weighting scheme was applied to all reported inversions. If equal weighting was intended, please discuss how re-balancing the PP/SS weights impacts the fit to horizontal SS components and the resulting focal mechanism ensemble.

6. Lines 131–132: "Although single-station geometry permits some trade-off between back azimuth and fault strike[37], the stable solution ensemble remains confined to western VM." Interestingly, looking at Fig. 2a and Supplementary Fig. 3, the VR exhibits strong sensitivity to epicentral distance but remains relatively flat across a wide range of back azimuths. Notably, when the back azimuth approaches ~110°, one of the nodal-plane strikes in the best-fitting solution becomes nearly subparallel to the VM trough system (Supplementary Fig. 3). If considered in isolation, a single solution at this back azimuth could alter the geological interpretation. However, I appreciate that the authors' overarching conclusions are based on an ensemble that systematically integrates uncertainties in location, velocity structure, and source depth. From a statistical standpoint, the ensemble robustly demonstrates that S0976a is dominated by strike-slip faulting oblique to VM (Supplementary Fig. 4), but explicitly noting this trade-off around 110° BAZ would further enhance the manuscript's transparency.

7. Fig. 4a: "The orange line marks the inferred E–W to WNW–ESE (~090–100°)

orientation of the maximum principal compressive stress associated with Hesperian-aged deformation[59].” Lines 219–221: “Kinematic analyses of the Noachian transtensional evolution of VM suggest a broadly E–W-oriented maximum compressive axis of ~090–100°[59], …” Lines 285–286: “the axis of maximum horizontal stress proposed for the Noachian development of the canyon system”.
There’s a discrepancy in the assigned geological age for the ancient stress field. Please check the original source and ensure consistency regarding the geologic epoch (Noachian vs. Hesperian) across the text and Fig. 4a.

8. Line 20: “…, with a subordinate transtensional component.” Lines 97–99: “…, whereas the tension axis (T axis) is broadly perpendicular to it, reflecting horizontal extension across the canyon combined with shear parallel to its axis.” Lines 257–259: “the alignment of the S0976a T axis with mapped graben and normal fault fabrics near Ius Chasma does not imply pervasive present-day extension across VM.”
The authors invoke “a subordinate transtensional component” and suggest that the T axis orientation reflects “horizontal extension across the canyon”, while noting later that this “does not imply pervasive present-day extension”. However, as shown in Fig. 3f, the vast majority of acceptable solutions correspond to a steeply dipping, predominantly pure strike-slip mechanism, which accommodates horizontal shear strain rather than crustal extension. While the horizontal projection of the T axis is indeed perpendicular to the trough, invoking regional “transtension” or “extension across the canyon” without a significant normal-fault component from the resolved mechanism might be a slight overinterpretation.

9. Lines 249–250: “a near strike-slip stress state in which a horizontal σ_1 is imposed primarily by global contraction while σ_3 is locally reduced by Tharsis-related stresses.” By citing Andrews-Hanna et al. (2008; ref. 71), the authors now provide a more physically sound model for S0976a. Under Tharsis loading stress, the σ_1 is vertical, which predicts dominant normal faulting. Superimposing planet-wide isotropic horizontal compressive stress from global contraction is essential to rotate σ_1 into the horizontal plane, thereby favoring strike slip on favorably oriented faults. It is worth noting that this combined model is proposed in Andrews-Hanna et al. (2008) to explain the strike slip faulting in regions southwest and northwest of Tharsis from Noachian to Early Amazonian. They explicitly excluded the Coprates region (cited in Fig. 4a for VM paleo-stress) from their global model predictions, stating that “stresses in this region would have been strongly influenced by VM tectonism and cannot be reliably predicted by the model.” This raises an intriguing geodynamic question: is the “global contraction + Tharsis loading” model the main explanation of the present-day stress field for S0976a, or does regional tectonism surrounding VM also contribute to shaping the modern E–W to WNW–ESE σ_1 and N–S to NNE–SSW σ_3 orientations?

10. Lines 250–251. I’m not sure whether the phrasing “σ_3 is reduced”/“reducing σ_3” is precise. It is the N–S horizontal stress that is reduced makes it the σ_3 axis.

11. Lines 253–254. In response to Reviewer 2, the authors introduced upward buoyant mantle material beneath Tharsis as a candidate mechanism for "reducing σ_3", while retaining Tharsis loading as the main explanation. My understanding is that, both mechanisms, when superimposed onto the global contraction, can produce W–E σ_1 and N–S σ_3 orientation at western VM. Could the authors clarify the theoretical or observational basis for favoring Tharsis lithospheric loading over active mantle dynamic support as the primary regional stress source? Is there a quantitative difference in the magnitude or orientation of the resolved stress tensor between flexural loading versus dynamic mantle upwelling at VM, or should both be treated as co-equal candidates of the stress field?

I thank the authors for their detailed reply and for addressing my concerns. I think the manuscript has improved substantially compared to the earlier version and I am now happy to recommend publication after some corrections.

A general point that deserves some discussion is (especially given that the authors discuss the broad implication of their results for stagnant lid tectonics): Why haven't we seen more quakes originating from Tharsis (or elsewhere)?

As the authors note and show, there is a widespread tectonic fabric in the region and on Mars in general, which, per the authors' argumentation can be easily reactivated by passive global contraction (superimposed on a pre-existing stress field). So, the source of stress (global contraction) is here and the crust is at near-failure criterion, then why a single Tharsis event, and why only a handful of seismic loci on Mars? Does it mean that global contraction is non-isotropic or that the strain rate is exceedingly low? Does it mean that crustal stresses healed nearly everywhere but in a geodynamically long-lived Tharsis? (this still wouldn't explain why there was a single Tharsis event).

Line 46: "its" -> "Valles Marineris" ?

Line 80: not clear why there is a "however" here

Line 141: my earlier comment might have been misunderstood. There is no need to cite 42 here.

Fig. 4 is the Rose diagram weighted by fault length?

Line 290-295: I'm not sure I understand the large elastic thickness part. Actually, Tharsis probably has some of the lowest elastic thicknesses and Mars, and should also be relatively warm, as there is evidence for young volcanism in the region (see Hauber https://doi.org/10.1029/2011GL047310). Maybe the authors should clarify what they mean by cold (colder than what?) and large (larger than what?).
